# Rapid Synthesis of the Spiroketal Subunit of Neaumycin B: Stereochemical Aspects of Singly Anomeric Spiroketals and Proposal for a Stereocenter Reassignment

**DOI:** 10.1021/acs.orglett.4c03751

**Published:** 2024-12-09

**Authors:** Anna E. Healy, Marcus D. Van Engen, Nicholas A. Cinti, Paul E. Floreancig

**Affiliations:** Department of Chemistry, University of Pittsburgh, Pittsburgh, Pennsylvania 15260, United States

## Abstract

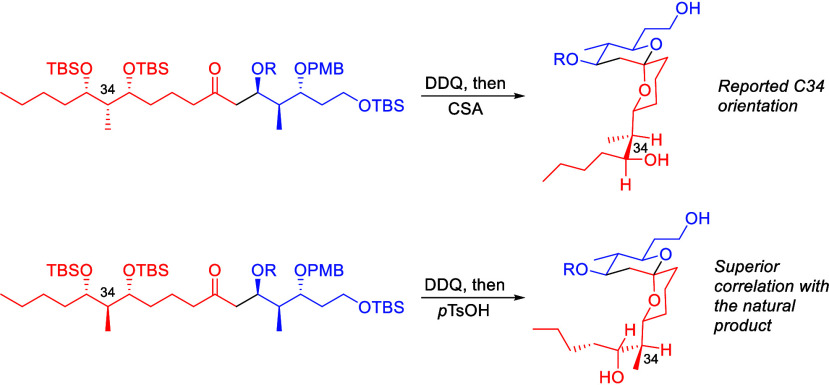

Neaumycin B is a complex polyketide that shows phenomenal cytotoxicity against U87 glioblastoma cells. The singly anomeric spiroketal core is a notable subunit in the natural product’s structure. We report a rapid and convergent approach to the spiroketal group, resulting in the formation of two isomeric singly anomeric spiroketals. We describe the origin of this rarely described outcome in spirocycle formation and subsequently propose a correction to a stereocenter in the natural product.

Simone and co-workers isolated the macrocyclic polyketide neaumycin B (**1**) in 2015 from the microspore *Actinoplanes* sp. ATCC33076 and showed that it enhanced the antibiotic efficacy of ramoplanin toward MRSA.^1^ This report described the atomic connectivity of the natural product but contained no stereochemical information. Fenical, Jensen, and co-workers subsequently isolated **1** from a *Micromonospora* sp. from the brown alga *Stypodium zonale* and identified its biosynthetic gene cluster.^2^ This group conducted extensive NMR studies and correlated the results with information from the genomic data, resulting in the proposed stereoisomer shown in Figure 1. Moreover the authors reported that neaumycin B shows exceptional and selective cytotoxic potency toward U87 glioblastoma cells. Unfortunately the chemical instability of **1** limits access from natural sources. The biological activity, low natural abundance, and synthetic challenge make neaumycin B an attractive target for chemical synthesis. Two total syntheses of the putative structure have been reported for the initially reported structure^3^ and several subunit syntheses have also appeared in the literature.^4^ The spectral data from the total syntheses, while seeming to confirm the atomic connectivity, did not match those of the reported structure, indicating that the actual stereostructure of neaumycin B remains a mystery. Additionally the reported structure lacks cytotoxic activity.^3a^ Solving the mystery of the structure of **1** therefore requires further synthetic efforts, and is essential for further studies of its antiglioblastoma activity.

**Figure 1:**
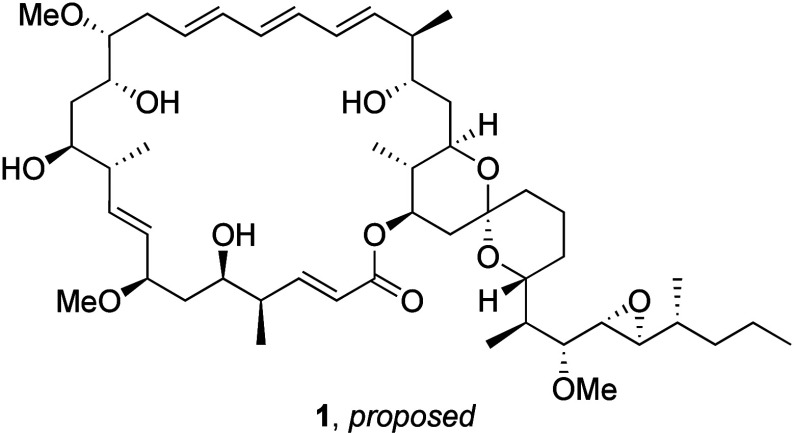
Proposed structure of neaumycin B.

The spiroketal is a notable structural feature of **1**. The close agreement of the spectral data for this portion of the molecule from the isolation efforts and synthetic materials in the total syntheses indicate that this structural unit correlates with singly anomeric structure **2** rather than the doubly anomeric isomer **3** (Figure 2).^5^ This manuscript describes a rapid and convergent approach to the spiroketal unit in which a Mukaiyama aldol serves as the key fragment coupling step. An examination of the stereochemical outcome of the spirocyclization showed that, while two stereoisomers were formed, none of the doubly anomeric isomer corresponding to **3** was isolated. The origin of the spiroketal stereoisomers and key spectroscopic observations are described. During the course of this investigation we noted a clear stereochemical misassignment from the original paper at a stereocenter that is peripheral from the spiroketal and propose an alternative structure that more accurately fits the spectral data from the natural product.

**Figure 2:**
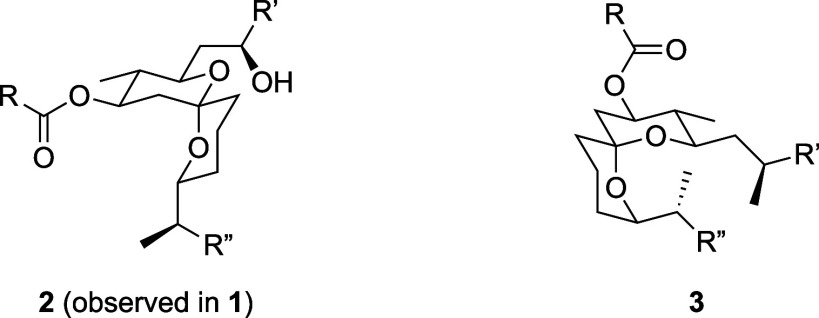
Observed spiroketal geometry and a doubly anomeric stereoisomer.

Our initial interest in the spiroketal unit arose from a desire to expand our hetero Diels–Alder/carbon–hydrogen bond cleavage-based convergent spiroketal formation^6^ to the formation of singly anomeric structures. The inability to conduct stereocontrolled the hetero Diels–Alder reaction on this system led us to change the approach. Our revised route employs a dehydrative spiroketal formation from a substrate that arises from a stereoselective aldol fragment-coupling step (Scheme 1). Setting a product-relevant stereocenter in the fragment-coupling process defines an attractive element of this strategy.

**Scheme 1:**
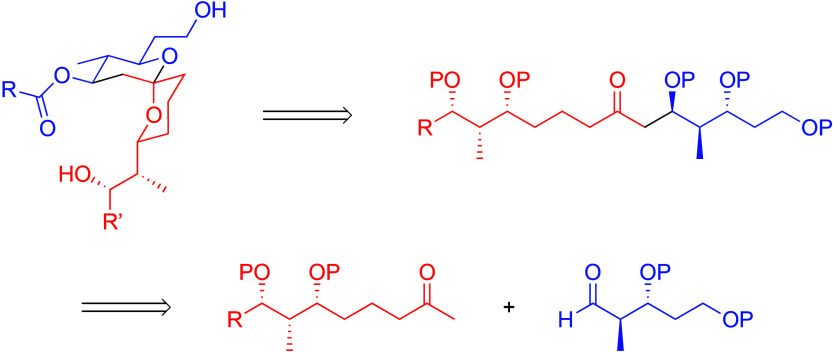
Retrosynthesis of the Spiroketal Unit

The synthesis of the aldehyde fragment is shown in Scheme 2. Exposing aldehyde **4**, readily available in two steps from 1,3-propanediol, to Leighton’s *in situ* crotylation conditions^7^ produced **5** in 68% yield and 95% ee. A minor desilylation product forms during the workup, but the resulting alcohol can be reprotected to enhance material throughput. The protecting group selection for the resulting hydroxy group proved to be crucial to the success of the downstream aldol reaction, with the *p*-methoxybenzyl group being optimal. Exposing **5** to *p*-methoxybenzyl trichloroacetimidate^8^ in the presence of Sc(OTf)_3_ provided ether **6** in good yield. The sequence concluded with a Johnson-Lemieux oxidation^9^ to form aldehyde **7**.

**Scheme 2:**
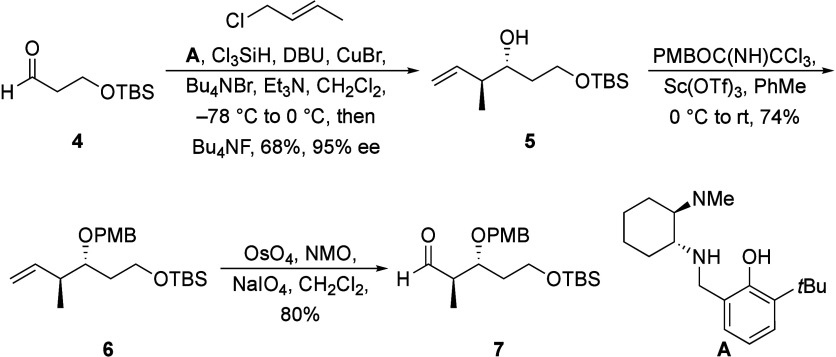
Synthesis of the Aldehyde Fragment

The construction of the ketone fragment (Scheme 3) commenced with the enantio- and diastereoselective cycloaddition of aldehyde **8**, prepared through a Swern oxidation of 5-hexen-1-ol, with propionyl chloride to form β-lactone **9**.^10^ This step can be conducted on multigram scale with minimal waste generation and no detectable stereoisomer formation. Lactone opening to form the Weinreb amide followed by the addition of excess *n*BuLi formed ketone **10**. Selective *syn*-reduction of the ketone proceeded with NaBH_4_ and Et_2_BOMe,^11^ and silylation of the resulting diol with TBSOTf provided **11**. The Tsuji variation^12^ of the Wacker oxidation to **11** produced ketone **12**.

**Scheme 3:**
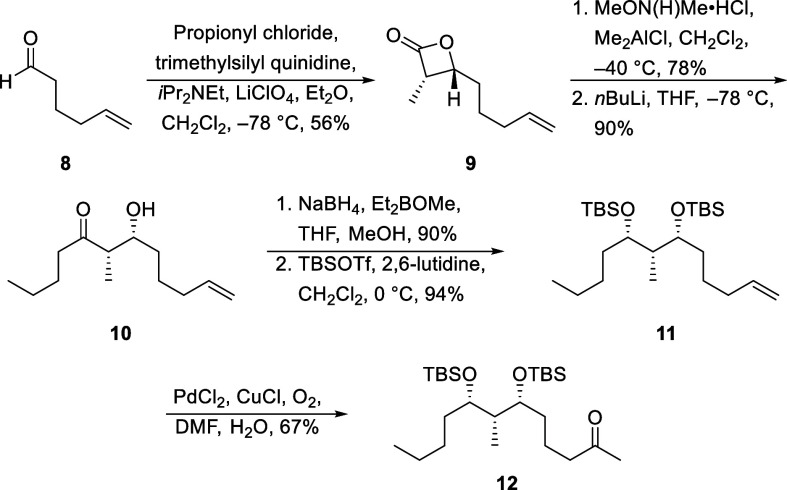
Synthesis of the Ketone Fragment

We chose to connect the fragments through a Mukaiyama aldol reaction (Scheme 4) because this strategy offers the greatest opportunity to control the stereocenter of the resulting alcohol. The stereocenters in aldehyde **7** provide the opportunity for matched stereoinduction through Felkin control from the α-position and Evans’ dipole minimization model from the β-position.^13^ Conversion of **12** to enolsilane **13** proceeded efficiently in the presence of LDA and TMSCl. Exposing **13** and **7** to BF_3_·OEt_2_ resulted in the formation of aldol products as an 8:1 mixture of diastereomers, with an isolated yield of 51% for **14**. Acylation proceeded under standard conditions to form **15**, in which the *p*-nitrobenzoate was employed with the (unrealized) expectation that it would facilitate crystallization. The PMB ether was cleaved by DDQ in a neutral buffer to form hemiketal **16**. The instability of this compound did not allow for complete characterization, but the closed form of the hydroxy ketone was the only visible structure by ^1^H NMR. Treatment of **16** with CSA in MeOH rapidly resulted in deprotection and cyclization to form a separable 2:1 mixture of spirocycles. These species were individually peracylated to facilitate structure determination, delivering **17** and **18** in 95% and 87% yields, respectively.

**Scheme 4:**
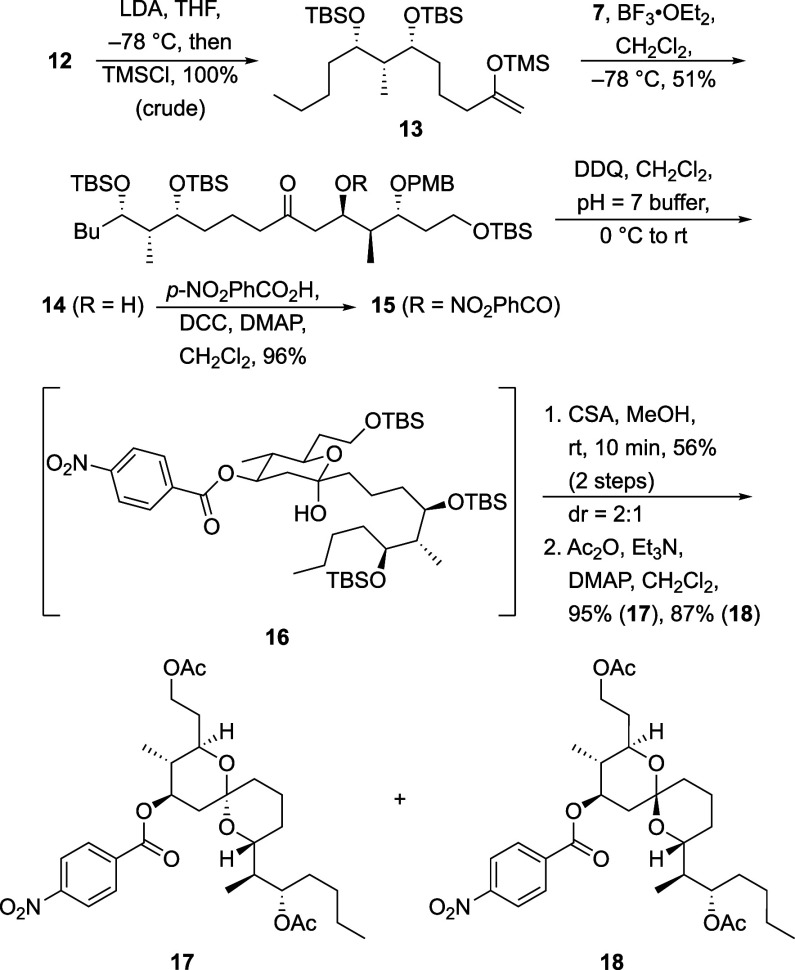
Spirocycle Synthesis

Analyzing **17** and **18** by ^1^H NMR and NOESY experiments revealed that many of the key hydrogens exhibited very similar splitting patterns and coupling constants, but significantly different chemical shifts and NOE patterns (Table 1, neaumycin B numbering). Splitting pattern similarity clearly showed that all substituents on the spiroketal unit are in equatorial alignments. This disqualifies the possibility that one of the products was a doubly anomeric isomer related to structure **3** in Figure 2. The major product in this sequence (**17**) showed chemical shifts and NOEs that closely matched those of the natural product, either from synthesis or isolation. We realized that the minor product was consistent with the alternative singly anomeric isomer **18**. The oxygen of the more substituted tetrahydropyran ring of **17** is in a nonanomeric orientation with respect to the less substituted ring, while the opposite is observed in **18**.

**Table 1:**
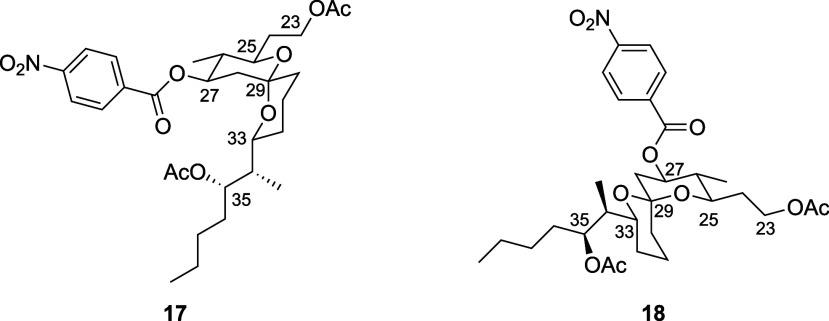
Product Analysis.^a^

Hydrogen	**17** δ, *J*	**18** δ, *J*	**17** NOE	**18** NOE
23	4.43 (m)	4.33 (dd)	25	25, 31
		7.6, 5.8		
25	4.12 (ddd)	2.95 (td)	23, 27	23, 27
	10.2, 9.2, 2.5	9.2, 2.4		
27	5.10 (td)	4.92 (td)	25, 33	25
	16.2, 4.5	11.0, 4.9		
28 (eq)	2.83 (dd)	2.16 (dd)	27, 33	27
	12.7, 4.4	12.2, 4.9		
33	3.63 (ddd)	4.33 (dd)	28 (eq), 35	23, 35
	11.2, 7.0, 1.9	11.8, 4.7, 1.9		
35	5.33 (ddd)	5.35 (ddd)	33	33
	8.5, 4.6, 4.6	7.0, 5.6, 5.4		

a Spectra were recorded in C_6_D_6_ at 500 MHz. Chemical shifts (δ) are reported in ppm, and coupling constants (J) are reported in Hz.

A notable difference in the chemical shifts for the compounds is seen in the hydrogens that have a diaxial relationship with an oxygen atom. The hydrogens on C25 and C27 in **17** are downfield relative to the corresponding hydrogens in **18** by 1.17 and 0.18 ppm, respectively, while the C33 hydrogen in **17** is upfield relative to the corresponding hydrogen in **18** by 0.7 ppm. The equatorial hydrogen on C28 is also a strong indicator of the stereoisomer, showing a 0.67 ppm downfield chemical shift in **17** relative to **18**. The origin of the anomalously downfield shift in **17** (which is also seen in the natural product) is unclear, though we note that this hydrogen has a *gauche*-relationship with an anomerically oriented oxygen. A similar effect was observed for **16** (see Supporting Information for details), indicating that this effect might be general. These spectral differences should be valuable starting points for distinguishing spiroketal structures in future endeavors.

Spirocyclization leads to the apparent formation of only one stereocenter, leading to questions of how more than two stereoisomers are possible. This occurs because the spiro[5.5.1] ring system is axially chiral and, therefore, two stereogenic units are formed in the spirocyclization process. Spirocycles **17** and **18** differ in the orientation of the spirocycle stereocenter and the axial orientation of the spirocycle. The dianomeric stereoisomers were not observed, presumably due to the energetic penalty associated with axially oriented branched chains. The isolation of two singly nonanomeric spiroketals was noted in syntheses of spongistatin,^14^ but is not discussed in detail and is otherwise seldom reported. This illustrates the potential to form more than two spiroketal isomers when the doubly anomeric stereoisomer would contain an axial substituent.

The structural similarity of compounds **17** and **18** indicate that they should be approximately equivalent energetically, which is consistent with their being formed as a 2:1 mixture. Exposing pure **18** to the spirocyclization conditions resulted in a slow conversion to a 2:1 mixture of **17** and **18**, in accord with their predicted energetic similarity. The equilibration is far slower than the cyclization, however, suggesting that the mixture arises from kinetic control. We postulate the stereoisomers are generated from **16** through as a kinetically controlled mixture through different pathways (Scheme 5). Pathway A proceeds through ionization to form oxocarbenium ion **19**, which delivers **20** by alcohol addition through a stereoelectronically preferred axial trajectory, with a net retention of stereochemistry at the anomeric position. The alternate pathway requires stereochemical inversion at the anomeric center. This can be achieved through Pathway B, in which spirocyclization occurs either through an S_N_2-pathway or an S_N_1-pathway with stereocontrol arising from an intermediate in which the water is still electrostatically associated with the oxocarbenium ion (**21**),^15^ generating **22**.

**Scheme 5:**
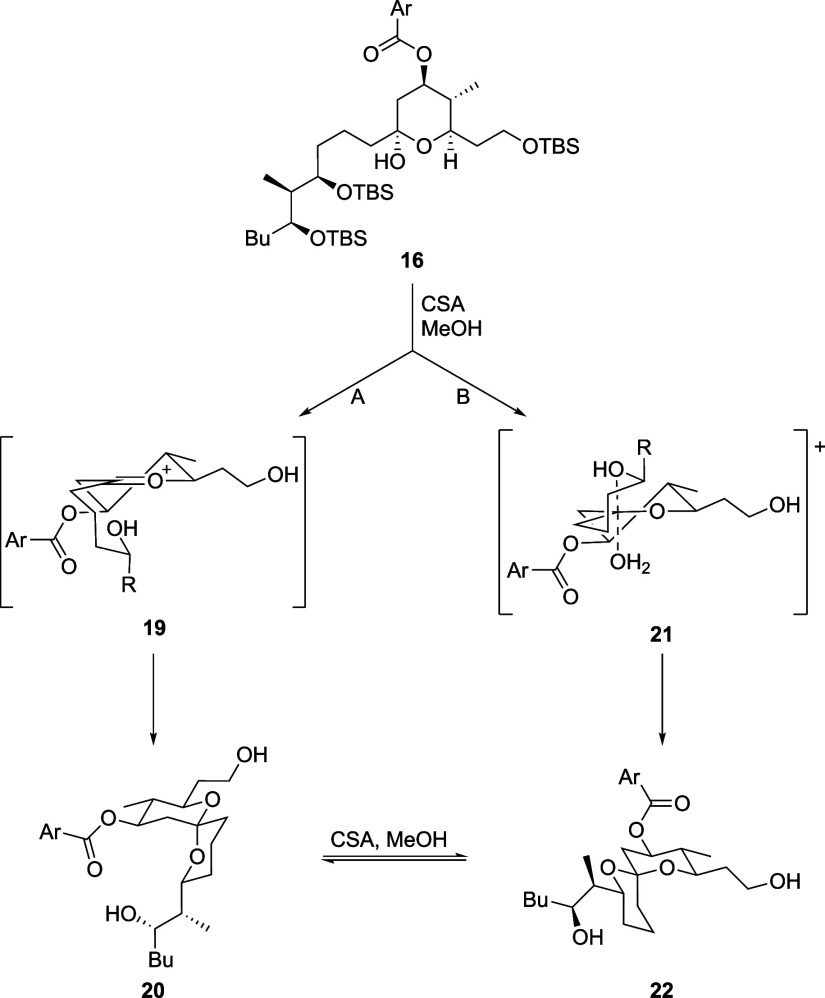
Mechanisms for Spirocycle Stereoisomer Formation (Ar = *p*-NO_2_Ph)

Our analysis of the NOEs in **17** showed strong agreement with those in the spirocycle subunit of the natural product but notable differences in the periphery. Fenical reported NOEs between the hydrogens on C35 and C25 and C27 for neaumycin B, which were not observed in **17**. An analysis of Smith’s data^3a^ also showed significant chemical shift differences between synthetic **1** and the natural product in the C35 region, indicating that this is a potential site of a stereochemical misassignment. We hypothesized that inverting the stereochemical configuration at C34 would resolve this issue through altering the preferred side chain conformation.

Testing this hypothesis (Scheme 6) required an *anti*-aldol reaction to establish the requisite stereochemical arrangement. Heathcock’s variant of the Evans aldol reaction proved suitable for this purpose.^16^ Boron enolate **23** was coupled with aldehyde **24** in the presence of Bu_2_BOTf. Cleaving the auxiliary from the resulting product with Me_3_Al and MeN(OMe)H·HCl provided Weinreb amide **25** in good yield as a separable 5:1 mixture of diastereomers. The sequence of BuLi addition, 1,3-*syn*-ketone reduction, and silyl ether formation yielded **26**. We noted that the intermediate boronate from the ketone reduction was unusually stable and discovered that treating with basic H_2_O_2_ followed by KHF_2_ is an effective method to cleave resilient cyclic boronates. Converting the terminal alkyne to a methyl ketone through gold-mediated hydration resulted in decomposition, leading us to pursue a hydroboration route. Yoshida’s copper-catalyzed borylation conditions^17^ with pinBBdan (pin = pinacol; dan = 1,8-diaminonaphthalene) selectively formed the branched isomer, which was oxidized in situ to deliver ketone **27**. This pathway provides a desirable method for hydrating acid sensitive alkynes. Enolsilane formation followed by a BF_3_·OEt_2_ promoted aldol reaction with **7** delivered **28** as a 6:1 mixture of diastereomers in 51% yield. Converting the newly generated alcohol to an acetate followed by PMB ether cleavage and acid mediated cyclization provided spiroketals **29** and **30** as a separable 2.5:1 mixture of interconvertible diastereomers. Acetylating the major diastereomer formed **31**.

**Scheme 6:**
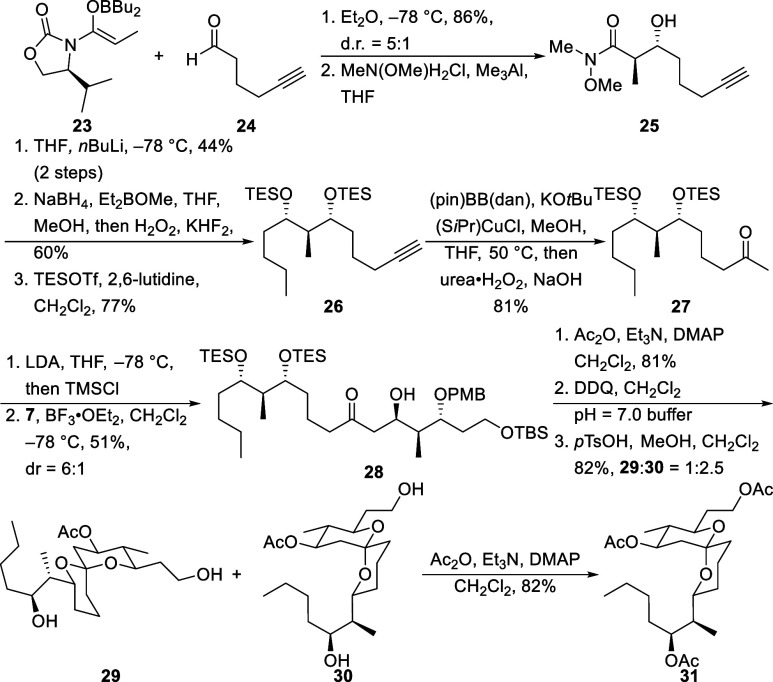
Synthesis of the C34 Epimer

A NOESY analysis of compound **31** revealed critical relationships that are observed in the natural product but not in **17**. Notably H35 shows NOEs to H25 and H27. While these hydrogens appear to be distal in the planar structure, their proximity is explained through superimposing the preferred conformation on a diamond lattice, as shown in Figure 3, where the light lines show implied bonds in the pattern.^18^ Placing H34 in an *anti*-orientation to H33 is desirable for minimizing *gauche*-interactions between the alkyl groups and the ring. This conformation shows the proximity between H35 and H25 and H27. A similar conformation for **17** places H35 distal to H25 and H27. Notably, the chemical shift for H35 in **31** is 0.28 ppm downfield relative to H35 in **17**, which is remarkably consistent with the respective chemical shifts in the natural product and Smith’s synthetic material (Δδ = 0.27 ppm). The downfield shift is also consistent with the spatial similarity to the hydrogens in the spirocycle that have a 1,3-diaxial relationship with an oxygen atom. This does not imply a rigid conformation in this region of the molecules, but rather a change in conformational bias that could contribute to the lack of biological activity for the initially proposed structure and illustrates the capacity of methyl groups to induce conformational changes.^19^ The Supporting Information contains a table that compares key NMR signals between **17**, **31**, natural neaumycin B and synthetic neaumycin B.

**Figure 3:**
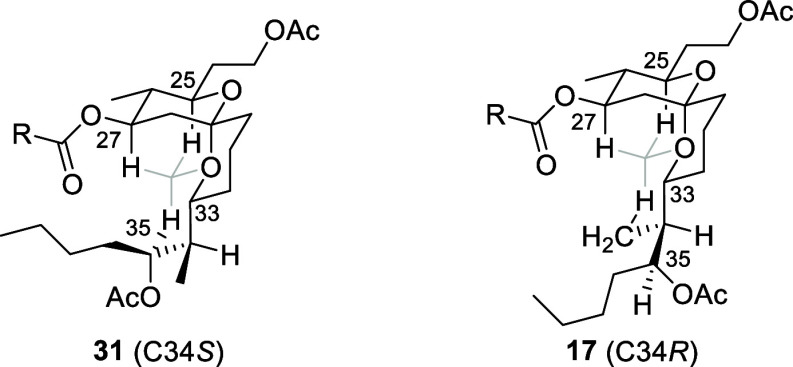
Conformational consequences of inverting C34.

We have described a convergent approach to the neaumycin B spiroketal in which a Mukaiyama aldol reaction serves to unite fragments while creating a product-relevant stereocenter. The spiroketal can form in either of two stereoisomeric forms, with both being singly anomeric. This is possible because spirocyclization creates both a centrally chiral stereocenter and an axilally chiral stereogenic unit. Spectroscopic analyses of the interconvertible spiroketals showed distinct chemical shift and NOE patterns that allow facile structural assignments. The lack of NOESY correlations in our initial structure that are present in the natural product led us to explore the possibility of a stereochemical misassignment in the side chain to C33 of the spirocycle. The spectroscopic data resulting from inverting the initial stereochemical assignment at C34 is much more consistent with the data from the natural product. While additional errors in the stereochemical assignment of the macrocycle will almost certainly be found, this work corrects one region of the molecule and serves as a reminder of the important role that synthesis can play in structural assignments for natural products.^20^

## Data Availability

The data underlying this study are available in the published article and the Supporting Information.

## Supporting Information Available

Substrate preparation and characterization data, including conformational analyses (PDF)
FAIR data, including the primary NMR FID files, for compounds **4**, **5**, **6**, **7**, **8**, **9**, **10**, **11**, **12**, **14**, **15**, **16**, **17**, **18**, **20**, **24**, **25**, **26**, **27**, **28**, **29**, **31**, **S1**, **S2**, **S3**, **S4**, **S5**, **S6**, and **S7**; see FID for Publication for further details (ZIP)
