# Supplementary material for: Rapid Synthesis of the Spiroketal Subunit of Neaumycin B: Stereochemical Aspects of Singly Anomeric Spiroketals and Proposal for a Stereocenter Reassignment
Source: Org Lett. 2024 Dec 9;26(50):10774–9. doi: 10.1021/acs.orglett.4c03751 (PMC11668044; doi:10.1021/acs.orglett.4c03751)
Supplement: Supplementary file 1 — ol4c03751_si_001.pdf [file ol4c03751_si_001.pdf]

Supporting Information

A Rapid Synthesis of the Spiroketal Subunit of Neaumycin B: Stereochemical Aspects of Singly Anomeric Spiroketals and Proposal for a Stereocenter Reassignment

Anna E. Healy, Marcus D. Van Engen, Nicholas A. Cinti, and Paul E. Floreancig*

Department of Chemistry
University of Pittsburgh
Pittsburgh, Pennsylvania 15260
florean@pitt.edu

Table of Contents

General Experimental	S3
Experimental Procedures and Characterization	S4
^{1}H and ^{13}C NMR Spectra	S19
Spectral Comparison Table	S52
References	S53

General Experimental

Proton (^{1}H) NMRs were recorded on Bruker Avance spectrometers at 300, 400, 500, and 600 MHz. Carbon (^{13}C) NMRs were recorded on Bruker Avance spectrometers at 100, 125, and 150 MHz. The chemical shifts are reported in parts per million (ppm) on the delta (δ) scale. The solvent peak was used as a reference value, for ^{1}H NMR: $CDCl_3$ = 7.26 ppm, CD_3CN = 1.94 ppm, for ^{13}C NMR: $CDCl_3$ = 77.2 ppm, CD_3CN = 1.3 ppm and 118.3 ppm. The coupling data are reported as follows: s = singlet; d = doublet; t = triplet; q = quartet; quin = quintet; m = multiplet. Structural assignments were made with additional information from gCOSY, gHSQC, and gHMBC experiments. High resolution mass spectra were collected on a ThermoFisher Q-Exactive Orbitrap instrument.

All distillations were performed under N_2 unless otherwise stated. Methylene chloride and acetonitrile were distilled from calcium hydride. Tetrahydrofuran was distilled over sodium/benzophenone. Methanol was distilled from ground calcium sulfate. Analytical TLC was performed on E. Merck pre-coated (25 mm) silica gel 60 F254 plates. Visualization was done under UV (254 nm) and by staining with anisaldehyde or $KMnO_4$ stain. Flash chromatography was done using SiliCycle SiliaFlash P60 40-63μm 60 Å silica gel. Reagent grade ethyl acetate, methanol, diethyl ether, acetonitrile, dichloromethane, and hexanes (commercial mixture) were purchased from Fisher Scientific and were used as-is for chromatography. All reactions were performed in flame-dried glassware under a positive pressure of argon with magnetic stirring unless noted otherwise.

Experimental Protocols

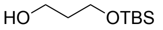

3-((*tert*-Butyldimethylsilyl)oxy)propan-1-ol (S1)

1,3-Propanediol (1.05 mL, 6.57 mmol) was added to a flame dried round bottom flask in CH_2Cl_2 (9.8 mL, 0.67 M). Et_3N (0.55 mL, 3.94 mmol) was then added and the solution was cooled to 0 °C. *Tert*-Butyldimethylsilyl chloride (495 mg, 3.29 mmol) added in one portion. The reaction mixture was warmed to rt and was stirred for 17 h. The reaction was quenched with saturated NH_4Cl. The organic layer was separated and the aqueous layer was extracted with CH_2Cl_2 (3x). The organic layer was washed with brine, dried with $MgSO_4$, and filtered then solvent was removed under reduced pressure. The crude mixture was then purified by flash column chromatography (5% EtOAc in hexanes to 15% EtOAc in hexanes), and **S1** was isolated as a clear oil (488 mg, 78%). The data match literature values.[1]

^{1}H NMR (500 MHz, $CDCl_3$): δ 3.83 (t, J = 5.6 Hz, 2H), 3.80 (t, J = 5.5 Hz, 2H), 2.61 (bs, 1H), 1.78 (p, J = 5.6 Hz, 2H), 0.87 (s, 9H), 0,74 (s, 6H)
^{13}C NMR (75 MHz, $CDCl_3$): δ 62.9, 62.4, 34.2, 25.9, 18.2, –5.5
HRMS (ESI): m/z calcd. for $C_9H_{23}O_2Si$ $[M+H]^+$ 191.1462, found 191.1463

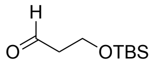

3-((*tert*-Butyldimethylsilyl)oxy)propanal (4)

A solution of DMSO (2.69 mL, 37.8 mmol) in CH_2Cl_2 (40 mL, 0.2 M) was cooled to –78 °C in a flame dried round bottom flask. Oxalyl chloride (1.49 mL, 17.3 mmol) was delivered dropwise and the solution was allowed to stir at –78° C for 20 min. **S1** (1.5 g, 7.9 mmol) was added to the solution and the reaction mixture was then allowed to stir at –78 °C for 1.5 h. Triethylamine (8.01 ml, 57.5 mmol) was then delivered to the reaction mixture dropwise at –78 °C, and the mixture was slowly warmed to rt. The reaction was then quenched with water, the organic layer was separated, and then the aqueous layer was extracted with CH_2Cl_2 (3x). The organic layer was then washed with saturated $NaHCO_3$, 1 M HCl, then brine and dried with $MgSO_4$ and filtered. The solvent was then removed under reduced pressure. **4** was obtained as a yellow oil (1.48 g, quantitative) and used without further purification.

^{1}H NMR (300 MHz, $CDCl_3$): δ 9.80 (t, J= 2.1 Hz, 1H), 3.99 (t, J= 6.0 Hz, 2H), 2.60 (td, J= 6.0, 2.1, 2H), 0.88 (s, 9H), 0.64 (s, 6H).
^{13}C NMR (75 MHz, $CDCl_3$): δ 202.1, 57.4, 46.6, 25.8, 18.2, –5.4.
These data match literature values.[1]

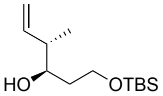

(3*R*,4*S*)-1-((*tert*-Butyldimethylsilyl)oxy)-4-methylhex-5-en-3-ol (5)

To a flame dried round bottom flask was added CuBr (59 mg, 0.41 mmol), NEt_3 (1.61 mL, 11.6 mmol), and ^{n}Bu$_4$Br (130 mg, 0.41 mmol) in CH_2Cl_2 (25 mL). This solution was cooled to 0° C. In a separate flask *trans*-crotyl chloride (0.81 mL, 8.3 mmol), Cl_3SiH (1.0 mL, 9.9 mmol), and CH_2Cl_2 (18 mL) were combined then added to the cooled solution dropwise. The resulting mixture was allowed to stir at 0 °C for 2 h. Ligand **A** (see text) (2.40 g, 8.27 mmol) and DBU (3.72 mL, 24.8 mmol) in CH_2Cl_2 (18 mL) were then added. The mixture was warmed to rt and was stirred for 1 h. The reaction was then cooled to –78 °C and **4** (1.48 g, 7.88 mmol) was added slowly. The reaction mixture was then stirred for 16 h at 0 °C. The solvent was removed under reduced pressure and the crude mixture was suspended in Et_2O (15 mL) and stirred for 20 min. The mixture was filtered then treated with ^{n}Bu$_4$NF (1 M in THF, 9.40 mL) at –

40 °C and stirred at this temperature for 1 h, then was treated with 1 M HCl (38 mL) and transferred to a separatory funnel. The organic layer was separated. The aqueous layer was extracted with Et_2O (3x). The organic layers were combined, washed with water then saturated $NaHCO_3$, dried with Mg_2SO_4, filtered through SiO_2, and the solvent was removed under reduced pressure. The crude mixture was then purified by flash column chromatography (5% EtOAc in hexanes to 10% EtOAc in hexanes), and **5** was isolated as a pale yellow oil (1.31 g, 68%).

^{1}H NMR (400 MHz, $CDCl_3$): δ 5.83 (m, 1H), 5.07 (m, 2H), 3.89 (m, 1H), 3.81 (m, 1H), 3.69 (m, 1H), 3.13 (d, *J* = 2.2 Hz, 1H), 2.24 (m, 1H), 1.63 (m, 2H), 1.04 (d, *J* = 6.9 Hz, 3H), 0.89 (s, 9H), 0.07 (s, 6H)
^{13}C NMR (100 MHz, $CDCl_3$): δ 140.9, 115.3, 75.2, 62.9, 44.1, 35.7, 26.0, 18.3, 15.9, –5.4
IR (ATR, neat): 3454, 2952, 2850, 1464, 1253, 1085, 834, 777 cm^{-1}
HRMS (ESI): *m/z* calcd. for $C_{13}H_{29}O_2Si$ $[M+H]^+$ 245.1932, found 245.1936
$[\alpha]^{25}_D$: –5.1 (*c* = 1.0 CH_2Cl_2)

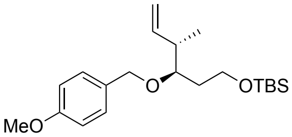

***tert*-Butyl(((3*R*,4*S*)-3-((4-methoxybenzyl)oxy)-4-methylhex-5-en-1-yl)oxy)dimethylsilane (6)**

5 (700 mg, 2.86 mmol) and $PMBOC(ONH)CCl_3$ (1.64 g, 5.81 mmol) were added to a flame dried round bottom flask in toluene (30 mL, 0.1 M). The resulting solution was cooled to 0 °C then $Sc(OTf)_3$ (70 mg, 0.15 mmol) was added. The resulting reaction mixture was warmed to rt and stirred for 16 h. The reaction was quenched with saturated NH_4Cl then diluted with EtOAc. The organic layer was separated and the aqueous layer was extracted with EtOAc (3x). The organic layers were combined, washed with brine, dried with Mg_2SO_4, filtered, and the solvent was removed under reduced pressure. The crude mixture was purified by flash column chromatography (1% Et_2O in hexanes to 3% Et_2O in hexanes), and **6** was isolated as a pale yellow oil and carried forward with inseparable PMB impurities which can be separated after the following step.

^{1}H NMR (400 MHz, $CDCl_3$): δ 7.27 (d, *J* = 8.6 Hz, 2H), 6.87 (d, *J* = 8.6 Hz, 2H), 5.81 (m, 1H), 5.04 (m, 2H), 4.52 (d, *J* = 11.0 Hz, 1H), 4.43 (d, *J* = 11.1 Hz, 1H), 3.80 (s, 3H), 3.68 (t, *J* = 6.4 Hz, 2H), 3.50 (m, 1H), 2.51 (m, 1H), 1.65 (q, *J* = 6.3 Hz, 2H), 1.03 (d, *J* = 6.9 Hz, 3H), 0.89 (s, 9H), 0.04 (s, 6H)
^{13}C NMR (500 MHz, $CDCl_3$): 159.4, 141.1, 131.6, 129.4, 114.9, 113.8, 79.4, 71.9, 60.2, 55.5, 40.8, 34.4, 29.9, 26.2, 18.6, 14.7, 1.33, –5.0
IR (ATR, neat): 3657, 2952, 1614, 1513, 1464, 1385, 1250, 1089, 953, 833, 775 cm^{-1}
HRMS (ESI): *m/z* calcd. for $C_{21}H_{37}O_3Si$ $[M+H]^+$ 365.2507, found 365.2515
$[\alpha]^{25}_D$: +1.8 (c = 1.0 CH_2Cl_2)

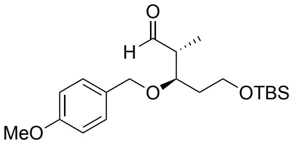

(2*R*,3*R*)-5-((*tert*-Butyldimethylsilyl)oxy)-3-((4-methoxybenzyl)oxy)-2-methylpentanal (7)

To a flame dried round bottom flask was added **6** (930 mg, 2.55 mmol) and a 4:1 mixture of THF:H_2O (10.2 mL:2.6 mL, 0.2 M). 4-Methylmorpholine oxide (448 mg, 3.83 mmol) was added to this solution followed by a 4% aqueous solution of OsO_4 (0.65 mL, 0.10 mmol). The resulting reaction mixture was allowed to stir at room temperature for 15 h. $NaIO_4$ (655 mg, 3.06 mmol) was then added to the reaction mixture which was stirred for 2 h. The aqueous

layer was then extracted with Et_2O (3x). The organic layers were combined, washed with brine, dried with $MgSO_4$, filtered and the solvent was removed under reduced pressure. The crude mixture was then purified by flash column chromatography (3% Et_2O in hexanes to 10% Et_2O in hexanes), and **7** was isolated as a clear oil (467 mg, 45% over 2 steps).

^{1}H NMR (500 MHz, $CDCl_3$): δ 9.71 (d, *J* = 2.0 Hz, 1H), 7.24 (d, *J* = 8.6 Hz, 2H), 6.87 (d, *J* = 8.6 Hz, 2H), 4.51 (d, *J* = 11.1 Hz, 1H), 4.45 (d, *J* = 11.1 Hz, 1H), 3.92 (ddd, *J* = 7.5, 5.7, 4.4 Hz, 1H), 3.80 (s, 3H), 3.73 (m, 2H), 2.70 (m, 1H), 1.79 (m, 1H), 1.71 (m, 1H), 1.10 (d, *J* = 7.0 Hz, 3H), 0.89 (s, 9H), 0.05 (d, *J* = 2.7 Hz, 6H)
^{13}C NMR (125 MHz, $CDCl_3$): δ 204.5, 159.4, 129.5, 114.0, 71.9, 66.0, 59.2, 55.4, 50.0, 34.84, 26.1, 18.4, 15.4, 10.0, –5.2
IR (ATR, neat): 3395, 2923, 2854, 1723, 1452, 1254, 1088, 1038, 809, 725 cm^{-1}
HRMS (ESI): *m/z* calcd. for $C_{20}H_{35}O_4Si$ $[M+H]^+$ 367.2300, found 367.2307
$[\alpha]^{25}_D$: –6.9 (*c* = 0.86 CH_2Cl_2)

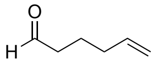

Hex-5-enal (8)
A solution of DMSO (11.5 mL, 161 mmol) in CH_2Cl_2 (100 mL) was cooled to –78 °C in a flame dried round bottom flask. Oxalyl chloride (7.0 mL, 82 mmol) was then added dropwise and the solution was stirred at –78 °C for 20 min. 5-Hexen-1-ol (5.0 g, 50 mmol) was then added to the solution and the reaction mixture was then stirred at –78 °C for 1.5 h. Triethylamine (40.4 ml, 290 mmol) was then delivered to the reaction mixture dropwise at –78 °C, and the mixture was slowly warmed to rt. The reaction was quenched with water, the organic layer was separated, and then the aqueous layer was extracted with CH_2Cl_2 (3x). The organic layers were then combined, washed with saturated $NaHCO_3$, 1 M HCl, then brine and dried with $MgSO_4$ and filtered. Compound **8** was used directly in the following reaction using the solvent from these extractions due to volatility of the aldehyde.

^{1}H NMR (300 MHz, $CDCl_3$): δ 9.73 (t, *J*= 1.6 Hz, 1H), 5.75 (ddt, *J*= 17.1, 10.2, 6.7 Hz, 1H), 5.00 (m, 2H), 2.41 (td, *J*= 7.3, 1.6 Hz, 2H), 2.06 (m, 2H), 1.70 (p, *J*= 7.3 Hz, 2H). These data match literature values.[2]

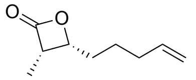

(3*S*,4*R*)-3-Methyl-4-(pent-4-en-1-yl)oxetan-2-one (9)
To a flame dried round bottom flask were added $LiClO_4$ (5.31 g, 49.9 mmol) and trimethylsilyl quinidine (0.5 M in Et_2O, 9.98 mL, 4.49 mmol) in Et_2O (83 mL, 0.6 M). This mixture was cooled to –78 °C and **8** (4.90 g, 49.9 mmol) in CH_2Cl_2 (150 mL, 0.2 M) was added dropwise followed by ^{i}Pr_2NEt (21.7 mL, 124.8 mmol). A solution of propionyl chloride (8.72 mL, 99.8 mmol) in CH_2Cl_2 (90 mL) was then added to the solution via syringe pump over the course of 3 h then the reaction mixture was stirred at –78 °C for 16 h. The reaction was diluted with Et_2O, filtered, and the solvent was removed under reduced pressure. The crude mixture was purified by flash column chromatography (10% Et_2O in hexanes to 25% Et_2O in hexanes), and **9** was isolated as a pale yellow oil (4.22 g, 56%).

^{1}H NMR (300 MHz, $CDCl_3$): δ 5.79 (m, 1H), 5.02 (m, 2H), 4.55 (ddd, *J* = 9.0, 6.4, 4.5 Hz, 1H), 3.74 (dq, *J* = 7.7, 6.5 Hz, 1H), 2.14 (m, 2H), 1.80-1.56 (m, 3H), 1.55-1.43 (m, 1H), 1.27 (d, *J* = 7.8 Hz, 3H)
^{13}C NMR (75 MHz, $CDCl_3$): δ 172.6, 137.8, 115.3, 95.5, 47.3, 33.2, 29.4, 24.6, 8.1

IR (ATR, neat): 2939, 1818, 1717, 1119, 912, 829 cm^{-1}
HRMS (ESI): *m/z* calcd. for $C_9H_{14}O_2$ $[M + H]^+$ 155.1067, found 155.1068
$[\alpha]^{25}_D$: +21.5 (c = 1.0 CH_2Cl_2)

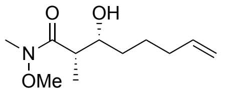

(2*S*,3*R*)-3-Hydroxy-*N*-methoxy-*N*,2-dimethyloct-7-enamide (S2)

A solution of *N,O*-dimethylhydroxylamine•HCl (1.27 g, 13.0 mmol) in CH_2Cl_2 (24 mL) was added to a flame dried round bottom flask and cooled to 0 °C. To this solution was added Me_2AlCl (0.9 M in heptanes, 14.4 mL, 13.0 mmol) dropwise over 30 min, then the resulting mixture was warmed to rt and stirred for 1 hour. The reaction was the cooled to –40 °C and a solution of **9** (1.0 g, 6.5 mmol) in CH_2Cl_2 (8 mL) was added dropwise then the resulting reaction mixture was stirred for 3 h. The reaction was quenched with saturated potassium sodium tartrate and the resulting biphasic mixture was warmed to rt and stirred until two distinct layers could be seen. The organic layer was separated, and the aqueous layer was extracted with CH_2Cl_2 (3x). The organic layers were combined, dried with Mg_2SO_4, filtered, and the solvent was removed under reduced pressure. The crude mixture was purified by flash column chromatography (10% EtOAc in hexanes to 40% EtOAc in hexanes), and **S2** was isolated as a clear oil (1.08 g, 78%).

^{1}**H NMR** (400 MHz, $CDCl_3$): δ 5.81 (ddt, J = 17.0, 10.3, 6.7 Hz, 1H), 4.99 (m, 2H), 3.86 (m, 1H), 3.70 (br s, 1H), 3.63 (s, 3H), 3.20 (s, 3H), 2.81 (m, 1H), 2.09 (m, 2H), 1.66-1.52 (m, 2H), 1.50-1.29 (m, 2H), 1.17 (d, J = 7.1 Hz, 3H)
^{13}**C NMR** (75 MHz, $CDCl_3$): δ 207.2, 138.7, 114.6, 71.4, 61.3, 33.7, 33.5, 25.4, 13.2, 10.2, 7.7
IR (ATR, neat): 3432, 2935, 1637, 1457, 1421, 1385, 991, 911, 620, 440
HRMS (ESI): *m/z* calcd. for $C_{11}H_{22}NO_3$ $[M+H]^+$ 216.1595, found 216.1594
$[\alpha]^{25}_D$: +16.7 (c = 1.0 CH_2Cl_2)

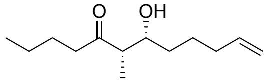

(6*S*,7*R*)-7-Hydroxy-6-methyldodec-11-en-5-one (10)

A solution of **S2** (1.18 g, 5.48 mmol) in THF (16 mL) was added to a flame dried round bottom flask and cooled to –78 °C. To this solution was added ^{n}BuLi (2.5 M in hexanes, 6.6 mL, 16.5 mmol) dropwise and the resulting reaction mixture was stirred for 1 h. The reaction was quenched with saturated NH_4Cl then warmed to rt. The aqueous layer was extracted with Et_2O (3x). The organic layers were combined, dried with Mg_2SO_4, filtered, and the solvent was removed under reduced pressure. The crude mixture was then purified by flash column chromatography (5% EtOAc in hexanes to 15% EtOAc in hexanes), and **10** was obtained as a clear oil (1.05 g, 90%).

^{1}**H NMR** (400 MHz, $CDCl_3$): δ 5.79 (ddt, J = 17.2, 10.1, 6.6 Hz, 1H), 4.98 (m, 2H), 3.89 (m, 1H), 2.78 (d, J = 2.8 Hz, 1H), 2.56 (dq, J = 7.2, 3.0 Hz, 1H), 2.48 (m, 2H), 2.07 (m, 2H), 1.64-1.45 (m, 4H), 1.39-1.22 (m, 4H), 1.12 (d, J = 7.3 Hz, 3H), 0.90 (t, J= 7.4 Hz, 3H)
^{13}**C NMR** (75 MHz, $CDCl_3$): δ 216.6, 138.7, 114.8, 71.0, 50.0, 41.8, 33.7, 33.6, 25.8, 25.4, 22.5, 14.0, 10.0
IR (ATR, neat): 3423, 2933, 2863, 1701, 1459, 1376, 992, 910, 551 cm^{-1}
HRMS (ESI): *m/z* calcd. for $C_{13}H_{25}O_2$ $[M+H]^+$ 213.1850, found 213.1852
$[\alpha]^{25}_D$: +1.7 (c = 1.0 CH_2Cl_2)

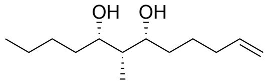

(5*S*,6*R*,7*R*)-6-Methyldodec-11-ene-5,7-diol (S3)

To a flame dried round bottom flask was added **10** (1.05 g, 4.95 mmol) in a 4:1 mixture of THF:MeOH (40 mL:10 mL, 0.1 M). This solution was cooled to –78 °C then Et_2BOMe (0.81 mL, 6.2 mmol) was added dropwise, and the resulting mixture was stirred for 1 h. $NaBH_4$ (281 mg, 7.42 mmol) was then added to the mixture in one portion and the reaction was stirred for 3 h. The reaction was quenched with a 30% aqueous solution of H_2O_2 and slowly warmed to rt. The resulting mixture was diluted with water and the aqueous layer was extracted with EtOAc (3x). The organic layers were combined, washed with saturated $NaHCO_3$, Na_2SO_3, and brine, then dried with Mg_2SO_4 and filtered. The solvent was removed under reduced pressure. The crude oil was purified by flash column chromatography (5% EtOAc in hexanes to 20% EtOAc in hexanes), and **S3** was isolated as a clear oil (955 mg, 90%).

^{1}H NMR (400 MHz, $CDCl_3$): δ 5.81 (ddt, *J* = 17.0, 10.2, 6.8 Hz, 1H), 5.00 (m, 2H), 3.85 (m, 2H), 2.56 (br s, 2H), 2.09 (q, *J* = 6.8 Hz, 2H), 1.60-1.48 (m, 3H), 1.46 – 1.30 (m, 5H), 1.31 – 1.23 (m, 1H), 0.91 (t, *J* = 6.8 Hz, 3H), 0.89 (d, *J* = 7.2 Hz, 3H)
^{13}C NMR (75 MHz, $CDCl_3$): δ 138.7, 114.8, 77.5, 77.3, 40.4, 35.2, 34.9, 33.8, 28.4, 25.5, 22.8, 14.2, 4.2
IR (ATR, neat): 3357, 2932, 2864, 1459, 961, 910 cm^{-1}
HRMS (ESI): *m/z* calcd. for $C_{13}H_{23}$ $[M–H_2O–OH]^+$ 179.1794, found 179.1793
$[\alpha]^{25}_D$: +4.4 (*c* = 1.0 CH_2Cl_2)

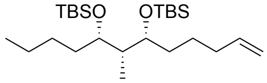

(5*S*,6*R*,7*R*)-5-Butyl-2,2,3,3,6,9,9,10,10-nonamethyl-7-(pent-4-en-1-yl)-4,8-dioxa-3,9-disilaundecane (11)

A solution of **S3** (60 mg, 0.28 mmol) in CH_2Cl_2 (2.8 mL, 0.1 M) was added to a flame dried round bottom flask and cooled to 0 °C. To this solution was added 2,6-lutidine (0.26 mL, 2.2 mmol) followed by dropwise addition of TBSOTf (0.25 mL, 1.1 mmol). The reaction mixture was warmed to rt and stirred for 2 h. The reaction was then quenched with saturated $NaHCO_3$. The organic layer was separated, and the aqueous layer was extracted with CH_2Cl_2 (3x). The organic layers were combined, washed with saturated NH_4Cl then brine, dried with Mg_2SO_4, filtered, and the solvent was removed under reduced pressure. The crude mixture was purified by flash column chromatography (100% hexanes to 2% EtOAc in hexanes), and **11** was isolated as a clear oil (116 mg, 94%).

^{1}H NMR (300 MHz, $CDCl_3$): δ 5.79 (ddt, *J* = 16.9, 10.3, 6.7 Hz, 1H), 4.97 (m, 2H), 3.67 (dq, *J* = 5.3, 2.0 Hz, 2H), 2.02 (q, *J* = 7.1 Hz, 2H), 1.59 (m, 1H), 1.52-1.42 (m, 4H), 1.41-1.22 (m, 6H), 0.87 (m, 21 H), 0.83 (d, *J* = 6.8 Hz, 3H), 0.02 (d, *J* = 2.9 Hz, 12H)
^{13}C NMR (75 MHz, $CDCl_3$): δ 139.0, 114.6, 72.95, 72.92, 40.6, 34.7, 34.3, 34.2, 27.1, 26.1, 24.1, 23.2, 18.3, 14.3, 9.8, –3.69, –3.71, –4.30
IR (ATR, neat): 2932, 1464, 1252, 1027, 832, 771, 668 cm^{-1}
HRMS (ESI): *m/z* calcd. for $C_{25}H_{55}O_2Si_2$ $[M+H]^+$ 443.3736, found 443.3742
$[\alpha]^{25}_D$: +1.0 (*c* = 1.0 CH_2Cl_2)

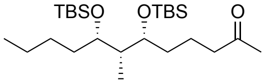

(6*R*,7*R*,8*S*)-6,8-Bis((*tert*-butyldimethylsilyl)oxy)-7-methyldodecan-2-one (12)

A solution of $PdCl_2$ (56 mg, 0.32 mmol) in a 10:1 mixture of DMF:H_2O (15.8 mL:1.58 mL) was added to a flame dried round bottom flask. CuCl (157 mg, 0.32 mmol) was added to the solution, and the reaction flask was backfilled with O_2 gas. The mixture was stirred for 1 h then **11** (700 mg, 1.58 mmol) was added and the resulting reaction mixture was stirred for 16 h. The reaction was then quenched with saturated NH_4Cl and filtered through Celite. The aqueous layer was extracted with EtOAc (3x). The organic layers were combined, washed with water, dried with Mg_2SO_4, filtered, and the solvent was removed under reduced pressure. The crude mixture was purified by flash column chromatography (100% hexanes to 5% EtOAc in hexanes), and **12** was isolated as a clear oil (486 mg, 67%).

^{1}H NMR (300 MHz, $CDCl_3$): δ 3.69 (m, 2H), 2.41 (td, *J* = 7.0, 2.9 Hz, 2H), 2.13 (s, 3H), 1.66-1.56 (m, 2H), 1.53-1.42 (m, 5H), 1.34-1.21 (m, 4H), 0.92-0.86 (m, 21H), 0.84 (d, *J* = 3.1 Hz, 3H), 0.03 (m, 12H)
^{13}C NMR (75 MHz, $CDCl_3$): δ 208.9, 72.9, 72.8, 44.2, 40.6, 34.8, 34.3, 29.9, 27.2, 26.1, 23.2, 19.2, 18.31, 18.29, 14.3, 9.7, –3.6, –3.8, –4.3
IR (ATR, neat): 2953, 2877, 1718, 1461, 1247, 1088, 1009, 832, 728 cm^{-1}
HRMS (ESI): *m/z* calcd. for $C_{25}H_{55}O_3Si_2$ $[M+H]^+$ 459.3690, found 459.3686
$[\alpha]^{25}_D$: +5.8 (*c* = 1.0 CH_2Cl_2)

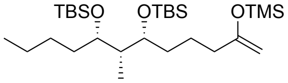

(8*R*,9*R*,10*S*)-10-Butyl-8-((*tert*-butyldimethylsilyl)oxy)2,2,9,12,12,13,13-heptamethyl-4-methylene-3,11-dioxa-2-12-disilatetradecane (13)

A solution of ^{i}Pr$_2$NH (0.10 mL, 0.74 mmol) in THF (1.2 mL) was added to a flame dried round bottom flask and cooled to 0 °C. ^{n}BuLi (2.5 M in hexanes, 0.27 mL, 0.68 mmol) was added to the solution dropwise, which was then stirred for 15 min before being cooled to –78 °C. **12** (280 mg, 0.61 mmol) was then added to the solution dropwise, using minimal THF to quantitate the transfer. The mixture was stirred for 10 min at –78 °C then TMSCl (0.09 mL, 0.68 mmol) was delivered dropwise. The reaction mixture was stirred for 10 min before being quenched with pH 7 buffer at –78 °C. The aqueous layer was then extracted with Et_2O (3x). The organic layers were combined, dried with $MgSO_4$, filtered and the solvent was removed under reduced pressure. The crude mixture was then placed under vacuum for 1 h, and **13** was obtained as a clear oil (321 mg, 99%) and was used without purification in the following step.

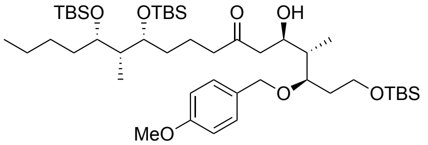

(5*S*,6*R*,7*R*,13*R*,14*S*,15*R*)-5-Butyl-7-((*tert*-butyldimethylsilyl)oxy)-13-hydroxy-15-((4-methoxybenzyl)oxy)2,2,3,3,6,14,19,19,20,20-decamethyl-4,18-dioxa-3,19-disilahenicosan-11-one (14)

A solution of **7** (37 mg, 0.10 mmol) in CH_2Cl_2 (1.0 mL) was added to a flame dried round bottom flask and cooled to –78 °C. BF_3•OEt_2 (12 µL, 0.10 mmol) was added to this solution dropwise, which was then stirred for 5 min. A solution of **13** (58mg, 0.11 mmol) in CH_2Cl_2 (0.3 mL) was delivered dropwise to the reaction mixture over the course of 45 min. The reaction was stirred for 1 h at –78 °C, then was quenched with saturated NH_4Cl and warmed to rt. The resulting biphasic mixture was poured into water, the organic layer was separated, and the aqueous layer was extracted with CH_2Cl_2 (3x). The organic layers were combined, dried with $MgSO_4$, filtered and

the solvent was removed under reduced pressure. The crude mixture was then purified by flash column chromatography (10% Et_2O in hexanes to 25% Et_2O in hexanes), and **14** was isolated as a clear oil (44 mg, 51%).

^{1}H NMR (500 MHz, $CDCl_3$): δ 7.24 (d, *J* = 8.6 Hz, 2H), 6.86 (d, *J* = 8.6 Hz, 2H), 4.53 (d, *J* = 10.8 Hz, 1H), 4.43 (m, 1H), 3.79 (s, 3H), 3.75-3.62 (m, 6H), 3.29 (s, 1H), 2.62 (dd, *J* = 16.4, 8.9 Hz, 1H), 2.46-2.32 (m, 3H), 1.84 (m, 2H), 1.66 (m, 1H), 1.63-1.57 (m, 8H), 1.33-1.24 (m, 5H), 0.97 (d, *J* = 7.0 Hz, 3H), 0.88 (m, 27H), 0.84 (d, *J* = 6.8 Hz, 3H), 0.03 (m, 18H)
^{13}C NMR (125 MHz, $CDCl_3$): δ 210.8, 159.4, 130.6, 129.7, 114.0, 79.9, 72.9, 72.8, 72.5, 67.5, 59.7, 55.4, 47.3, 44.2, 40.6, 40.4, 35.0, 34.7, 34.3, 29.8, 27.1, 26.1, 23.2, 18.9, 18.4, 18.30, 18.29, 14.3, 11.1, 9.7, –3.66, –3.74, –4.3, –5.2
IR (ATR, neat): 2929, 1710, 1513, 1462, 1249 1084, 1030, 833, 772 cm^{-1}
HRMS (ESI): *m/z* calcd. for $C_{45}H_{89}O_7Si_3$ $[M+H]^+$ 825.5930, found 825.5911
$[\alpha]^{25}_D$: +5.5 (*c* =1.0 CH_2Cl_2)

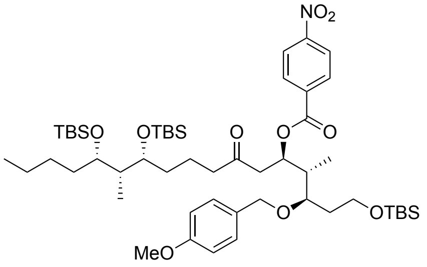

(7*R*,8*R*,9*R*,15*R*,16*R*,17*S*)-17-Butyl-15-((*tert*-butyldimethylsilyl)oxy)-7-((4-methoxybenzyl)oxy)2,2,3,3,8,16,19,19,20,20-decamethyl-11-oxo-4,18-dioxa-3,19-disilahenicosan-9-yl 4-nitrobenzoate (15)

A solution of **14** (39 mg, 0.05 mmol) and *p*-nitrobenzoic acid (16 mg, 0.09 mmol) in CH_2Cl_2 (1.5 mL) was added to a flame dried 2-dram vial and cooled to 0 °C. To this solution DCC (19 mg, 0.09 mmol) and DMAP (3.4 mg, 0.03 mmol) were added. The reaction mixture was warmed to rt and stirred for 3 h. The reaction was then filtered through Celite® and washed with saturated NH_4Cl and brine. The organic layer was separated, dried with $MgSO_4$, filtered, and the solvent was removed under reduced pressure. The crude mixture was purified by flash column chromatography (5% Et_2O in hexanes to 15% Et_2O in hexanes), and **15** was isolated as a clear oil (44 mg, 96%).

^{1}H NMR (400 MHz, $CDCl_3$): δ 8.20 (d, *J* = 8.9 Hz, 2H), 8.06 (d, *J* = 8.9 Hz, 2H), 7.19 (d, *J* = 8.6 Hz, 2H), 6.75 (d, *J* = 8.6 Hz, 2H), 5.81 (ddd, *J* = 6.9, 5.9, 3.6 Hz, 1H), 4.42 (d, *J* = 10.7 Hz, 1H), 4.33 (d, *J* = 10.7 Hz, 1H), 3.74 (s, 3H), 3.72 (m, 1H), 3.69-3.63 (m, 3H), 3.55 (m, 1H), 2.88 (dd, *J* = 16.3, 7.2 Hz, 1H), 2.73 (dd, *J* = 16.1, 5.7 Hz, 1H), 2.42 (m, 2H), 2.11 (m, 1H), 1.86-1.73 (m, 3H), 1.53-1.40 (m, 7H), 1.31-1.18 (m, 6H), 1.07 (d, *J* = 7.0 Hz, 3H), 0.86 (s, 27H), 0.81 (d, *J* = 7.0 Hz, 3H), 0.06 (m, 18H)
^{13}C NMR (100 MHz, $CDCl_3$): δ 207.2, 164.0, 159.3, 150.5, 136.0, 130.8, 129.8, 123.5, 113.8, 72.9, 72.76, 71.8, 71.7, 59.4, 55.3, 45.4, 43.7, 40.6, 39.8, 34.7, 34.3, 34.2, 27.1, 26.1, 23.2, 19.0, 18.4, 18.29, 18.27, 14.3, 10.8, 9.7, 1.2, –3.66, –3.74, –4.29, –4.32, –5.20, –5.21
IR (ATR, neat) 2928, 2857, 1722, 1523, 1344, 1250, 1089, 1029, 832, 773 cm^{-1}
HRMS (ESI): *m/z* calcd. for $C_{52}H_{90}NO_{10}Si_3$ $[M–H]^-$ 972.5867, found 972.5899
$[\alpha]^{25}_D$: –4.7 (*c* = 0.30 CH_2Cl_2)

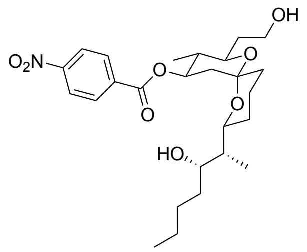

(2*R*,3*R*,4*R*,6*S*,8*R*)-2-(2-Hydroxyethyl)-8-((2*R*,3*S*)-3-hydroxyheptan-2-yl)-3-methyl-1,7-dioxaspiro[5.5]undecane-4-yl 4-nitrobenzoate (20)

A solution of **15** (20 mg, 0.02 mmol) in a 10:1 mixture of CH_2Cl_2 and pH 7 buffer (0.9 mL:0.09 mL) was added to a 2-dram vial and cooled to 0 °C. DDQ (7.3 mg, 0.03 mmol) was added to the solution, which was warmed to rt and stirred for 1.5 h. The reaction was quenched with $NaHCO_3$ and the organic layer was separated. The aqueous layer was extracted with EtOAc (3x). The organic layers were combined, washed with brine, dried with $MgSO_4$, filtered, and solvent was removed under reduced pressure to obtain **16** which was used directly in the following reaction. The crude mixture was dissolved in MeOH (1 mL) and CSA (1 mg, 4 μmol) was added. The reaction was stirred for 10 min. The solvent was removed under reduced pressure and the crude mixture was purified by flash column chromatography (30% Et_2O in hexanes to 40% hexanes in Et_2O), and the product was obtained as a 2:1 mixture of epimers (6 mg, 58%). The mixture was able to be purified by preparatory TLC (20% hexanes in MTBE) to deliver **S4** as a clear oil (3 mg, 29%).

^{1}H NMR (500 MHz, C_6D_6): δ 7.78 (d, *J* = 8.9 Hz, 2H), 7.71 (d, *J* = 8.9 Hz, 2H), 5.18 (td, *J* = 16.7, 4.4 Hz, 1H), 4.01 (m, 2H), 3.81-3.73 (m, 3H), 2.81 (dd, *J* = 12.7, 4.4 Hz, 1H), 2.26 (m, 1H), 1.76-1.51 (m, 7H), 1.47-1.41 (m, 3H), 1.40-1.32 (m, 6H), 0.94 (t, *J* = 7.2 Hz, 3H), 1.25-1.17 (m, 3H), 1.12 (d, *J* = 7.1 Hz, 3H), 0.78 (d, *J* = 6.5 Hz, 3H)
^{13}C NMR (500 MHz, C_6D_6): δ 164.3, 150.8, 135.5, 130.5, 123.6, 99.2, 78.1, 74.6, 74.6, 60.6, 60.6, 42.5, 40.8, 35.8, 35.5, 35.4, 34.6, 29.0, 28.2, 23.2, 20.1, 14.4, 13.1, 7.3
IR (ATR, neat): 3493, 2930, 2857, 1514, 1464, 1250, 1085, 1030, 832, 773 cm^{-1}
HRMS (ESI): *m/z* calcd. for $C_{26}H_{40}NO_8$ $[M+H]^+$ 494.2749, found 494.2752
$[\alpha]^{25}_D$: +1.3 (*c* = 0.30 CH_2Cl_2)

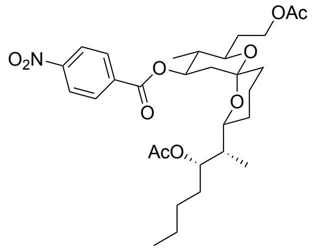

(2*R*,3*R*,4*R*,6*S*,8*R*)-2-(Acetoxyethyl)-8-((2*S*,3*S*)-3-acetoxyheptan-2-yl)-3-methyl-1,7-dioxaspiro[5.5]undecane-4-yl 4-nitrobenzoate (17)

A solution of **20** (1.5 mg, 3x10^{-3} mmol) in CH_2Cl_2 (0.3 mL, 0.01 M) was added to a flame dried 2-dram vial. NEt_3 (7 μL, 0.05 mmol) and DMAP (0.05 mg, 4x10^{-4} mmol) were then added to the resulting solution before delivering acetic anhydride (3 μL, 0.03 mmol) dropwise. The reaction mixture was stirred for 3 h. The solvent was removed under reduced pressure and the crude reaction mixture was purified by flash column chromatography (5% Et_2O in hexanes to 20% Et_2O in hexanes) and **17** was obtained as a clear oil (1.7 mg, 95%).

^{1}H NMR (500 MHz, C_6D_6): δ 7.75 (d, *J* = 9.0 Hz, 2H), 7.71 (d, *J* = 9.0 Hz, 2H), 5.33 (ddd, *J* = 8.5, 4.6, 4.0 Hz, 1H), 5.10 (td, *J* = 16.2, 4.5 Hz, 1H), 4.43 (m, 2H), 4.12 (ddd, *J* = 10.2, 9.2, 2.5 Hz, 1H), 3.63 (ddd, *J* = 11.2, 7.0, 1.9 Hz, 1H), 2.83 (dd, *J* = 12.7, 4.4 Hz, 1H), 2.04 (m, 1H), 2.00 (s, 3H), 1.77 (s, 3H), 1.76-1.71 (m, 3H), 1.69-1.62 (m, 3H), 1.56 (dd, *J* = 13.6, 5.3 Hz, 1H), 1.52-1.44 (m, 3H), 1.34-1.28 (m, 5H), 1.26 (d, *J* = 6.9 Hz, 3H), 1.19-1.15 (m, 2H), 0.83 (d, *J* = 6.5 Hz, 3H)

^{13}C NMR (125 MHz, C_6D_6): δ 170.3, 170.2, 164.3, 150.7, 135.5, 130.4, 123.6, 98.7, 75.2, 74.3, 73.9, 71.4, 61.3, 43.1, 40.7, 35.5, 34.4, 32.7, 32.4, 28.9, 28.6, 23.0, 20.9, 20.6, 20.2, 14.3, 13.1, 10.3
IR (ATR, neat): 2958, 1729, 1531, 1374, 1269, 1248, 1102, 1020, 721 cm^{-1}
HRMS (ESI): *m/z* calcd. for $C_{30}H_{44}NO_{10}$ [M+H]$^+$ 578.2960, found 578.2955

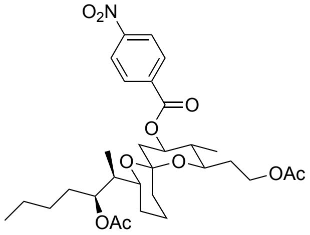

(2*R*,3*R*,4*R*,6*R*,8*R*)-2-(Acetoxyethyl)-8-((2*S*,3*S*)-3-acetoxyheptan-2-yl)-3-methyl-1,7-dioxaspiro[5.5]undecane-4-yl 4-nitrobenzoate (18) A solution of *epi*-**20** (1.5 mg, 3x10^{-3} mmol) in CH_2Cl_2 (0.3 mL, 0.01 M) was added to a flame dried 2-dram vial. NEt_3 (7 µL, 0.05 mmol) and DMAP (0.05 mg, 4x10^{-4} mmol) were then added to the resulting solution before delivering acetic anhydride (3 µL, 0.03 mmol) dropwise. The reaction mixture was stirred for 3 h. The solvent was removed under reduced pressure and the crude reaction mixture was purified by flash column chromatography (5% Et_2O in hexanes to 20% Et_2O in hexanes) and **18** was obtained as a clear oil (1.5 mg, 87%).

^{1}H NMR (500 MHz, C_6D_6): δ 7.76 (d, *J* = 9.0 Hz, 2H), 7.72 (d, *J* = 9.0 Hz, 2H), 5.35 (ddd, *J* = 7.0, 5.6, 5.4 Hz, 1H), 4.92 (td, *J* = 11.0, 4.9 Hz, 1H), 4.33 (dd, *J* = 7.6, 5.8 Hz, 2H), 4.12 (ddd, *J* = 11.8, 4.7, 1.9 Hz, 1H), 2.95 (td, *J* = 9.9, 2.4 Hz, 1H), 2.16 (dd, *J* = 12.2, 4.9 Hz, 1H), 1.86 (s, 3H), 1.74 (s, 3H), 1.72-1.64 (m, 4H), 1.64-1.56 (m, 2H), 1.54-1.43 (m, 4H), 1.33-1.26 (m, 7H), 1.24-1.19 (m, 2H), 1.10 (d, *J* = 7.1 Hz, 3H), 1.03 (dd, *J* = 13.5, 4.2 Hz, 1H), 0.65 (d, *J* = 6.6 Hz, 3H).
^{13}C NMR (125 MHz, C_6D_6): δ 170.1, 164.2, 150.8, 135.4, 130.6, 123.5, 97.6, 74.9, 74.7, 72.1, 71.6, 61.3, 42.6, 41.7, 41.3, 33.0, 32.1, 30.2, 29.5, 28.5, 28.0, 23.0, 20.9, 20.5, 18.8, 14.2, 12.8, 10.2
HRMS (ESI): *m/z* calcd. for $C_{30}H_{44}NO_{10}$ [M+H]$^+$ 578.2960, found 578.2955

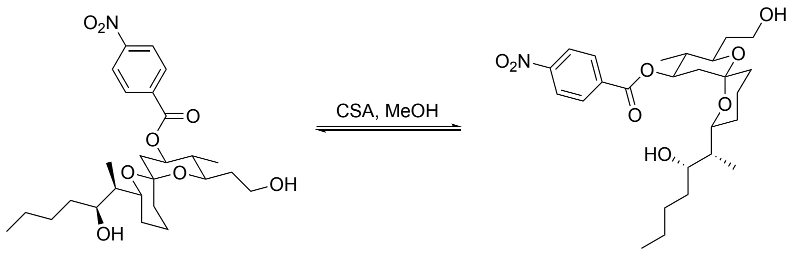

22 20

Minor spirocycle **22** (1.00 mg, 2.00 µM) was dissolved in MeOH (0.05 mL) and a 0.01M solution of CSA in MeOH was added (0.04. mL, 0.40 µM). The resulting solution was stirred. After 5 h a 1:1 mixture with **20** was present, and after 24 h a 1:2 mixture of **22**:**20** was observed.

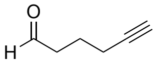

Hex-5-ynal (24)
To a solution of DMSO (11.7 mL, 165 mmol) in DCM (200 mL) at −78 °C was added oxalyl chloride (7.31 mL, 83.5 mmol). The resulting mixture was stirred for 20 min then 5-hexynol (5.00 g, 50.9 mmol) was added and the solution was stirred for an additional 20 min. After this time NEt_3 (41.0 mL, 296 mmol) was added dropwise. After stirring for 10 min the solution was warmed to 0 °C then rt. Upon completion, the reaction was quenched with water and extracted with DCM. The combined organic layers were washed consecutively with sat. bicarb, 1M HCl,

and brine then dried over $MgSO_4$ and concentrated. The resulting oil was used without further purification.

^{1}H NMR (300 MHz, $CDCl_3$) δ 9.83 (t, *J* = 1.3 Hz, 1H), 2.63 (td, *J* = 7.3, 1.3 Hz, 2H), 2.30 (td, *J* = 6.9, 2.7 Hz, 2H), 2.00 (t, *J* = 2.7 Hz, 1H), 1.88 (q, *J* = 7.0 Hz, 2H). The spectroscopic data is consistent with literature reported data.[3]

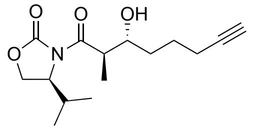

(*S*)-3-((2*R*,3*R*)-3-Hydroxy-2-methyloct-7-ynoyl)-4-isopropyloxazolidin-2-one (S4)

(*S*)-4-Isopropyl-3-propionyloxazolidin-2-one (5.00 g, 27.0 mmol) was dissolved in diethyl ether (135 mL) and cooled to 0 °C. $^nBu_2BOTf$ (1M in CH_2Cl_2, 54.0 mL, 54.0 mmol) was added and the reaction was stirred for 5 min, then $^iPr_2NEt$ (5.41 mL, 31.0 mmol) was added. The resulting solution was stirred for 1 h then was cooled to 78 °C. Aldehyde **24** (3.40 g, 35.4 mmol) was added dropwise over 10 min and the reaction was stirred for 2 h. Tartaric acid (20 g, 0.130 mol) was added, then the solution was warmed to rt and stirred for an additional 2 h. Water was added and the layers were separated. The aqueous layer was extracted with Et_2O. The combined organic layers were then stirred with a 3:1 mixture of MeOH and 30% H_2O_2 at 0 °C for 30 min. The resulting light-yellow solution was diluted with water and extracted with Et_2O then washed with sat. $NaHCO_3$ and brine then dried over $MgSO_4$ and concentrated. The resulting residue was purified via flash chromatography (40% Et_2O/hexanes) to afford the desired product as an inseparable 5:1 mixture of diastereomers (6.50 g, 85.6%, >99% ee as confirmed by Mosher ester).

^{1}H NMR (400 MHz, $CDCl_3$): δ 4.47- 4.40 (m, 1H), 4.31-4.24 (m, 1H), 4.21 (dd, *J* = 9.13, 2.93 Hz, 1H), 3.90 (quin, *J* = 6.9 Hz, 1H), 3.72 – 3.64 (m, 1H), 2.77 – 2.51 (br, 1H), 2.45 – 2.30 (m, 1H), 2.26 – 2.19 (m, 2H), 1.93 (t, *J* = 2.5 Hz, 1H), 1.84 – 1.69 (m, 2H), 1.66- 1.47 (m, 2H), 1.86 (d, *J* = 6.9 Hz, 3H), 0.913 (d, *J* = 7.0 Hz, 2H), 0.883 (d, *J* = 6.9 Hz, 3H)
^{13}C NMR (400 MHz, $CDCl_3$): δ 176.7, 154.3, 84.1, 74.3, 68.7, 58.6, 43.2, 33.3, 28.5, 24.4, 18.2, 18.0, 14.7, 14.5
IR (ATR, neat): 3493, 3293, 2956, 1776, 1699, 1374, 1203 cm^{-1}
HRMS (ESI): *m/z* calcd. for $C_{15}H_{23}NO_4$ $[M+H]^+$ 282.1697, found 282.1710
$[\alpha]^{25}_D$: +47.6 (*c* =1.0 CH_2Cl_2)

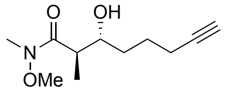

(2*R*,3*R*)-3-Hydroxy-*N*-methoxy-*N*,2-dimethyloct-7-ynamide (25)

N,O-Dimethyl hydroxylamine hydrochloride (3.40 g, 35.2 mmol) was dissolved in THF (26.7 mL) and cooled to 0 °C. $AlMe_3$ (17.6 mL of a 2M solution in hexanes, 35.2 mmol) was carefully added dropwise and the resulting solution was allowed to stir for 1 h at rt. A solution of **S4** (3.00 g, 10.7 mmol) in THF (4 mL) was then added dropwise and stirred overnight. The reaction was quenched by the careful addition of 1M HCl, filtered through Celite, and extracted with Et_2O. The combined organic layers were washed with brine, dried over $MgSO_4$ and concentrated. The crude material was then purified by flash chromatography (100% Et_2O) to afford pure minor diastereomer (0.61 g, 2.90 mmol) and a 2:1 inseparable mixture of the major diastereomer an oxazolidinone derived byproduct which was used without further purification (1.52 g).

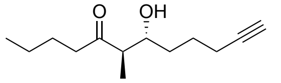

(6*R*,7*R*)-7-hydroxy-6-methyldodec-11-yn-5-one (S5)

Weinreb amide **S4** (1.52 g of a 2:1 mixture of amide and oxazolinone) was dissolved in THF (13.5 mL) and cooled to −78 °C. ^{n}BuLi (2.5 M in hexanes, 11.0 mL, 28.0 mmol) was then added dropwise and the resulting bright yellow solution was stirred for 4 h. The reaction was quenched with sat. aqueous NH_4Cl and warmed to rt. The layers were separated, and the aqueous layer was extracted with Et_2O. The combined organic layers were washed with brine, dried over $MgSO_4$ and concentrated. The resulting residue was purified by flash chromatography (10% EtOAc in hexanes) to afford the desired product as a clear oil (0.810 g, 44% of a single diastereomer over two steps).

^{1}H NMR (400 MHz, $CDCl_3$): δ 3.74 – 3.66 (m, 1H), 2.60 (d, *J* = 6.47 Hz, 1H) 2.63 (quin, *J* = 7.1 Hz, 1H), 2.58 – 2.38 (m, 2H), 2.23 (td, *J* = 6.6, 2.42 Hz, 2H), 1.95 (t, *J* = 2.4 Hz, 1H), 1.81 – 1.70 (m, 1H), 1.69 – 1.42 (m, 6H), 1.31 (sextet, *J* = 7.4 Hz, 1H), 1.14 (d, *J* = 7.3 Hz, 3H), 0.905 (t, *J* = 7.3 Hz, 3H)
^{13}C NMR (400 MHz, $CDCl_3$): δ 216.8, 84.4, 73.6, 68.7, 51.4, 42.6, 33.8, 25.8, 24.5, 22.4, 18.5, 14.4, 14.0
IR (ATR Neat): 3310, 2934, 1702, 1458, 530 cm^{-1}
HRMS (ESI): *m/z* calcd. for $C_{13}H_{23}O_2$ $[M+H]^+$ 211.1693, found 211.1698
$[\alpha]^{25}_D$: +1.9 (*c* =1.0 CH_2Cl_2)

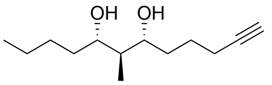

(5*S*,6*S*,7*R*)-6-Methyldodec-11-yne-5,7-diol (S6)

To a flame dried round bottom flask was added hydroxy ketone **S5** (0.700 g, 3.30 mmol) in a 4:1 mixture of THF and MeOH (33 mL). To this solution was added Et_2BOMe (1 M in THF, 6.60 mL, 6.60 mmol) and the resulting solution was stirred for 30 min. The mixture was cooled to −78 °C and $NaBH_4$ (0.250 g, 6.60 mmol) was added in one portion. This was then stirred for 4 h and quenched with 30% aq. H_2O_2 and KHF_2 and allowed to warm to rt. The solution was diluted with water and extracted with Et_2O. The combined organic layers were washed with $Na_2S_2O_3$ and brine, then dried over $MgSO_4$ and concentrated. The crude oil was purified via flash chromatography (30% Et_2O in hexanes) to give the product as a viscous yellow oil (0.420 g, 60%).

^{1}H NMR (300 MHz, $CDCl_3$): δ 3.72-3.58 (m, 2H), 2.98 (br, 2H), 2.57 – 2.20 (m, 2H), 1.95 (t, *J* = 2.6 Hz, 1H), 1.84 – 1.70 (m, 2H), 1.69 – 1.49 (m, 5H), 1.48 – 1.29 (m, 4H), 0.91 (t, *J* = 7.1 Hz, 3H), 0.81 (d, *J* = 6.9 Hz, 3H)
^{13}C NMR (400 MHz, $CDCl_3$): δ 84.5, 75.7, 68.5, 43.8, 34.8, 33.8, 29.7, 27.2, 23.9, 22.8, 18.3, 14.1, 13.1
IR (ATR Neat): 3229, 2980, 1383, 1151, 955 cm^{-1}
HRMS (ESI): *m/z* calcd. for $C_{13}H_{25}O_2$ $[M+H]^+$ 213.1849, found 213.1854
$[\alpha]^{25}_D$: +1.5 (*c* =1.0 CH_2Cl_2)

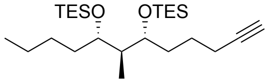

(5*S*,6*S*,7*R*)-5-Butyl-3,3,9,9-tetraethyl-6-methyl-7-(pent-4-yn-1-yl)-4,8-dioxa-3,9-disilaundecane (26)

Diol **S6** (0.250 g, 1.18 mmol) was dissolved in CH_2Cl_2 (2.35 mL) and cooled to 0 °C. 2,6-Lutidine (1.10 mL, 9.42 mmol) was added followed by TESOTf (1.10 mL, 4.70 mmol). The resulting solution was stirred for 1 h then was quenched with sat. $NaHCO_3$ and

extracted with DCM. The organic layer was washed with brine, dried over $MgSO_4$, and concentrated. The resulting residue was purified via flash chromatography (0-5% Et_2O in hexanes) to afford the desired product as a yellow oil (0.419 g, 77%).

^{1}H NMR (300 MHz, $CDCl_3$): δ 3.71 (q, *J* = 5.1 Hz, 2H), 2.23 – 2.09 (m, 2H), 1.95 – 1.88 (m, 1H), 1.74 (sextet, *J* = 6.7 Hz, 1H), 1.68 – 1.57 (m, 2H), 1.56 – 1.40 (m, 3H), 1.35– 1.15 (m, 5H), 0.939 (t, *J* = 8.0 Hz, 21 H), 0.746 (d, *J* = 7.1 Hz, 3H), 0.567 (q, *J* = 7.9 Hz, 12 H)
^{13}C NMR (400 MHz, $CDCl_3$): δ 84.7, 73.2, 72.5, 43.6, 32.7, 31.8, 26.8, 23.9, 23.1, 18.8, 14.2, 9.6, 6.9, 5.3
IR (ATR Neat): 2918, 1458, 1239, 1005, 725, 629 cm^{-1}
HRMS (ESI): *m/z* calcd. for $C_{25}H_{53}O_2Si_2$ $[M+H]^+$ 441.35786, found 441.35889
$[\alpha]^{25}_D$: +3.7 (*c* =1.0 CH_2Cl_2)

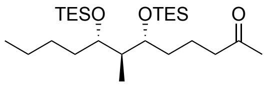

(6*R*,7*S*,8*S*)-7-Methyl-6,8-bis((triethylsilyl)oxy)dodecan-2-one (27)
To a solution of (1,3-bis(2,6-diisopropylphenyl)imidazolidin-2-yl)copper(II) chloride (10.0 mg, 19.7 μmol) in THF (1 mL) was added KO^{t}Bu (3.00 mg, 28.1 μmol) and MeOH (0.34 mL, 0.844 mmol). The resulting mixture was cooled to 0 °C and B(pin)-B(dan) (0.107 g, 0.422 mmol) was added. The bright yellow solution was warmed to rt. Once the solution was a pale yellow color alkyne **26** (0.124 g, 0.281 mmol) was then added and the resulting solution was heated to 50 °C in a sand bath and stirred for 4 h. The reaction mixture was cooled to rt then concentrated. The crude residue was filtered through a silica plug eluting with 5% Et_2O in hexanes, then was concentrated and redissolved in THF (1.4 mL). A 0.50 M solution of NaOH in MeOH (0.843 mL) and ^{t}BuOOH (5.5 M in decane, 0.112 mL, 0.61 mmol) were added and the resulting solution was stirred for 2.5 h. The reaction was quenched with aq. $Na_2S_2O_3$ and extracted with EtOAc, then was filtered through Celite. The combined organic layers were washed with $Na_2S_2O_3$ and dried over $MgSO_4$ then were concentrated. The resulting crude residue was purified via flash chromatography (10% Et_2O in hexanes) to give methyl ketone **27** as a single regioisomer (0.104 g, 81%).

^{1}H NMR (400 MHz, $CDCl_3$): δ 3.73 – 3.68 (m, 1H), 3.67 – 3.61 (m, 1H), 2.40 (t, *J* = 7.5 Hz, 2H), 2.12 (s, 3H), 1.73 – 1.65 (m, 1H), 1.64 – 1.57 (m, 1H), 1.55 – 1.46 (m, 1H), 1.33 – 1.26 (m, 5H), 1.25 – 1.13 (m, 3H), 0.88 (t, *J* = 7.9 Hz, 18H), 0.83 (t, *J* = 6.9, 3H), 0.68 (d, *J* = 7.0 Hz, 3H), 0.58 (q, *J* = 7.9 Hz, 6H), 0.57 (q, *J* = 7.9 Hz, 6H)
^{13}C NMR (500 MHz, $CDCl_3$): δ 209.0, 73.3, 72.9, 68.1, 44.3, 43.6, 32.7, 32.3, 29.8, 26.9, 25.6, 23.2, 19.6, 14.2, 9.8, 7.0, 5.2,
IR (ATR, neat): 2954, 1719, 1459, 1238, 1075, 1005, 723 cm^{-1}
HRMS (ESI) *m/z* calcd. for $C_{25}H_{55}O_3Si_2$ $[M+H]^+$ 459.3684, found 459.3694
$[\alpha]^{25}_D$: +2.9 (*c* =1.0 CH_2Cl_2)

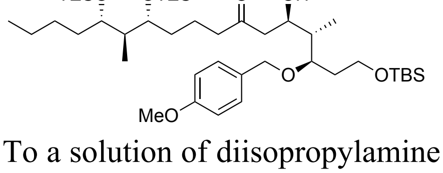

(7*R*,8*S*,9*R*,15*R*,16*S*,17*S*)-17-Butyl-19,19-diethyl-9-hydroxy-7-((4-methoxybenzyl)oxy)-2,2,3,3,8,16-hexamethyl-15-((triethylsilyl)oxy)-4,18-dioxa-3,19-disilahenicosan-11-one (28)
To a solution of diisopropylamine (0.048 mL, 0.34 mmol) in THF (1.1 mL) at −78 °C was added ^{n}BuLi (2.5 M in hexanes, 0.13 mL, 0.33 mmol) dropwise. The resulting solution was stirred for 5

min, then TMSCl (0.054 mL, 0.42 mmol) was added. After stirring for 10 min a solution of methyl ketone **27** (0.094 g, 0.21 mmol) in THF (0.5 mL) was added dropwise, then the mixture was stirred for an additional 15 min. The mixture was quenched with pH = 7 buffer and warmed to rt. The layers were separated, and the aqueous layer was extracted with Et_2O and dried over $MgSO_4$. The solvent was removed under reduced pressure and the crude oil was used without further purification. Aldehyde **7** (0.050 g, 0.15 mmol) was dissolved in DCM (1.5 mL) and cooled to −78 °C. BF_3•OEt (20.0 μL, 0.15 mmol) was added and stirred for 5 min. The crude silyl enol ether was dissolved in DCM (0.5 mL) and added dropwise. The resulting mixture was stirred for 30 min then the reaction was quenched with saturated NH_4Cl and warmed to rt. The aqueous layer was extracted with DCM, washed with brine, and dried over $MgSO_4$. The solution was concentrated, and the crude material was purified via flash chromatography (10% Et_2O/hexanes). The pure material was isolated as a clear and colorless oil (0.065 g, 51%).

^{1}H NMR (500 MHz, $CDCl_3$): δ 7.24 (d, *J* = 8.5 Hz, 2H), 6.86 (d, *J* = 8.6 Hz, 2H), 4.53 (d, *J* = 11.0 Hz, 1H), 4.43 (d, *J* = 10.7 Hz, 1H), 4.41- 4.36 (m, 1H), 3.79 (s, 3H), 3.74 – 3.68 (m, 4H), 3.67 – 3.61 (m, 1H), 3.34 (br s, 1H), 2.61 (dd, *J* = 16.4, 8.8 Hz, 1H), 2.44 (m, 3H), 1.87 – 1.81 (m, 1H), 1.76 (q, *J* = 1.8 Hz, 1H), 1.71 – 1.63 (m, 2H), 1.39 – 1.33 (m, 5H), 1.32 – 1.24 (m, 4H), 0.95 (overlapping t and m, *J* = 7.9 Hz, 21 H), 0.90 (s, 12H), 0.76 (d, *J* = 7.0 Hz, 3H), 0.58 (q, *J* = 7.9 Hz, 6H), 0.58 (q, *J* = 7.9 Hz, 6H), 0.05 (s, 6H)
^{13}C NMR (400 MHz, $CDCl_3$): δ 210.6, 159.5, 130.9, 129.4, 114.3, 79.7, 73.2, 72.8, 72.4, 67.3, 59.6, 55.3, 47.2, 44.2, 43.6, 40.2, 34.8, 32.6, 32.3, 26.9, 26.0, 23.1, 19.1, 18.3, 14.1, 10.9, 9.8, 7.0, 5.2, -5.3
IR (ATR, neat): 3504, 2953, 1711, 1513, 1461, 1247, 1079, 1005, 834, 725 cm^{-1}
HRMS (ESI): *m/z* calcd. for $C_{45}H_{88}O_7NaSi_3$ $[M+Na]^+$ 847.5730, found 847.5729
$[\alpha]^{25}_D$: +10.7 (*c* = 0.7 CH_2Cl_2)

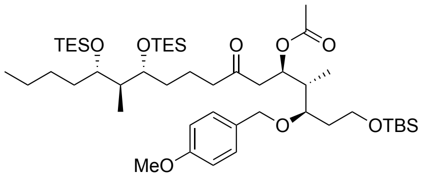

(7*R*,8*R*,9*R*,15*R*,16*S*,17*S*)-17-Butyl-19,19 diethyl-7-((4-methoxybenzyl)oxy)-2,2,3,3,8,16-hexamethyl-11-oxo-15-((triethylsilyl)oxy)-4,18-dioxa-3,19-disilahenicosan-9-yl acetate (S7)

To a solution of aldol adduct **28** (0.020 g, 0.024 mmol) in DCM (0.2 mL) was added triethylamine (0.020 mL, 0.15 mmol), acetic anhydride (0.009 mL, 0.097 mmol), and DMAP (0.30 mg, 2.4 ug). The resulting solution was stirred for 1 h then was quenched with aqueous sodium bicarbonate. The layers were separated and the aqueous layer was extracted with DCM. The combined organic layers were washed with brine and dried over $MgSO_4$ then concentrated. The crude material was purified via flash chromatography (15% Et_2O in hexanes). The desired material was isolated as a yellow oil (0.017 g, 81%).

^{1}H NMR (400 MHz, C_6D_6): δ 7.38 (d, *J* = 8.6 Hz, 2H), 6.84 (d, *J* = 8.7 Hz, 2H), 5.84 – 5.77 (m, 1H), 4.48 (s, 2H), 3.94 – 3.86 (m, 2H), 3.85 – 3.77 (m, 1H), 3.77 – 3.67 (m, 1H), 3.63 – 3.57 (m, 1H), 3.32 (s, 3H). 2.63 (dd, *J* = 15.3, 7.2 Hz, 1H), 2.45 (dd, *J* = 15.3, 5.8 Hz, 1H), 2.39 – 2.20 (m, 2H), 2.16 – 2.07 (m, 1H). 2.07 – 1.98 (m, 1H), 1.96 – 1.86 (m, 1H), 1.86- 1.77 (m, 2H), 1.74 (s, 3H), 1.51 – 1.44 (m, 5H), 1.42 – 1.33 (m, 4H), 1.09 (t, *J* = 7.91 Hz, 9 H), 1.08 (t, *J* = 7.91 Hz, 9H), 0.99 (s, 12H), 0.96 (m, 6H), 0.72 (q, *J* = 7.93 Hz, 6H), 0.71 (q, *J* = 7.93 Hz, 6H), 0.08 (s, 6H)

^{13}C NMR (500 MHz, C_6D_6): δ 206.2, 169.6, 159.5, 129.6, 113.8, 76.8, 73.2, 71.4 70.3, 59.4, 54.5, 45.5, 44.0, 43.1, 39.5, 34.0, 32.5, 27.0, 25.9, 23.3, 20.4, 19.1, 18.1, 14.1, 10.1, 9.75, 7.02, 5.40, –5.53
IR (ATR, neat): 2954, 1739, 1514, 1462, 1245, 1083, 1015, 835, 739 cm^{-1}
HRMS (ESI): *m/z* calcd. for $C_{47}H_{91}O_8Si_3$ $[M+H]^+$ 867.6016, found 867.6039
$[\alpha]^{25}_D$: +8.9 (*c* = 0.45 CH_2Cl_2)

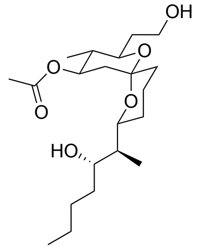

(2*R*,3*R*,4*R*,6*S*,8*R*)-2-(2-hydroxyethyl)-8-((2*S*,3*S*)-3-hydroxyheptan-2-yl)-3-methyl-1,7-dioxaspiro[5.5]undecan-4-yl acetate (30)

Compound **S7** (15.6 mg, 18 μM) was dissolved in a 10:1 mixture of DCM and pH = 7 buffer (0.17 mL). DDQ (5.0 mg, 22 μM) was then added and the resulting solution was stirred for 1 h. Upon completion, the reaction was quenched with sat. aq. $NaHCO_3$ and extracted with DCM and dried over $MgSO_4$. The solution was concentrated and filtered through a silica plug. To a solution of resulting hemiacetal (10.6 mg, 0.0142 mmol) in a 2:1 mixture of MeOH/DCM (2.1 mL) was added *p*-TsOH•H_2O (0.50 mg, 0.0028 mmol). The resulting solution was then allowed to stir. Product formation was seen within 40 min and, to aid in epimerization, additional *p*-TsOH•H_2O was added after 1.5 h (0.50 mg, .0028 mmol) and 2.5 h 0.500 mg, 0.00284 mmol). After a total of 4 h NEt_3 was added and the solution was concentrated. The resulting oil was purified via column chromatography on silica gel eluting with 30%-50% EtOAc/Hexanes to afford the two epimers of the spirocyclic (3.0 mg major, 8.05 μmol 55%, 1.5 mg minor, contaminated with **29**, 4.03 μmol 27.5%, 82% overall). The structure of the minor diastereomer was assigned by analogy and was not characterized due to the lack of material.

^{1}H NMR (600 MHz, C_6D_6): δ 4.88 (td, *J* = 5.2 Hz, 1H), 4.05 (td, *J* = 4.4 Hz, 1H), 3.98 – 3.93 (m, 1H), 3.81 – 3.72 (m, 2H), 3.52 (dd, *J* = 9.0, 2.2 Hz, 1H), 2.74 (dd, *J* = 12.8, 4.4 Hz, 1H), 2.39 (br s, 2H), 1.78 – 1.69 (m, 4H), 1.68 (s, 3H), 1.59 – 1.47 (m, 4H), 1.47 – 1.38 (m, 4H), 1.37 – 1.30 (m, 3H), 1.22 – 1.11 (m, 1H), 1.00 (t, *J* = 7.2 Hz, 3H), 0.97 – 0.90 (m, 1H), 0.769 (d, *J* = 6.4 Hz, 3H), 0.745 (d, *J* = 7.0 Hz, 3H), 0.49 (water)
^{13}C NMR (600 MHz, C_6D_6): δ 169.9, 98.9, 76.9, 74.7, 73.4, 72.4, 60.6, 45.2, 40.7, 35.6, 35.2, 34.1, 32.9, 28.6, 28.5, 23.0, 20.3, 19.7, 14.3, 12.7, 11.4
IR (ATR, neat): 3358, 2928, 1728, 1637, 1243, 1023, 610
HRMS (ESI): *m/z* calcd. for $C_{21}H_{39}O_6$ $[M+H]^+$ 387.2768, found 387.2741
$[\alpha]^{25}_D$: +73.8 (*c* = 0.15 CH_2Cl_2)

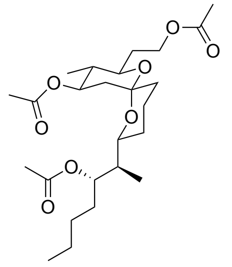

2-((2*R*,3*R*,4*R*,6*S*,8*R*)-4-Acetoxy-8-((2*R*,3*S*)-3-acetoxyheptan-2-yl)-3-methyl-1,7-dioxaspiro[5.5]undecan-2-yl)ethyl acetate (31)

Spirocycle **30** (3.00 mg, 8.05 μmol) was dissolved in DCM (0.1 mL) and Ac_2O (6.00 μL, 64.4 μmol), NEt_3 (13.5 μL, 96.6 μmol), and DMAP (0.200 mg, 1.61 μmol) were added. The resulting solution was stirred for 30 minutes then quenched with sat. aq. NH_4Cl. The layers were separated and the aqueous layer was extracted with DCM. The combined organic layers were dried over $MgSO_4$, filtered, and concentrated. The resulting residue was columned on

silica gel eluting with 10-50% Et_2O/hexanes to afford the desired product (3.00 mg, 6.60 μmol, 82%)

^{1}H NMR (600 MHz, C_6D_6) δ 5.61 (ddd, *J* = 10.7, 6.7, 2.0 Hz, 1H), 4.83 (td, *J* = 10.9, 4.5 Hz, 1H), 4.47 – 4.38 (m, 2H), 4.22 (ddd, *J* = 13.7, 7.8, 2.0 Hz, 1H), 3.42 (dd, *J* = 7.9, 2.0 Hz, 1H), 2.74 (dd, *J* = 12.8, 4.8 Hz, 1H), 2.04 – 1.94 (m, 2H), 1.94 – 1.87 (m, 1H), 1.83 (s, 3H), 1.81 (s, 3H), 1.67 (s, 3H), 1.66 – 1.58 (m, 3H), 1.57 – 1.44 (m, 6H), 1.43 – 1.26 (m, 4H), 1.26 – 1.16 (m, 2H), 1.01 (t, *J* = 7.2 Hz, 3H), 0.88 (d, *J* = 6.6 Hz, 3H), 0.81 (d, *J* = 7.3 Hz, 3H), 0.43 (water)

^{13}C NMR (600 MHz, C_6D_6) δ 169.9, 169.7, 169.5, 98.1, 74.0, 73.1, 70.9, 61.0, 42.9, 40.4, 35.2, 34.4, 32.2, 29.1, 28.8, 27.9, 22.8, 20.6, 20.3, 19.7, 14.2, 12.7, 10.2,

IR (ATR Neat) 2929, 1735, 1461, 1337, 1239, 1022, 606

HRMS (ESI) *m/z* calcd. for $C_{24}H_{40}O_8$ $[M+H]^+$ 456.2723, found

[α]$^{25}_{D}$: +52.8 (*c* = 0.300 CH_2Cl_2)

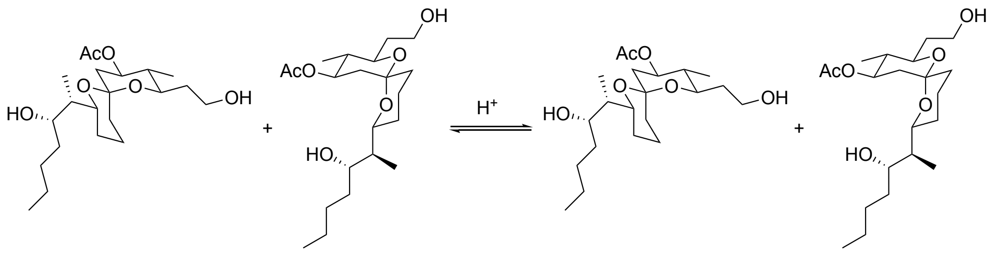

2:1 mixture 1:2.2 mixture

A 2:1 (undesired: desired) mixture of diastereomers (1.5 mg, 3.9 μmol) was dissolved in a 2:1 mixture of MeOH/DCM (0.39 mL) and *p*TsOH•H_2O (0.3 mg, 1.6 μmol) was added. The solution was stirred for 6 h, then was quenched with NEt_3 and concentrated. The resulting residue was filtered through a silica plug eluting (100% Et_2O) to afford a 2.2:1 (desired: undesired) mixture of diastereomers.

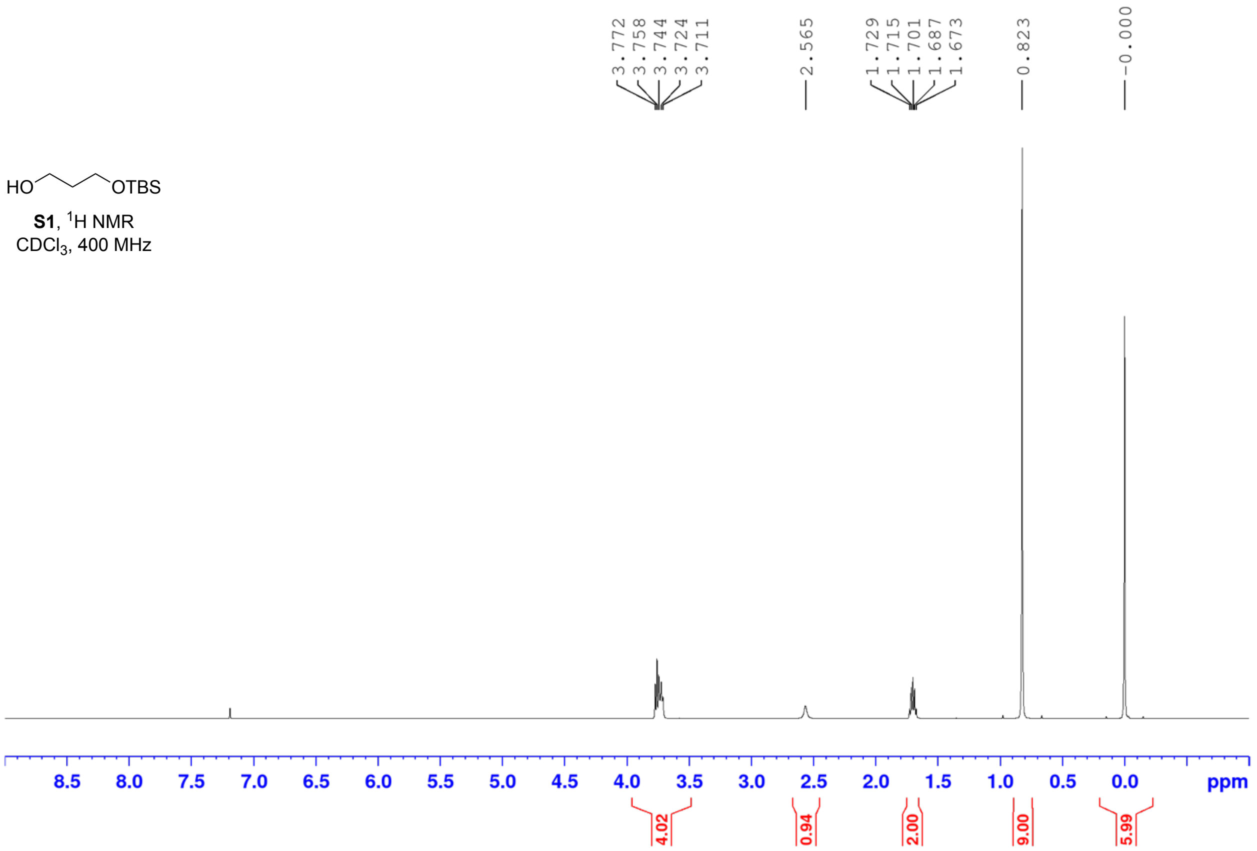

3.772
3.758
3.744
3.724
3.711
2.565
1.729
1.715
1.701
1.687
1.673
0.823
-0.000
HO OTBS
S1, ¹H NMR
CDCl₃, 400 MHz
8.5 8.0 7.5 7.0 6.5 6.0 5.5 5.0 4.5 4.0 3.5 3.0 2.5 2.0 1.5 1.0 0.5 0.0 ppm
4.02
0.94
2.00
9.00
5.99

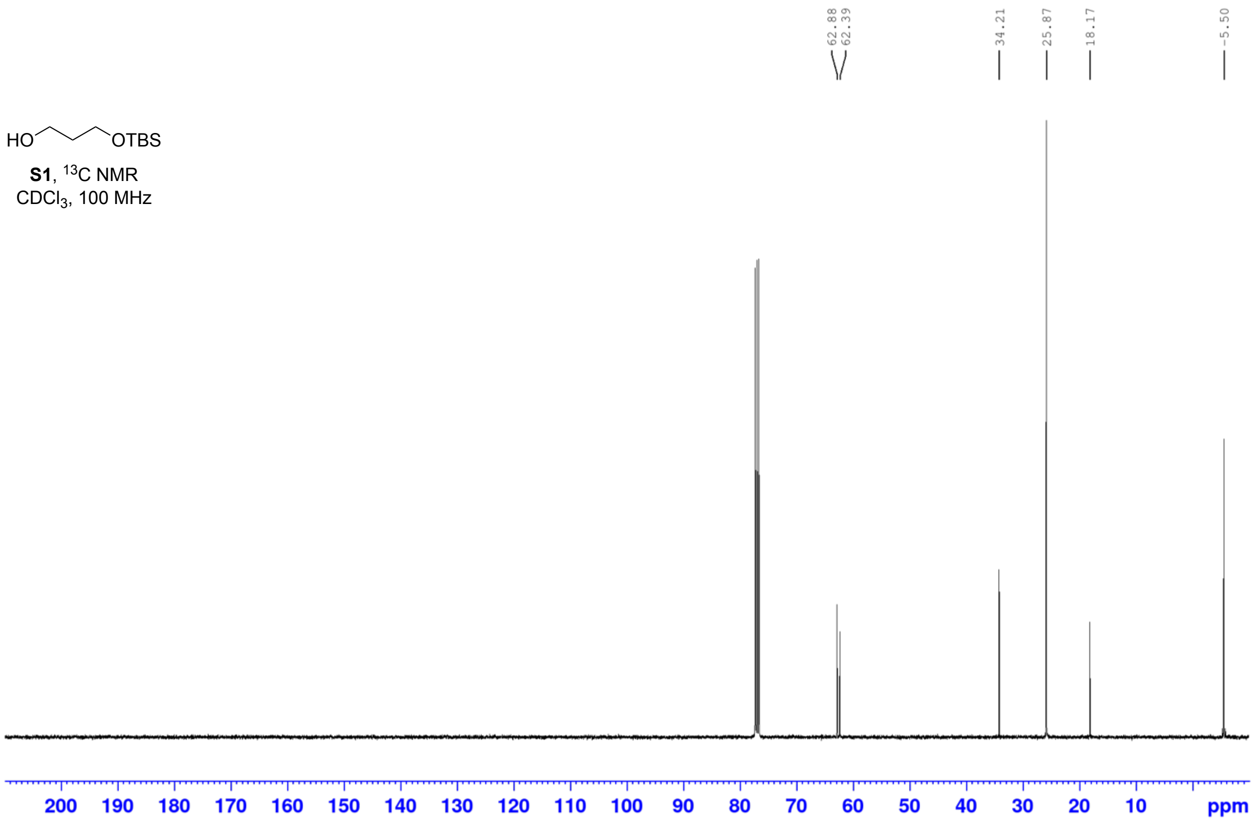

62.88
62.39
34.21
25.87
18.17
-5.50
HO OTBS
S1, ¹³C NMR
CDCl₃, 100 MHz
200 190 180 170 160 150 140 130 120 110 100 90 80 70 60 50 40 30 20 10 ppm

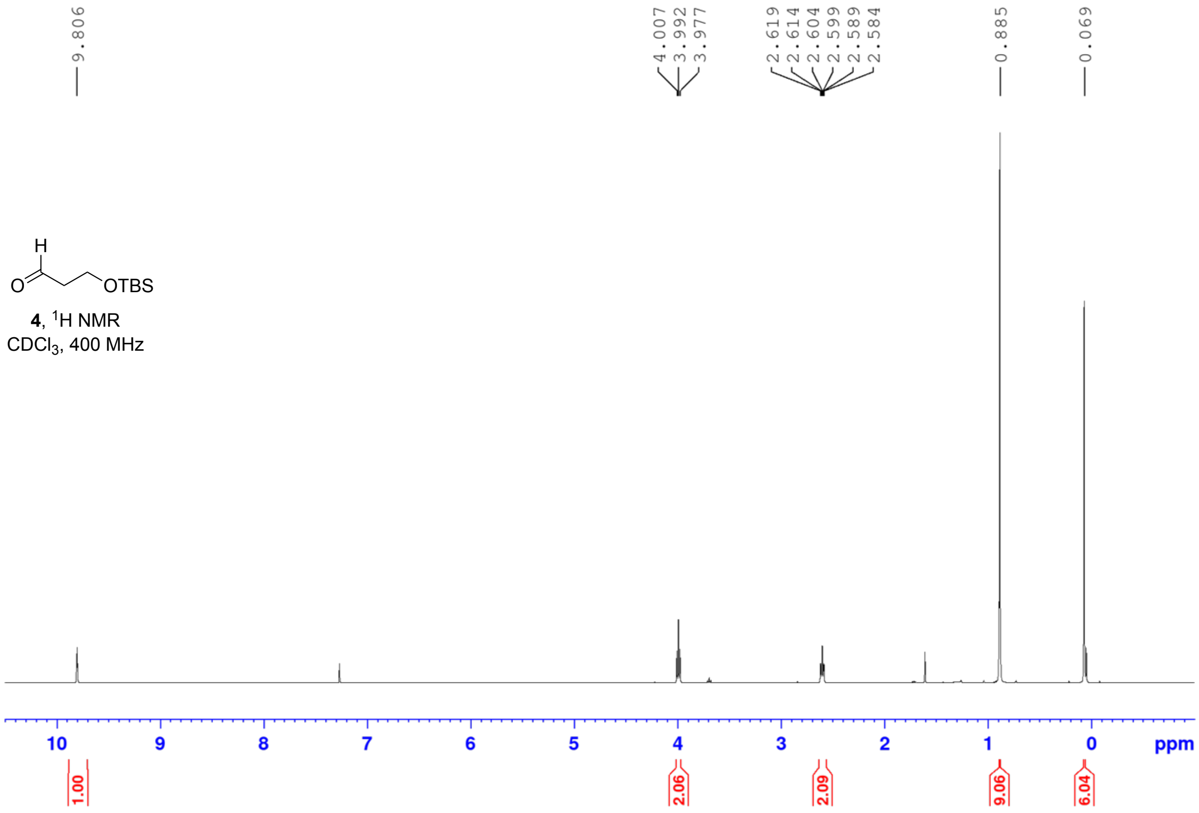
9.806
4.007
3.992
3.977
2.619
2.614
2.604
2.599
2.589
2.584
0.885
0.069
H
O
OTBS
4, ¹H NMR
CDCl3, 400 MHz
10
9
8
7
6
5
4
3
2
1
0
ppm
1.00
2.06
2.09
9.06
6.04

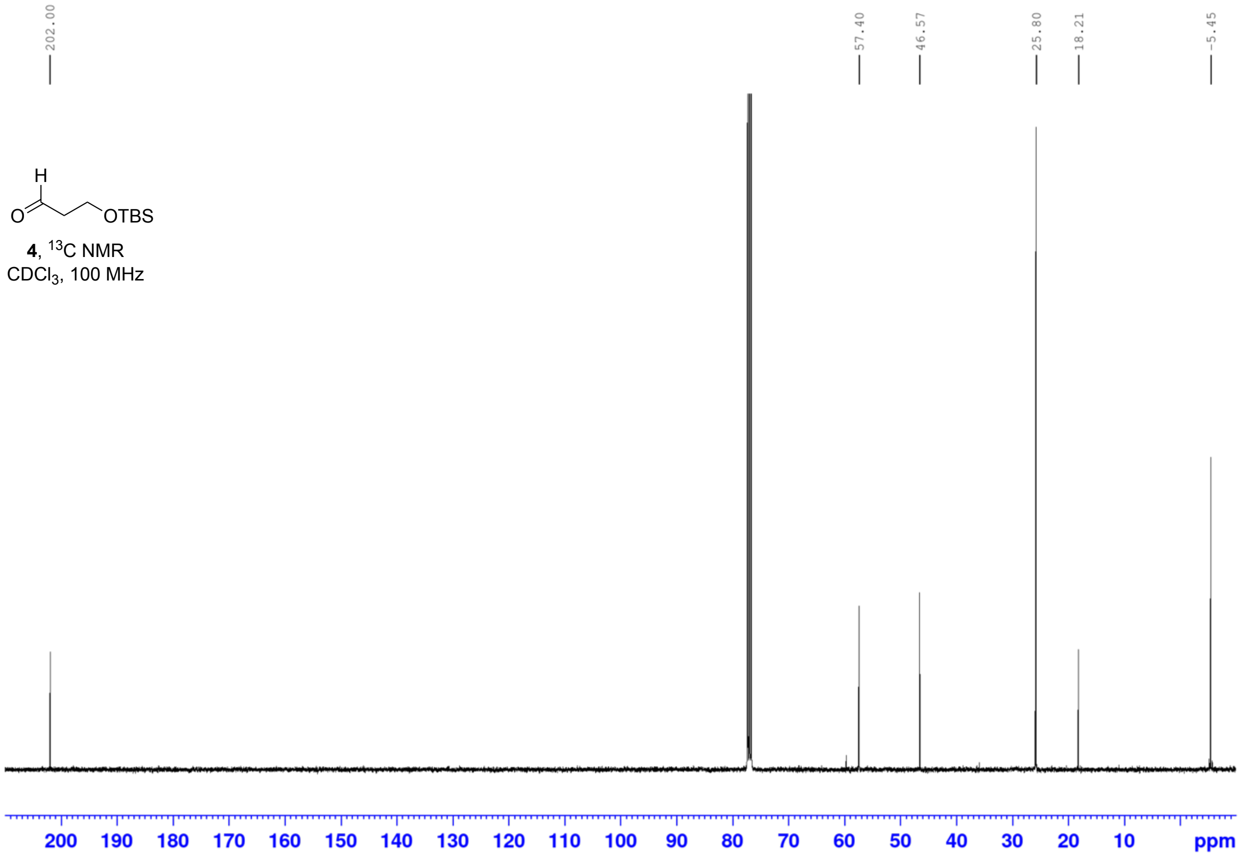
202.00
57.40
46.57
25.80
18.21
-5.45
H
O
OTBS
4, ¹³C NMR
CDCl3, 100 MHz
200
190
180
170
160
150
140
130
120
110
100
90
80
70
60
50
40
30
20
10
ppm

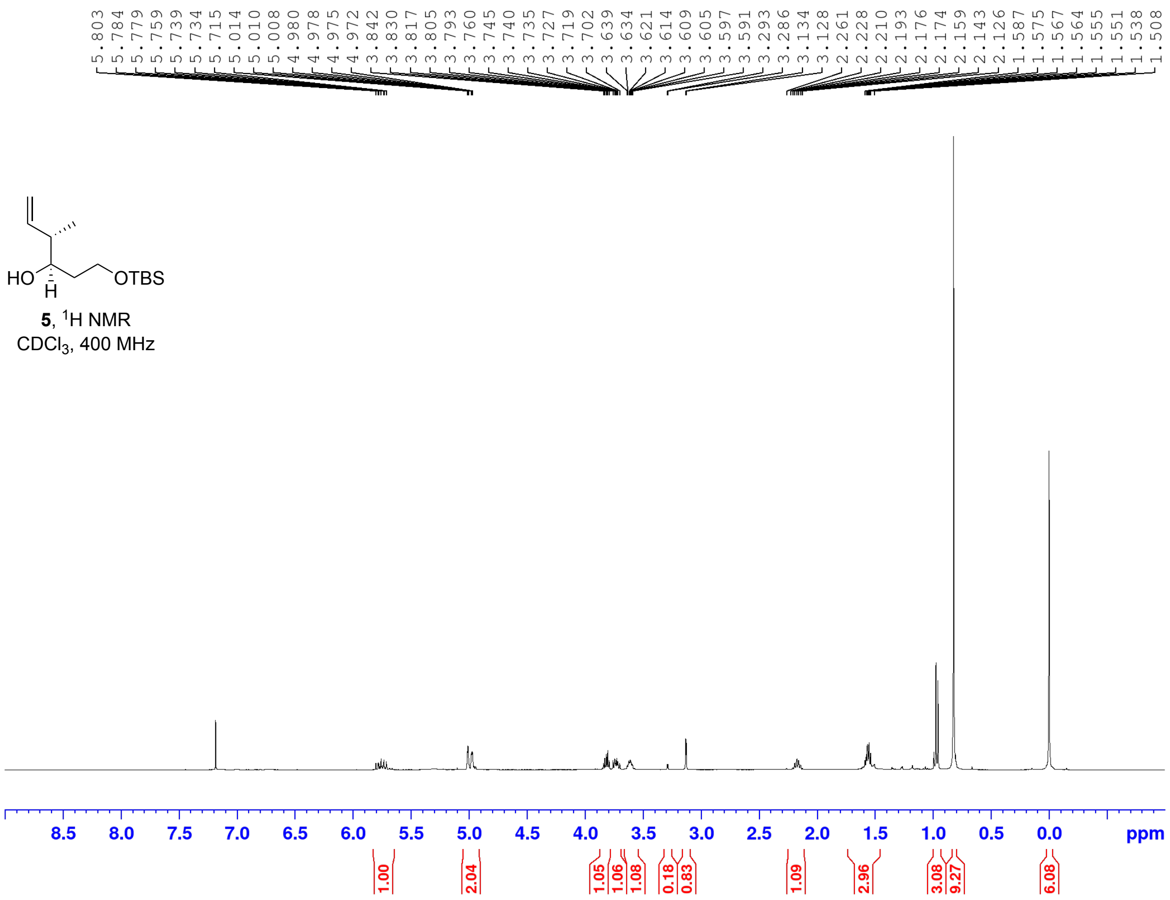
5.803
5.784
5.779
5.759
5.739
5.734
5.715
5.014
5.010
5.008
4.980
4.978
4.975
4.972
3.842
3.830
3.817
3.805
3.793
3.760
3.745
3.740
3.735
3.727
3.719
3.702
3.639
3.634
3.621
3.614
3.609
3.605
3.597
3.591
3.293
3.286
3.134
3.128
2.261
2.228
2.210
2.193
2.176
2.174
2.159
2.143
2.126
1.587
1.575
1.567
1.564
1.555
1.551
1.538
1.508
HO
H
OTBS
5, ^{1}H NMR
$CDCl_3$, 400 MHz
8.5 8.0 7.5 7.0 6.5 6.0 5.5 5.0 4.5 4.0 3.5 3.0 2.5 2.0 1.5 1.0 0.5 0.0 ppm
1.00
2.04
1.05
1.06
1.08
0.18
0.83
1.09
2.96
3.08
9.27
6.08

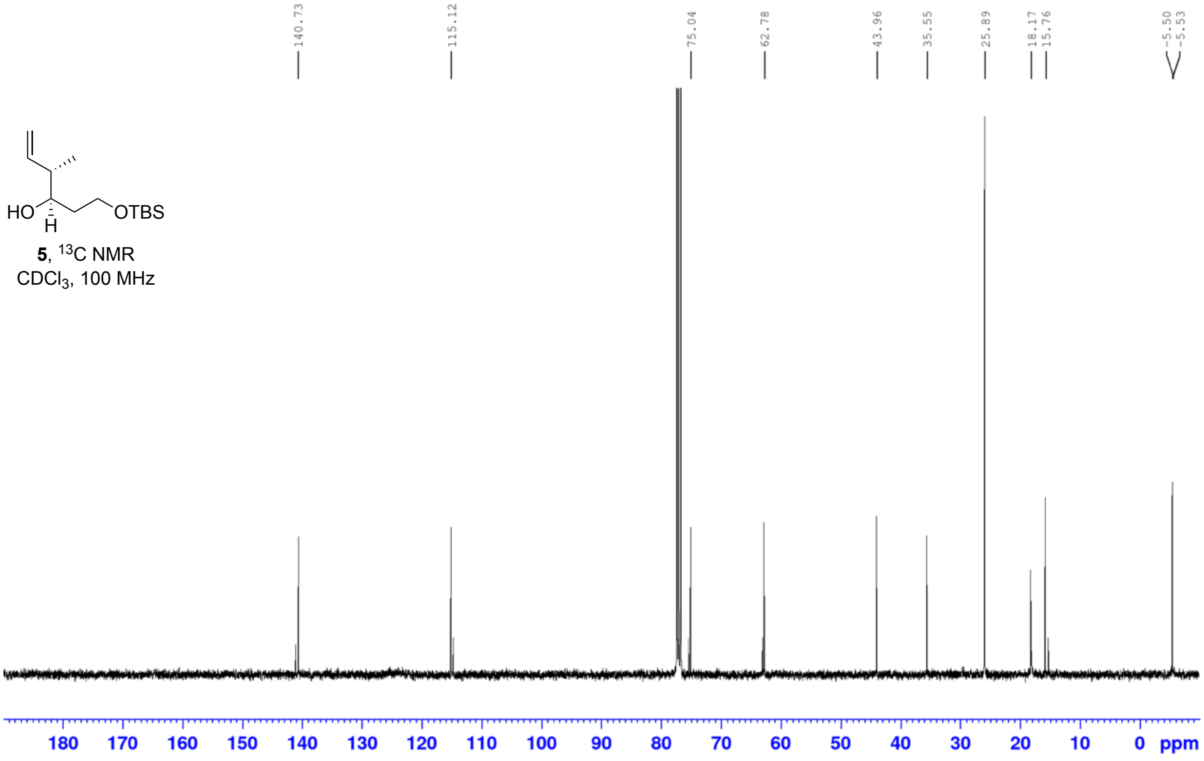
140.73
115.12
75.04
62.78
43.96
35.55
25.89
18.17
15.76
-5.50
-5.53
HO
H
OTBS
5, ^{13}C NMR
$CDCl_3$, 100 MHz
180 170 160 150 140 130 120 110 100 90 80 70 60 50 40 30 20 10 0 ppm

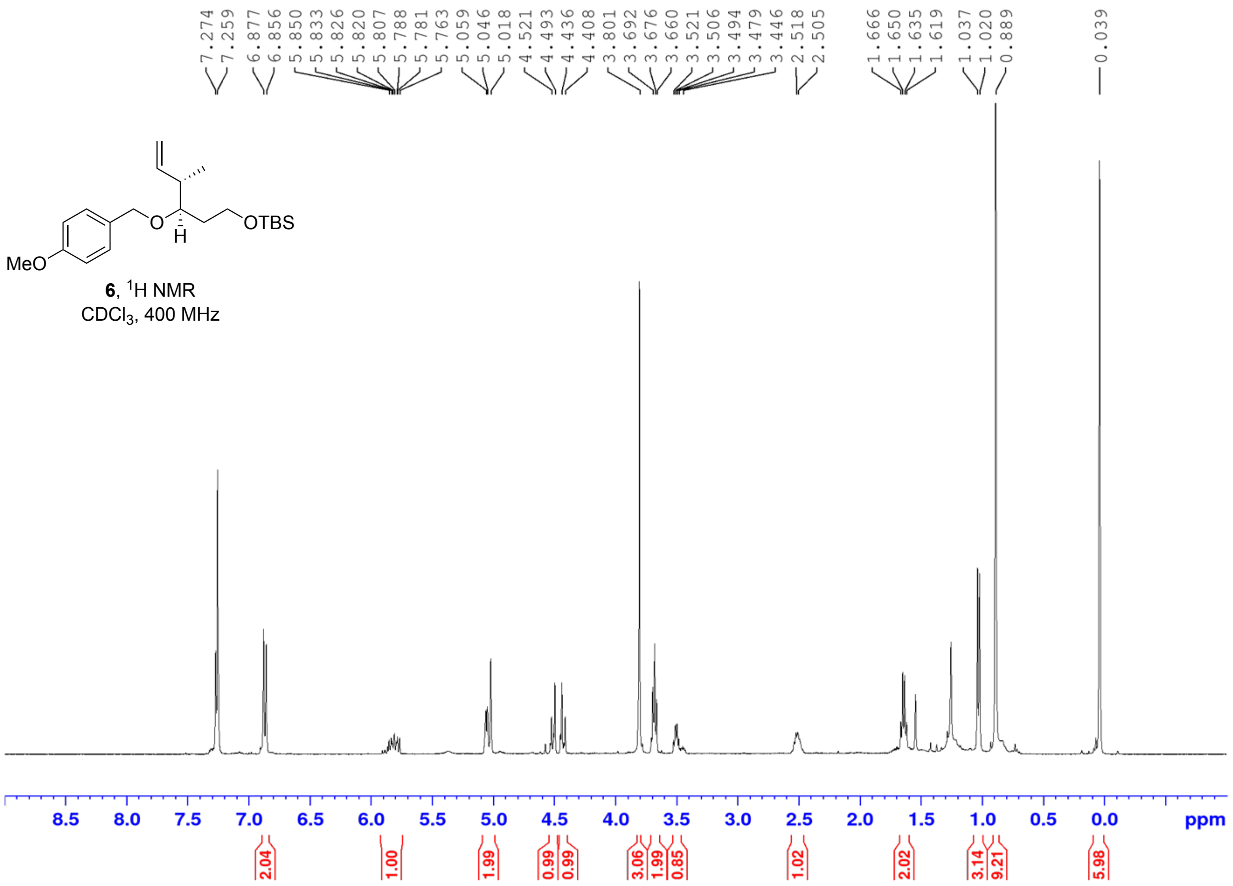
7.274
7.259
6.877
6.856
5.850
5.833
5.826
5.820
5.807
5.788
5.781
5.763
5.059
5.046
5.018
4.521
4.493
4.436
4.408
3.801
3.692
3.676
3.660
3.521
3.506
3.494
3.479
3.446
2.518
2.505
1.666
1.650
1.635
1.619
1.037
1.020
0.889
0.039
OTBS
MeO
O
H
6, 1H NMR
CDCl3, 400 MHz
8.5
8.0
7.5
7.0
6.5
6.0
5.5
5.0
4.5
4.0
3.5
3.0
2.5
2.0
1.5
1.0
0.5
0.0
ppm
2.04
1.00
1.99
0.99
0.99
3.06
1.99
0.85
1.02
2.02
3.14
9.21
5.98

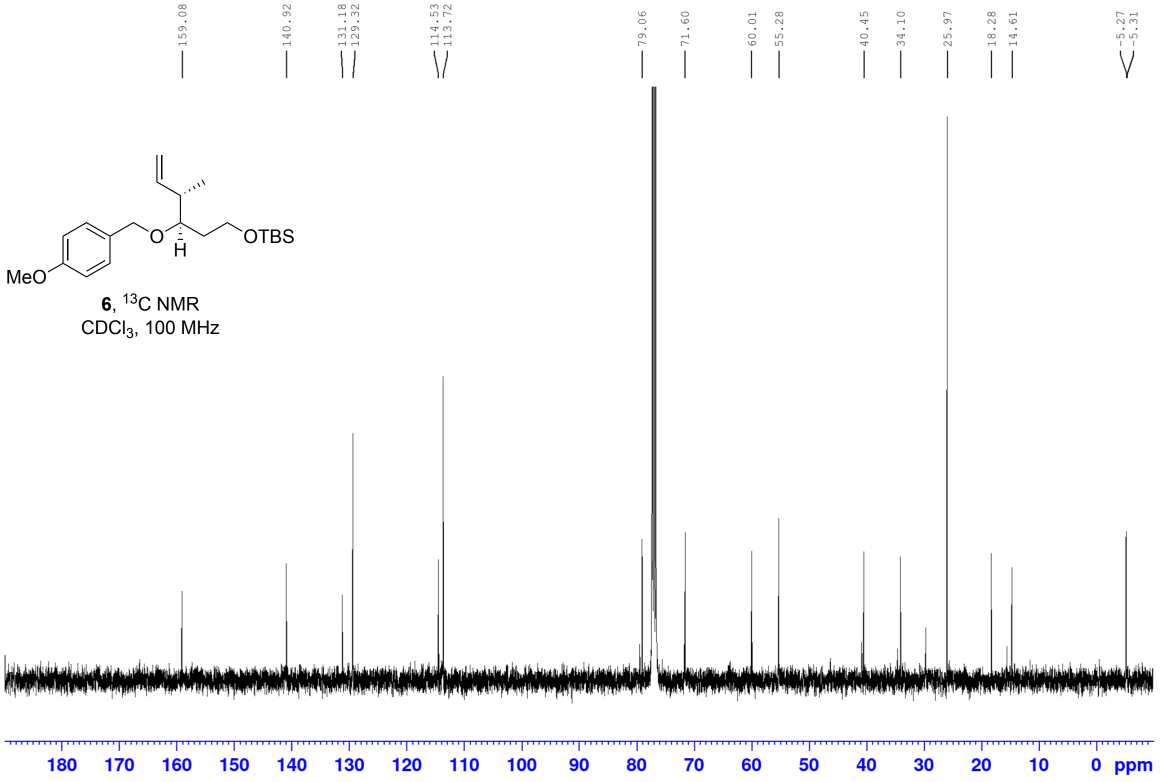
159.08
140.92
131.18
129.32
114.53
113.72
79.06
71.60
60.01
55.28
40.45
34.10
25.97
18.28
14.61
-5.27
-5.31
OTBS
MeO
O
H
6, 13C NMR
CDCl3, 100 MHz
180
170
160
150
140
130
120
110
100
90
80
70
60
50
40
30
20
10
0
ppm

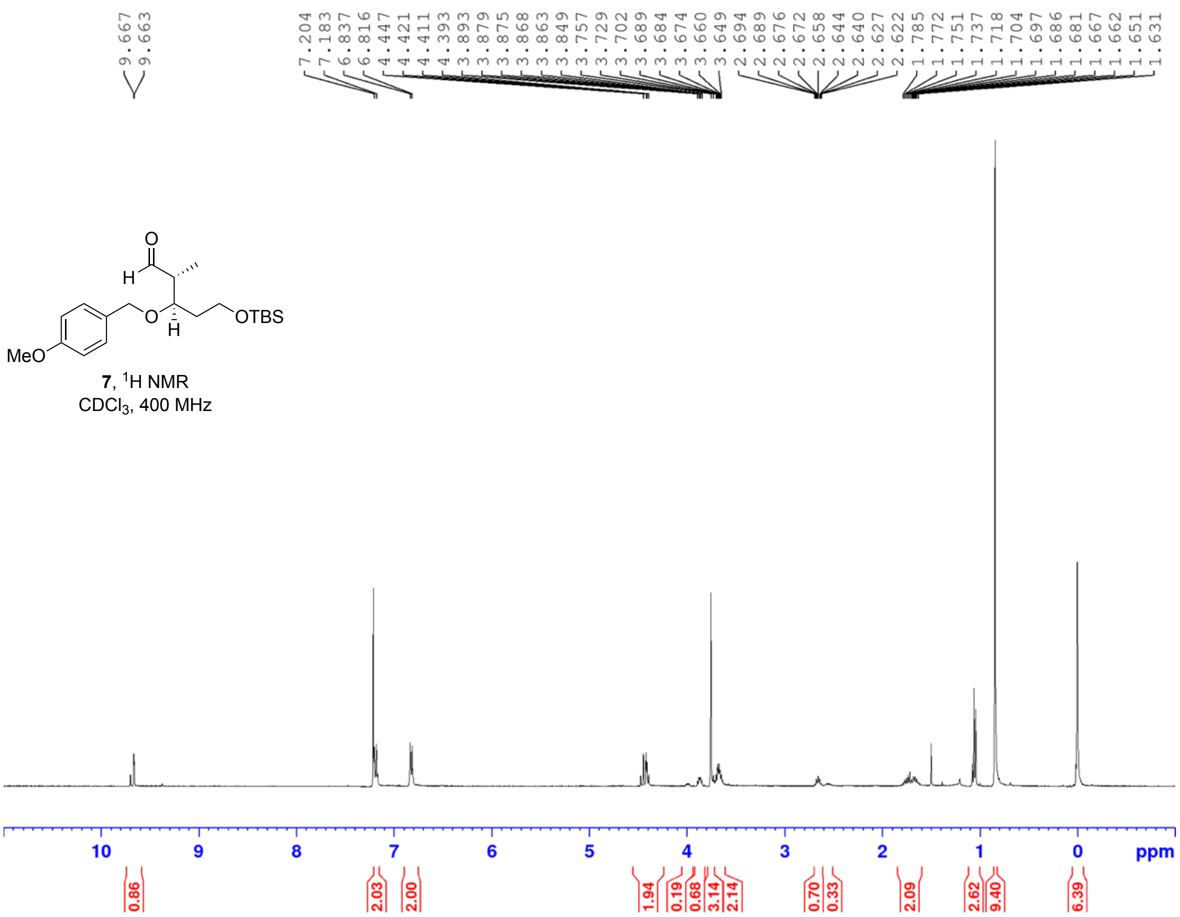
9.667
9.663
7.204
7.183
6.837
6.816
4.447
4.421
4.411
4.393
3.893
3.879
3.875
3.868
3.863
3.849
3.757
3.729
3.702
3.689
3.684
3.674
3.660
3.649
2.694
2.689
2.676
2.672
2.658
2.644
2.640
2.627
2.622
1.785
1.772
1.751
1.737
1.718
1.704
1.697
1.686
1.681
1.667
1.662
1.651
1.631
O
H
MeO
O
H
OTBS
7, ¹H NMR
CDCl₃, 400 MHz
10
9
8
7
6
5
4
3
2
1
0
ppm
0.86
2.03
2.00
1.94
0.19
0.68
3.14
2.14
0.70
0.33
2.09
2.62
9.40
6.39

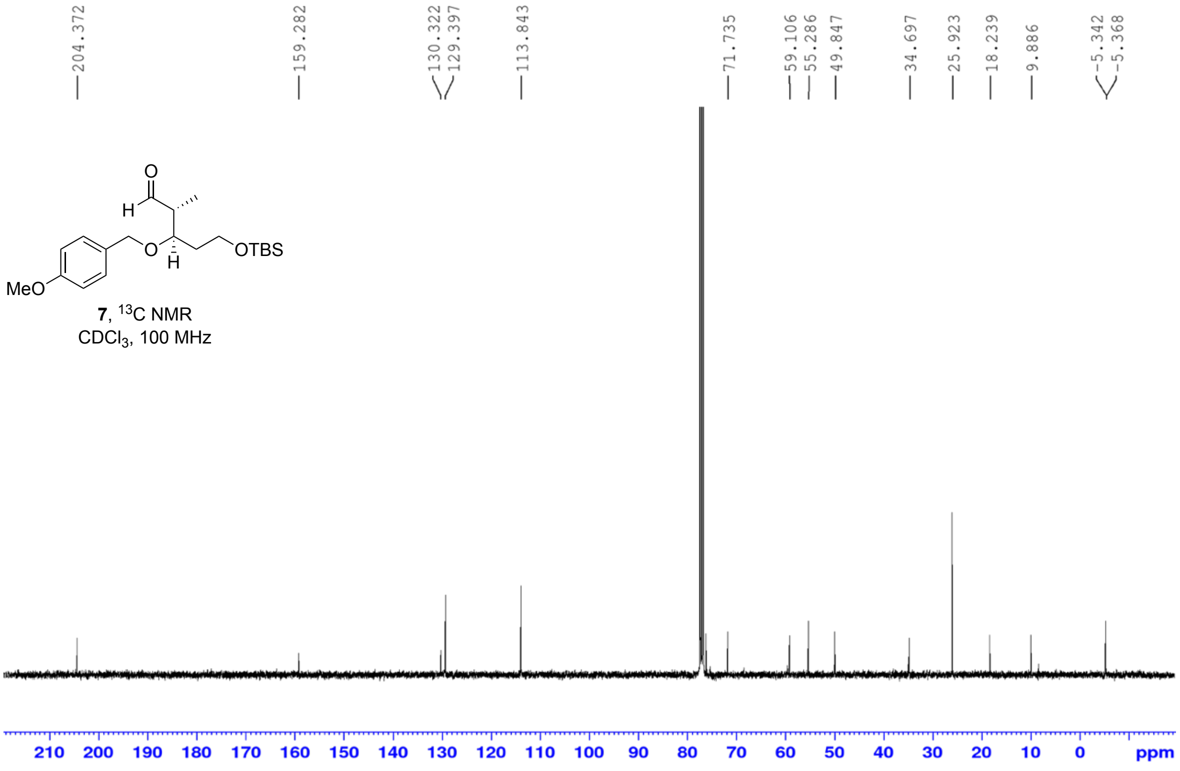
204.372
159.282
130.322
129.397
113.843
71.735
59.106
55.286
49.847
34.697
25.923
18.239
9.886
-5.342
-5.368
O
H
MeO
O
H
OTBS
7, ¹³C NMR
CDCl₃, 100 MHz
210
200
190
180
170
160
150
140
130
120
110
100
90
80
70
60
50
40
30
20
10
0
ppm

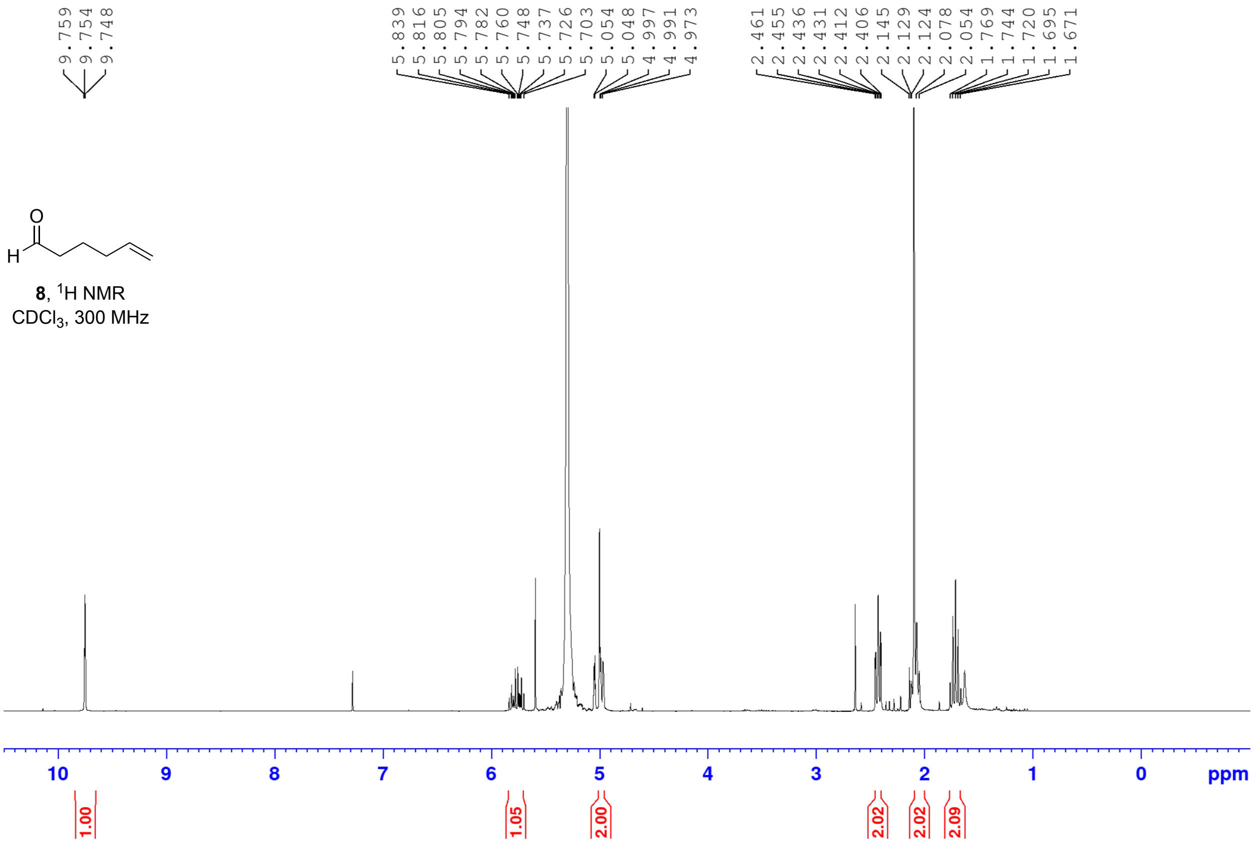
9.759
9.754
9.748
5.839
5.816
5.805
5.794
5.782
5.760
5.748
5.737
5.726
5.703
5.054
5.048
4.997
4.991
4.973
2.461
2.455
2.436
2.431
2.412
2.406
2.145
2.129
2.124
2.078
2.054
1.769
1.744
1.720
1.695
1.671
O
H
8, ^{1}H NMR
$CDCl_3$, 300 MHz
10
9
8
7
6
5
4
3
2
1
0
ppm
1.00
1.05
2.00
2.02
2.02
2.09

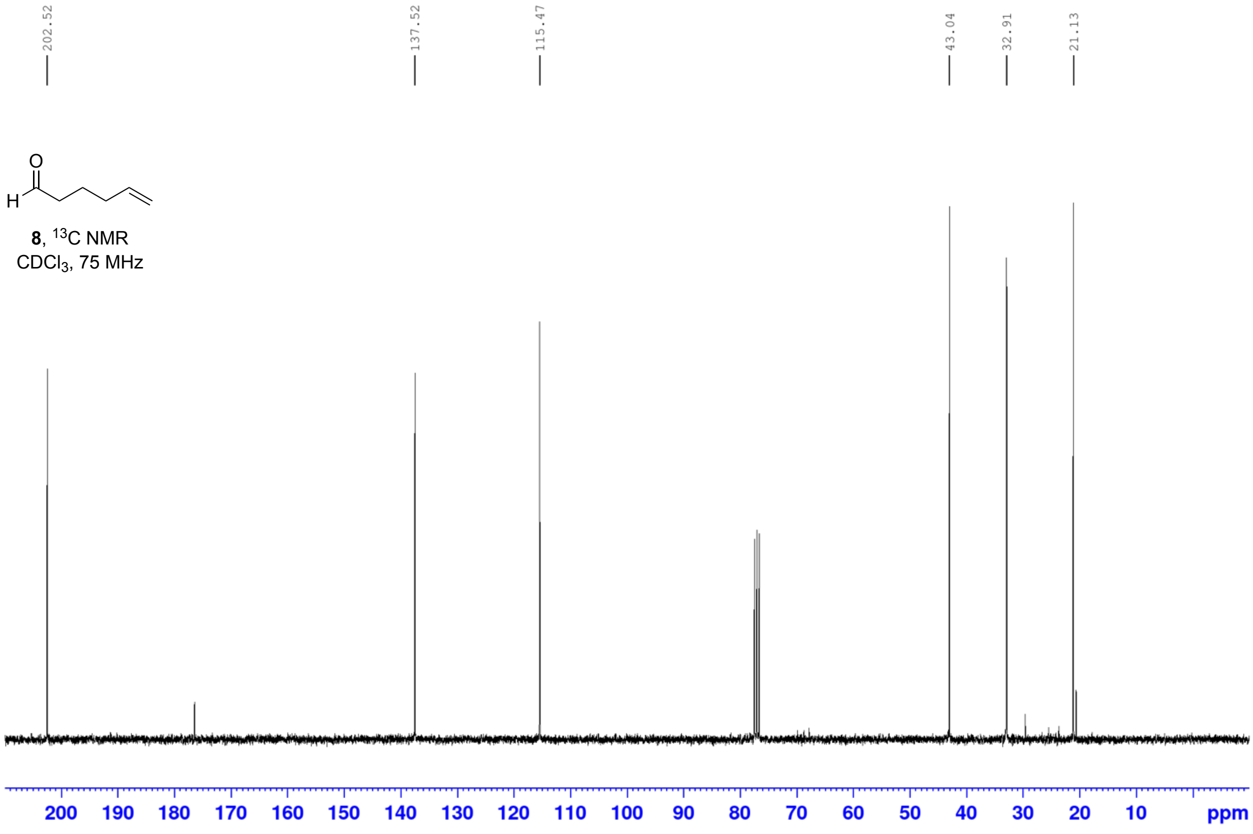
202.52
137.52
115.47
43.04
32.91
21.13
O
H
8, ^{13}C NMR
$CDCl_3$, 75 MHz
200
190
180
170
160
150
140
130
120
110
100
90
80
70
60
50
40
30
20
10
ppm

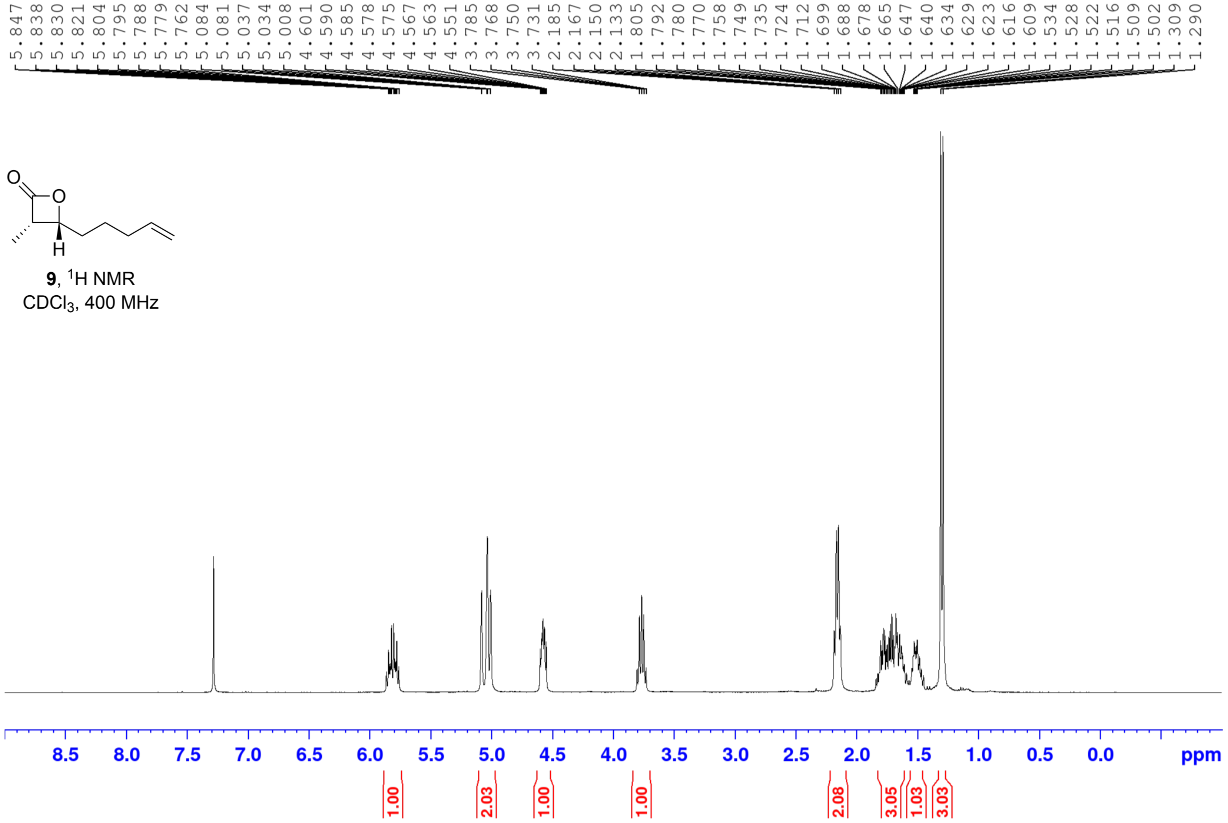
5.847 5.838 5.830 5.821 5.804 5.795 5.788 5.779 5.762 5.084 5.081 5.037 5.034 5.008 4.601 4.590 4.585 4.578 4.575 4.567 4.563 4.551 3.785 3.768 3.750 3.731 2.185 2.167 2.150 2.133 1.805 1.792 1.780 1.770 1.758 1.749 1.735 1.724 1.712 1.699 1.688 1.678 1.665 1.647 1.640 1.634 1.629 1.623 1.616 1.609 1.534 1.528 1.522 1.516 1.509 1.502 1.309 1.290
O
O
H
9, 1H NMR
CDCl3, 400 MHz
8.5 8.0 7.5 7.0 6.5 6.0 5.5 5.0 4.5 4.0 3.5 3.0 2.5 2.0 1.5 1.0 0.5 0.0 ppm
1.00 2.03 1.00 1.00 2.08 3.05 1.03 3.03

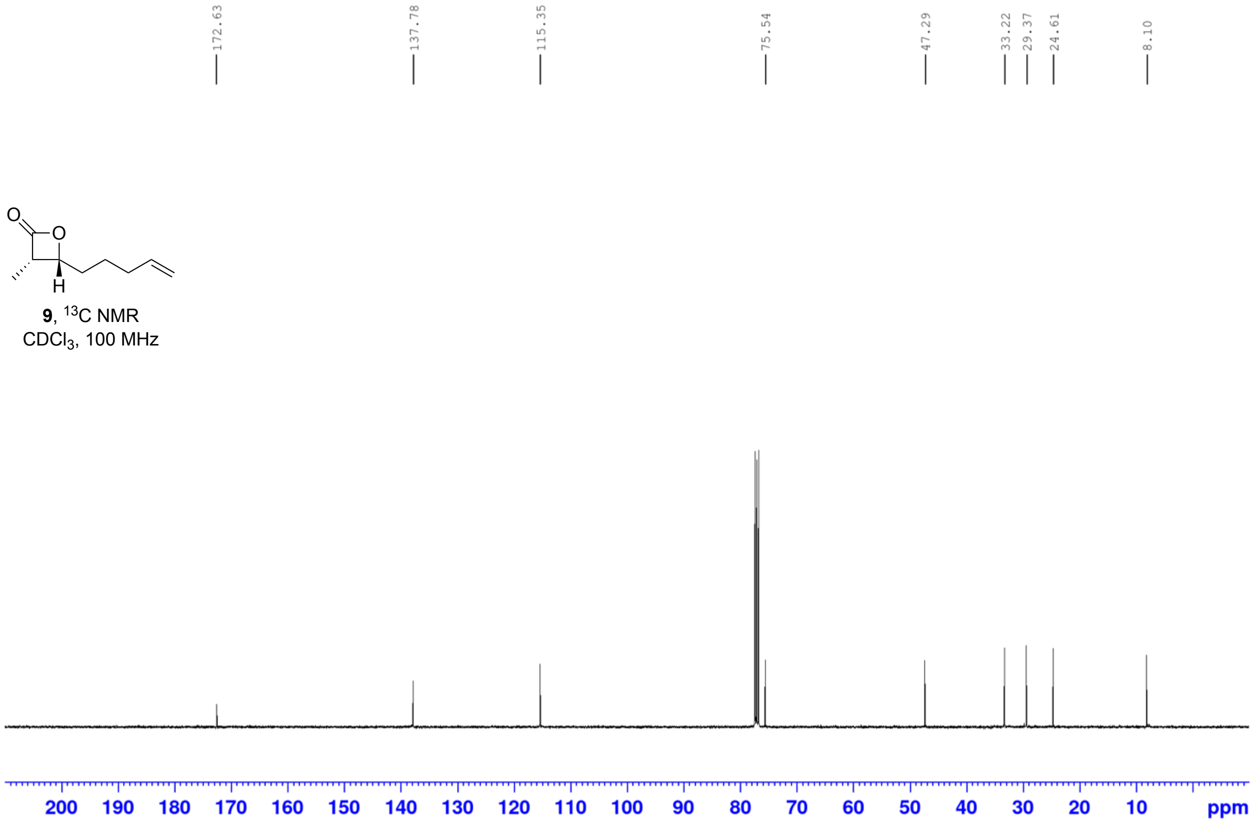
172.63 137.78 115.35 75.54 47.29 33.22 29.37 24.61 8.10
O
O
H
9, 13C NMR
CDCl3, 100 MHz
200 190 180 170 160 150 140 130 120 110 100 90 80 70 60 50 40 30 20 10 ppm

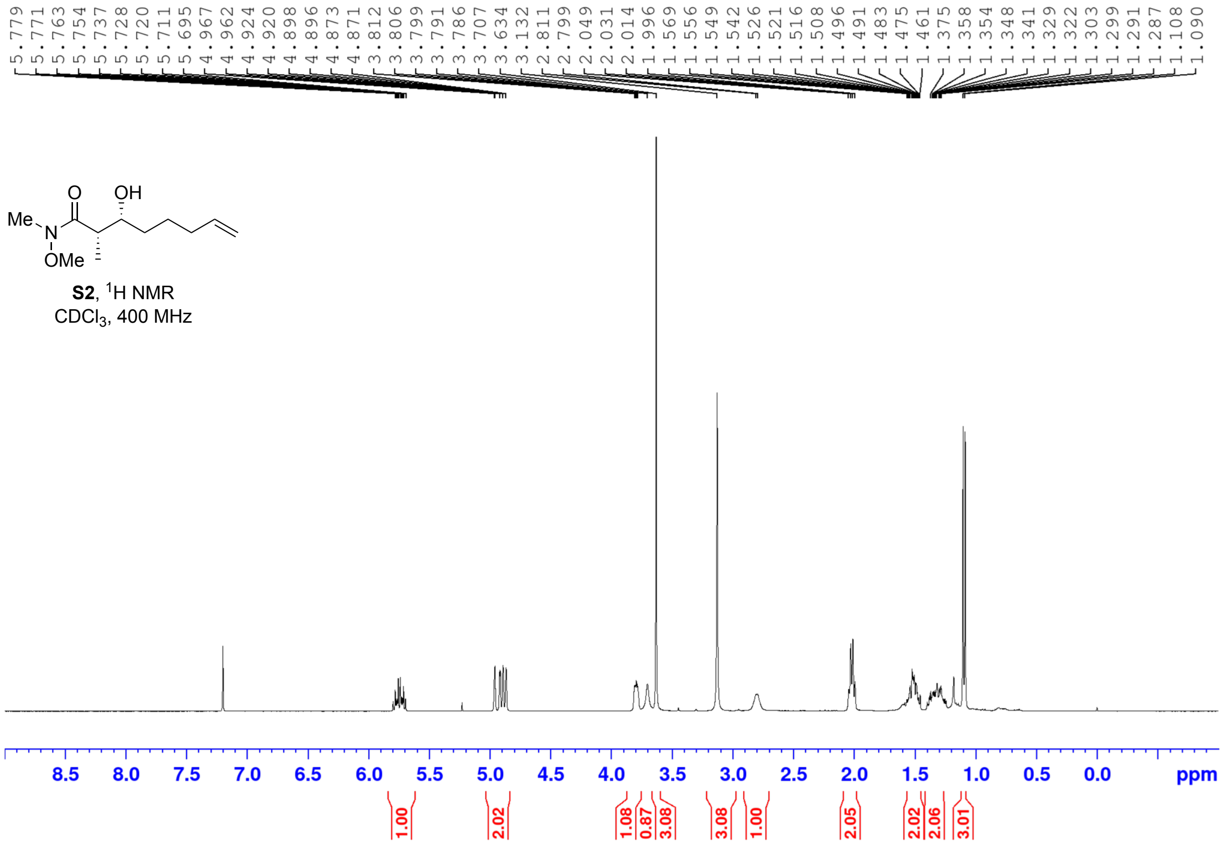
5.779 5.771 5.763 5.754 5.737 5.728 5.720 5.711 5.695 4.967 4.962 4.924 4.920 4.898 4.896 4.873 4.871 3.812 3.806 3.799 3.791 3.786 3.707 3.634 3.132 2.811 2.799 2.049 2.031 2.014 1.996 1.569 1.556 1.549 1.542 1.526 1.521 1.516 1.508 1.496 1.491 1.483 1.475 1.461 1.375 1.358 1.354 1.348 1.341 1.329 1.322 1.303 1.299 1.291 1.287 1.108 1.090
O
OH
Me
N
OMe
S2, 1H NMR
CDCl3, 400 MHz
8.5 8.0 7.5 7.0 6.5 6.0 5.5 5.0 4.5 4.0 3.5 3.0 2.5 2.0 1.5 1.0 0.5 0.0 ppm
1.00 2.02 1.08 0.87 3.08 3.08 1.00 2.05 2.02 2.06 3.01

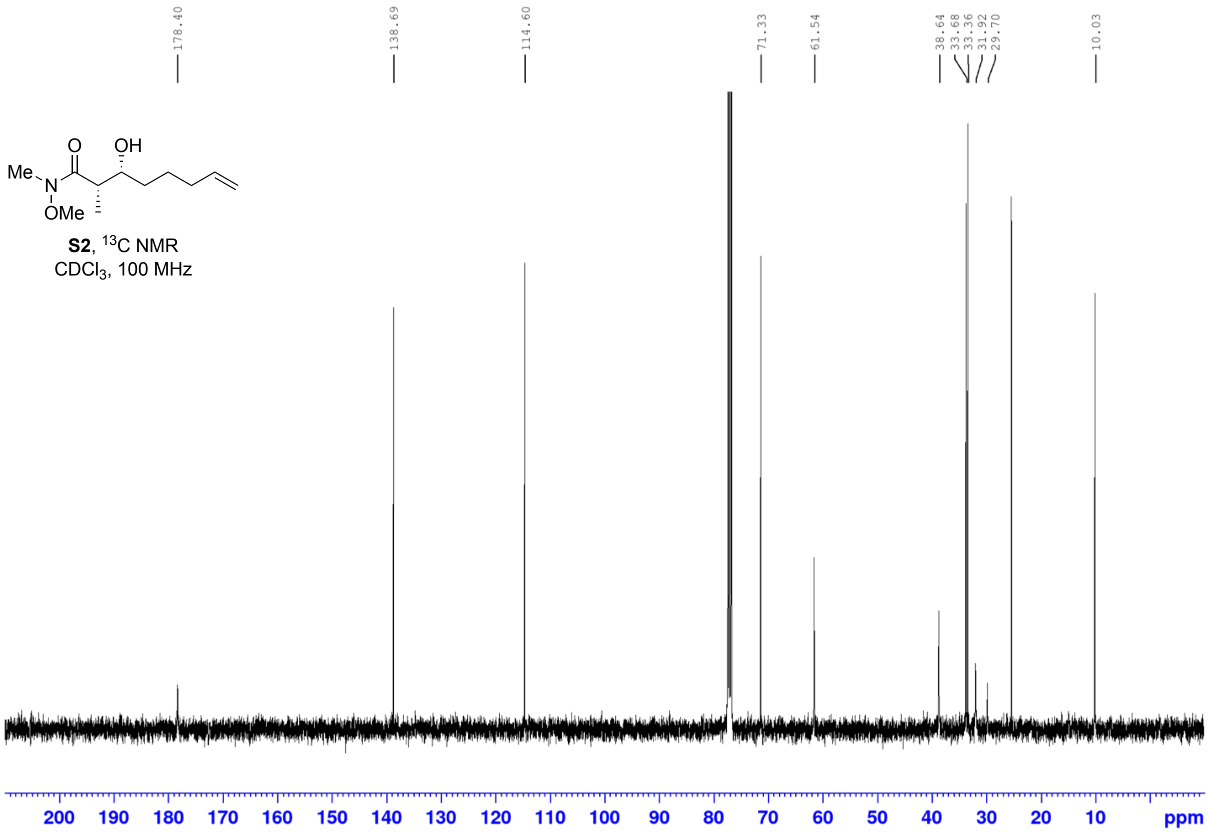
178.40 138.69 114.60 71.33 61.54 38.64 33.68 33.36 31.92 29.70 10.03
O
OH
Me
N
OMe
S2, 13C NMR
CDCl3, 100 MHz
200 190 180 170 160 150 140 130 120 110 100 90 80 70 60 50 40 30 20 10 ppm

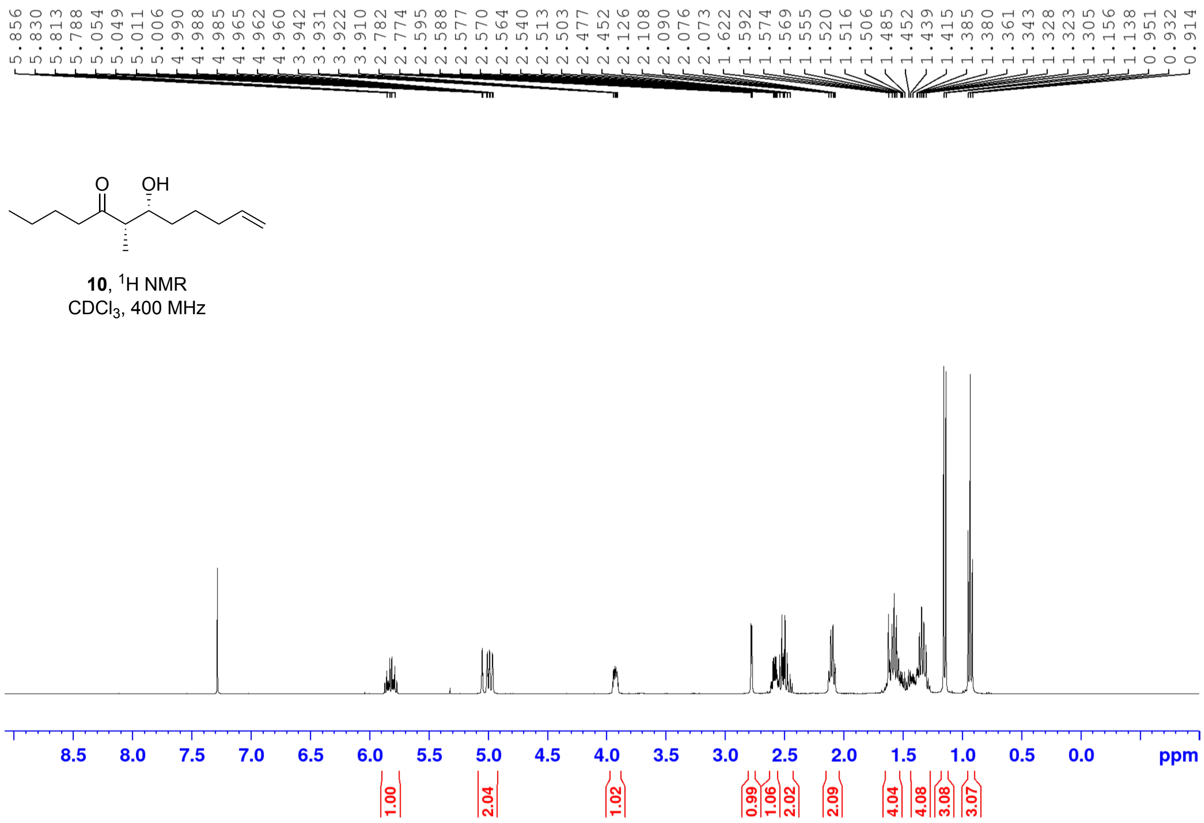
5.856 5.830 5.813 5.788 5.054 5.049 5.011 5.006 4.990 4.988 4.985 4.965 4.962 4.960 3.942 3.931 3.922 3.910 2.782 2.774 2.595 2.588 2.577 2.570 2.564 2.540 2.513 2.503 2.477 2.452 2.126 2.108 2.090 2.076 2.073 1.622 1.592 1.574 1.569 1.555 1.520 1.516 1.506 1.485 1.452 1.439 1.415 1.385 1.380 1.361 1.343 1.328 1.323 1.305 1.156 1.138 0.951 0.932 0.914
O
OH
10, 1H NMR
CDCl3, 400 MHz
8.5 8.0 7.5 7.0 6.5 6.0 5.5 5.0 4.5 4.0 3.5 3.0 2.5 2.0 1.5 1.0 0.5 0.0 ppm
1.00 2.04 1.02 0.99 1.06 2.02 2.09 4.04 4.08 3.08 3.07

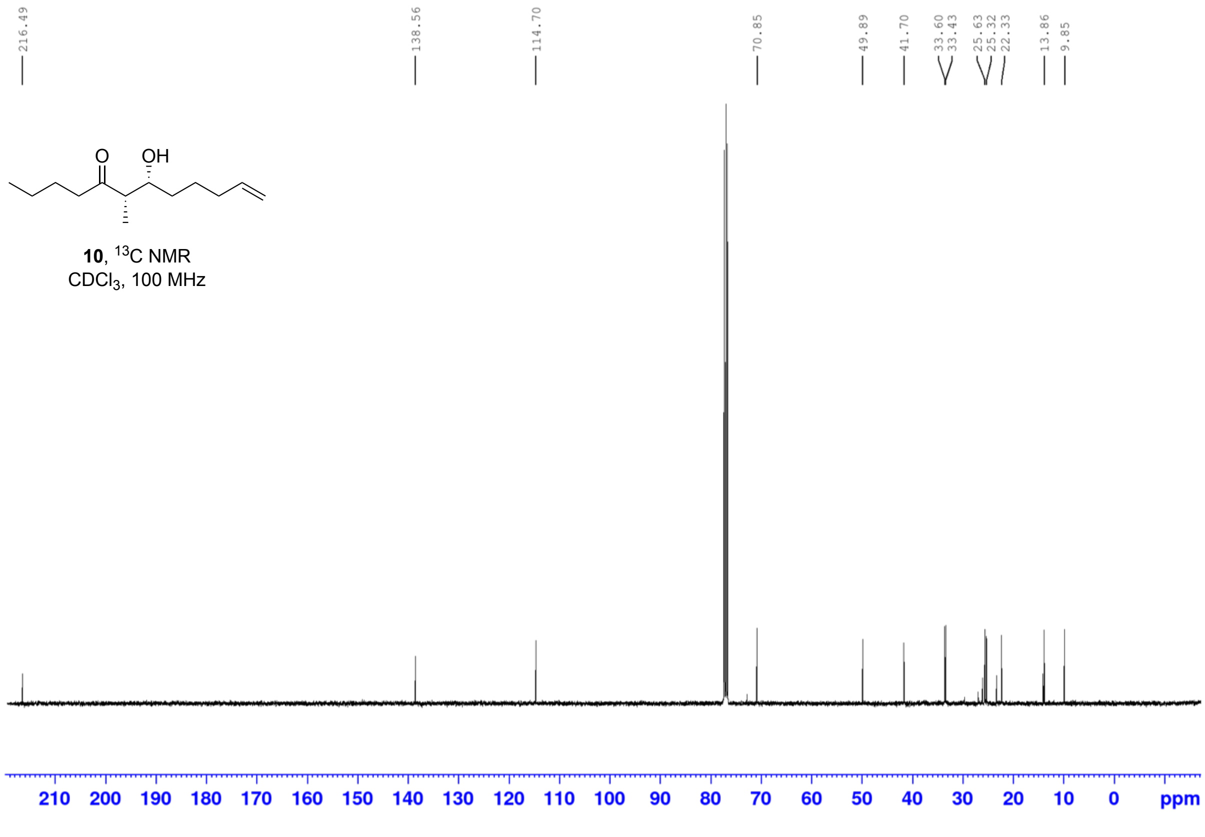
216.49 138.56 114.70 70.85 49.89 41.70 33.60 33.43 25.63 25.32 22.33 13.86 9.85
O
OH
10, 13C NMR
CDCl3, 100 MHz
210 200 190 180 170 160 150 140 130 120 110 100 90 80 70 60 50 40 30 20 10 0 ppm

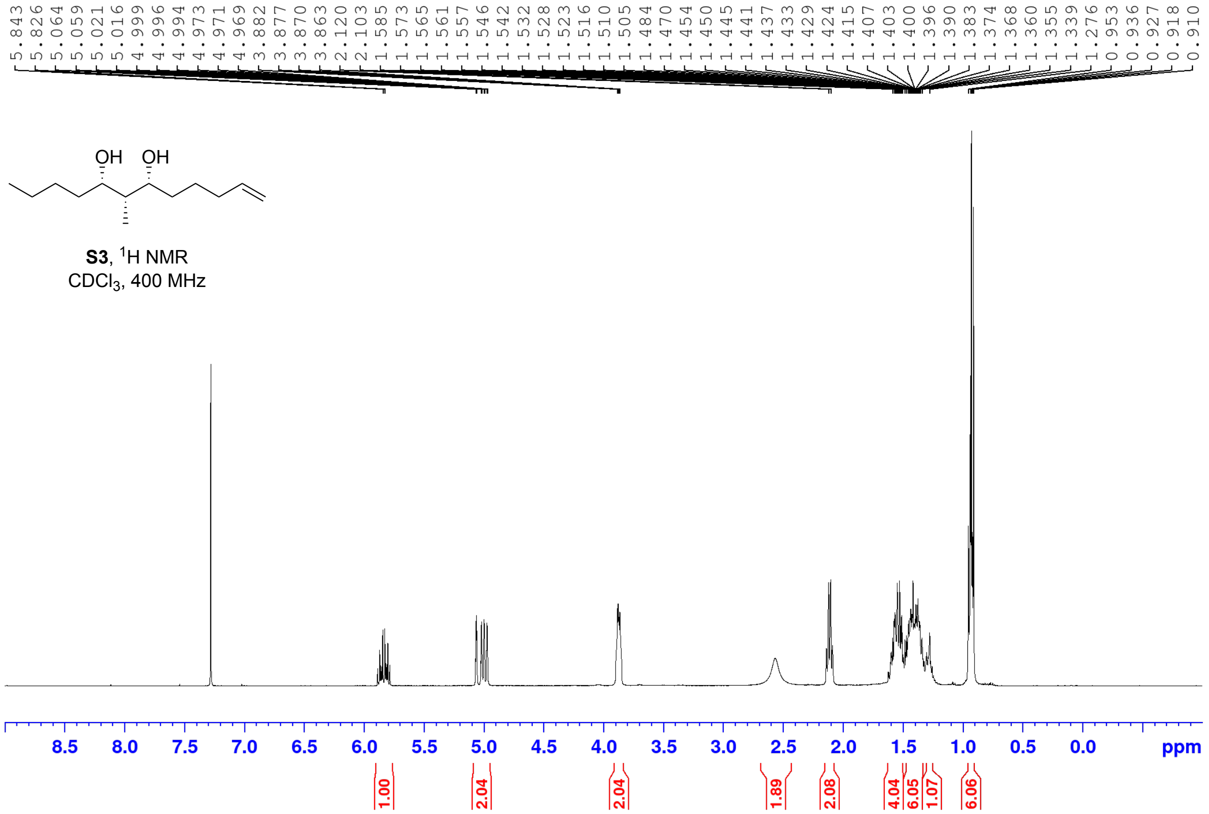
5.843 5.826 5.064 5.059 5.021 5.016 4.999 4.996 4.994 4.973 4.971 4.969 3.882 3.877 3.870 3.863 2.120 2.103 1.585 1.573 1.565 1.561 1.557 1.546 1.542 1.532 1.528 1.523 1.516 1.510 1.505 1.484 1.470 1.454 1.450 1.445 1.441 1.437 1.433 1.429 1.424 1.415 1.407 1.403 1.400 1.396 1.390 1.383 1.374 1.368 1.360 1.355 1.339 1.276 0.953 0.936 0.927 0.918 0.910
OH OH
S3, ^{1}H NMR
$CDCl_3$, 400 MHz
8.5 8.0 7.5 7.0 6.5 6.0 5.5 5.0 4.5 4.0 3.5 3.0 2.5 2.0 1.5 1.0 0.5 0.0 ppm
1.00 2.04 2.04 1.89 2.08 4.04 6.05 1.07 6.06

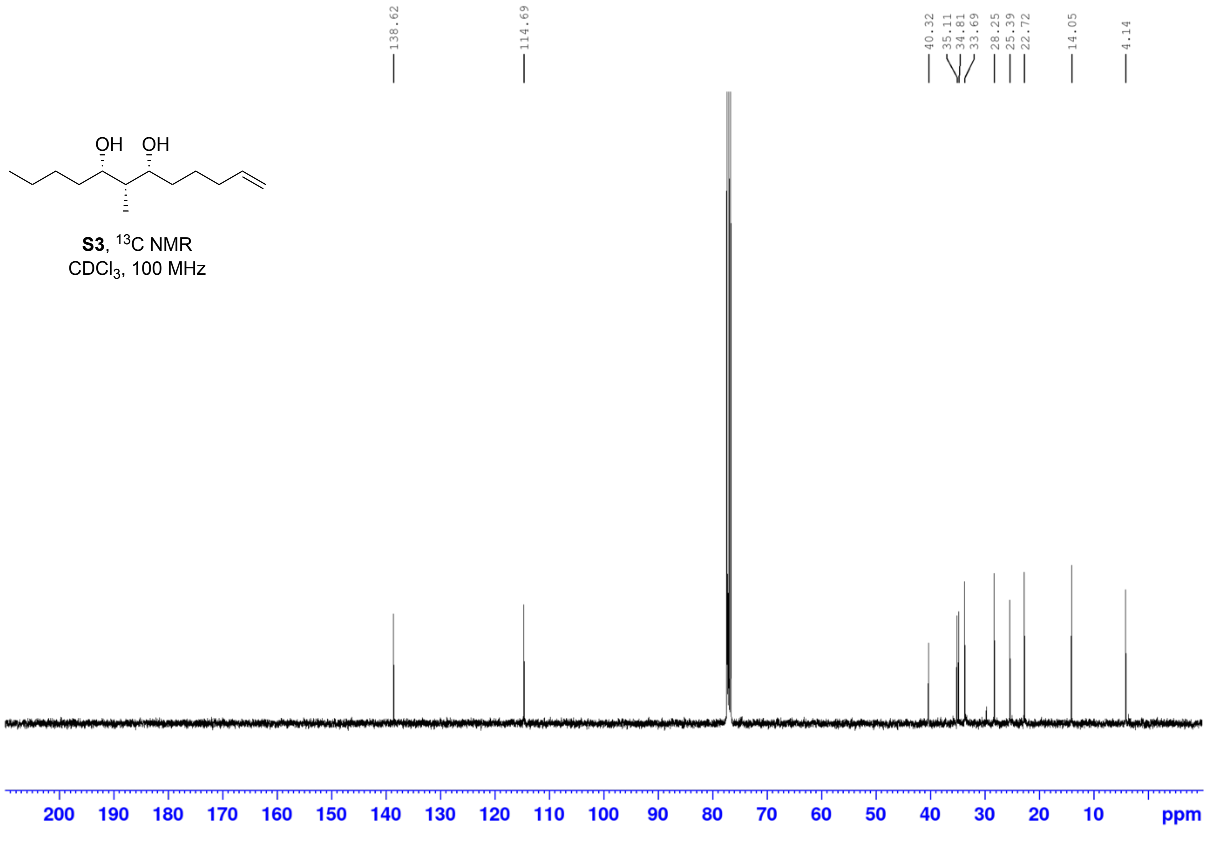
138.62 114.69 40.32 35.11 34.81 33.69 28.25 25.39 22.72 14.05 4.14
OH OH
S3, ^{13}C NMR
$CDCl_3$, 100 MHz
200 190 180 170 160 150 140 130 120 110 100 90 80 70 60 50 40 30 20 10 ppm

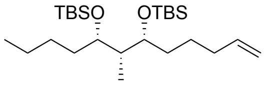

11, ^{1}H NMR
$CDCl_3$, 400 MHz

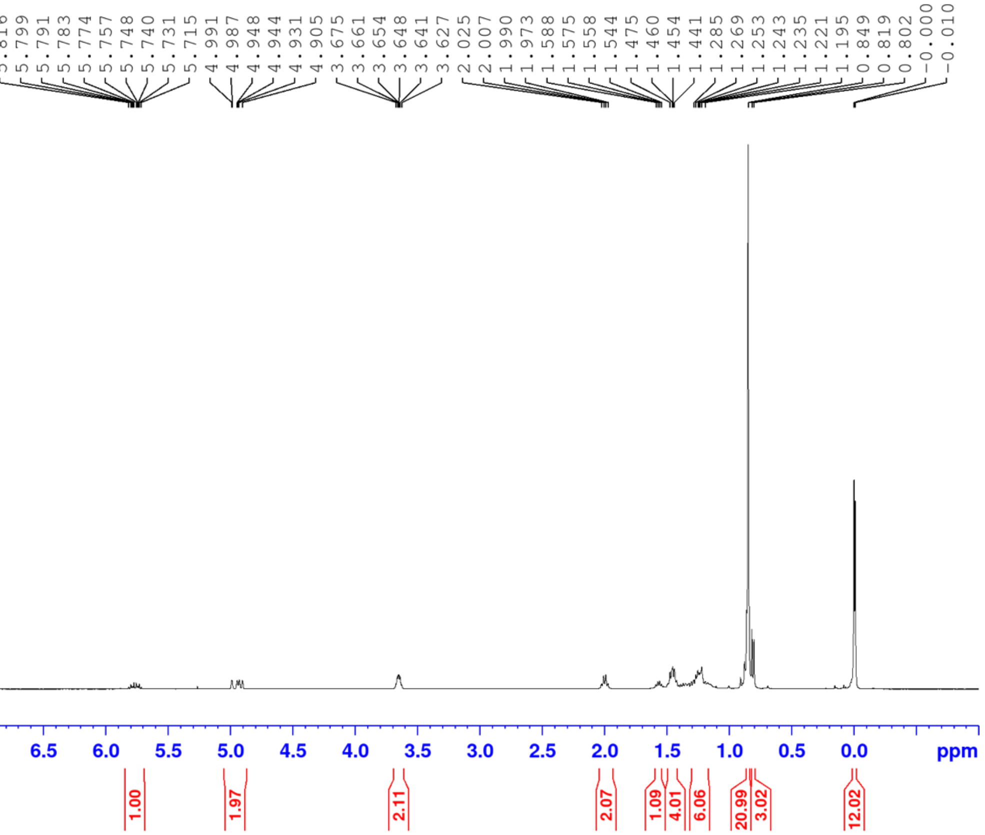

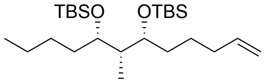

11, ^{13}C NMR
$CDCl_3$, 100 MHz

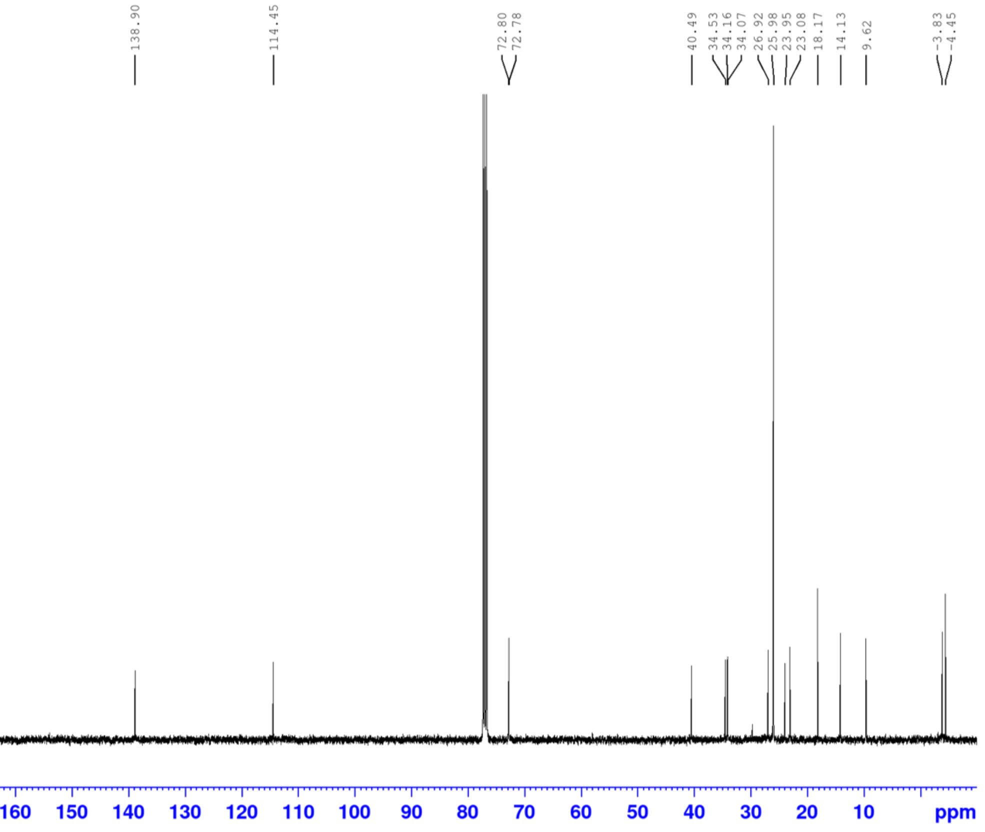

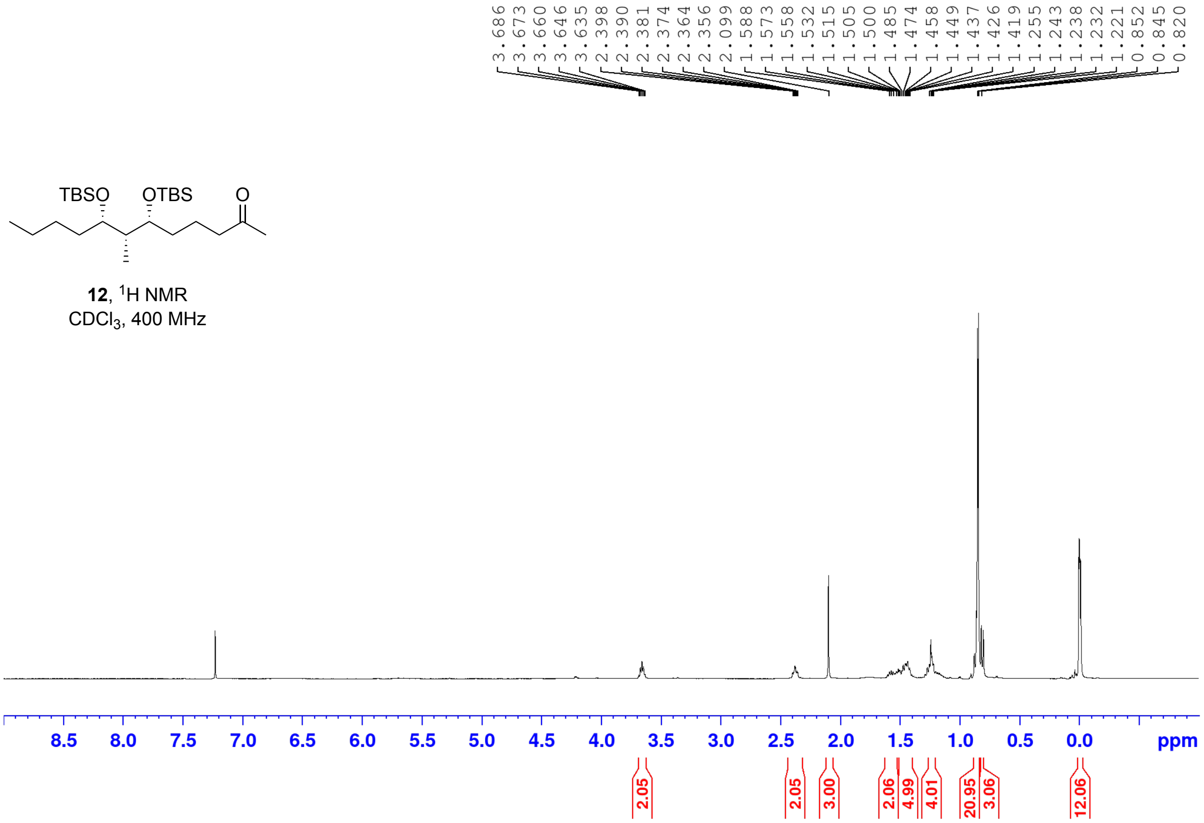
3.686 3.673 3.660 3.646 3.635 2.398 2.390 2.381 2.374 2.364 2.356 2.099 1.588 1.573 1.558 1.532 1.515 1.505 1.500 1.485 1.474 1.458 1.449 1.437 1.426 1.419 1.255 1.243 1.238 1.232 1.221 0.852 0.845 0.820
TBSO OTBS O
12, ^{1}H NMR
$CDCl_3$, 400 MHz
8.5 8.0 7.5 7.0 6.5 6.0 5.5 5.0 4.5 4.0 3.5 3.0 2.5 2.0 1.5 1.0 0.5 0.0 ppm
2.05 2.05 3.00 2.06 4.99 4.01 20.95 3.06 12.06

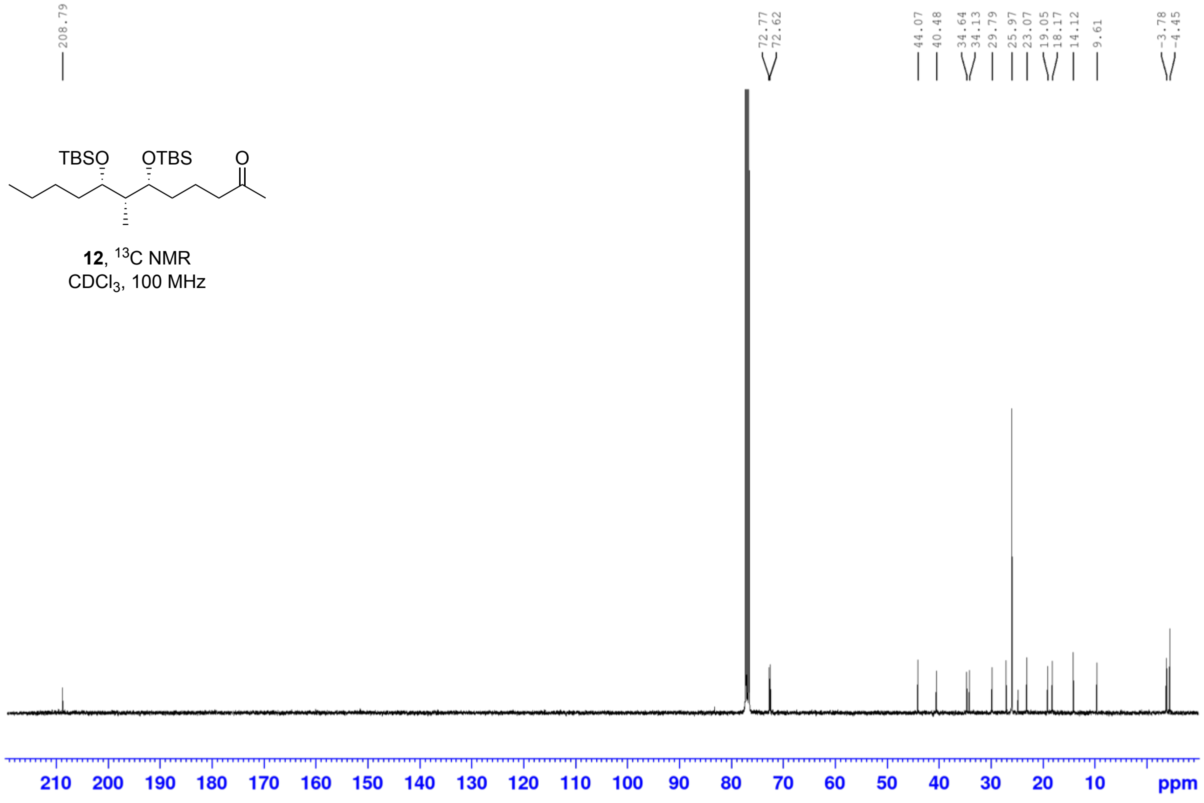
208.79
72.77 72.62
44.07 40.48 34.64 34.13 29.79 25.97 23.07 19.05 18.17 14.12 9.61
-3.78 -4.45
TBSO OTBS O
12, ^{13}C NMR
$CDCl_3$, 100 MHz
210 200 190 180 170 160 150 140 130 120 110 100 90 80 70 60 50 40 30 20 10 ppm

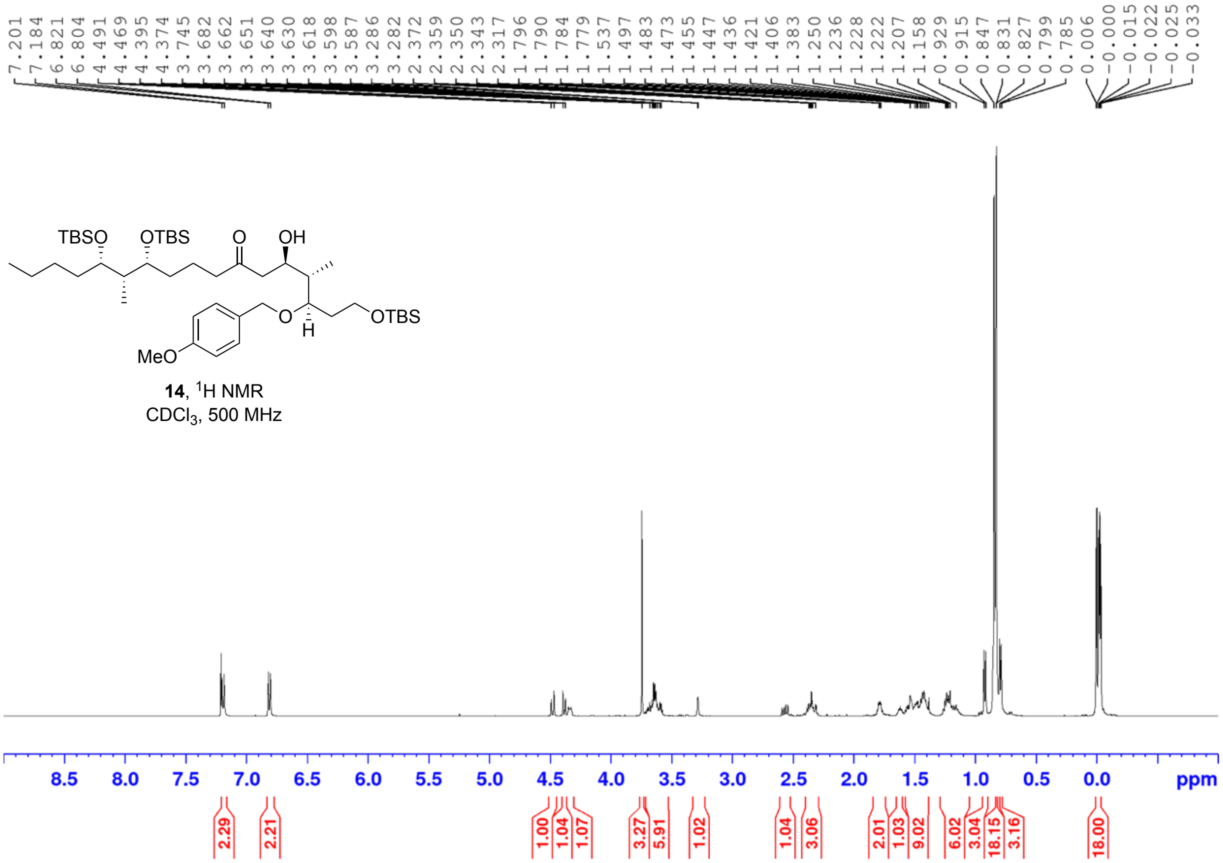

14, ^{1}H NMR
$CDCl_3$, 500 MHz

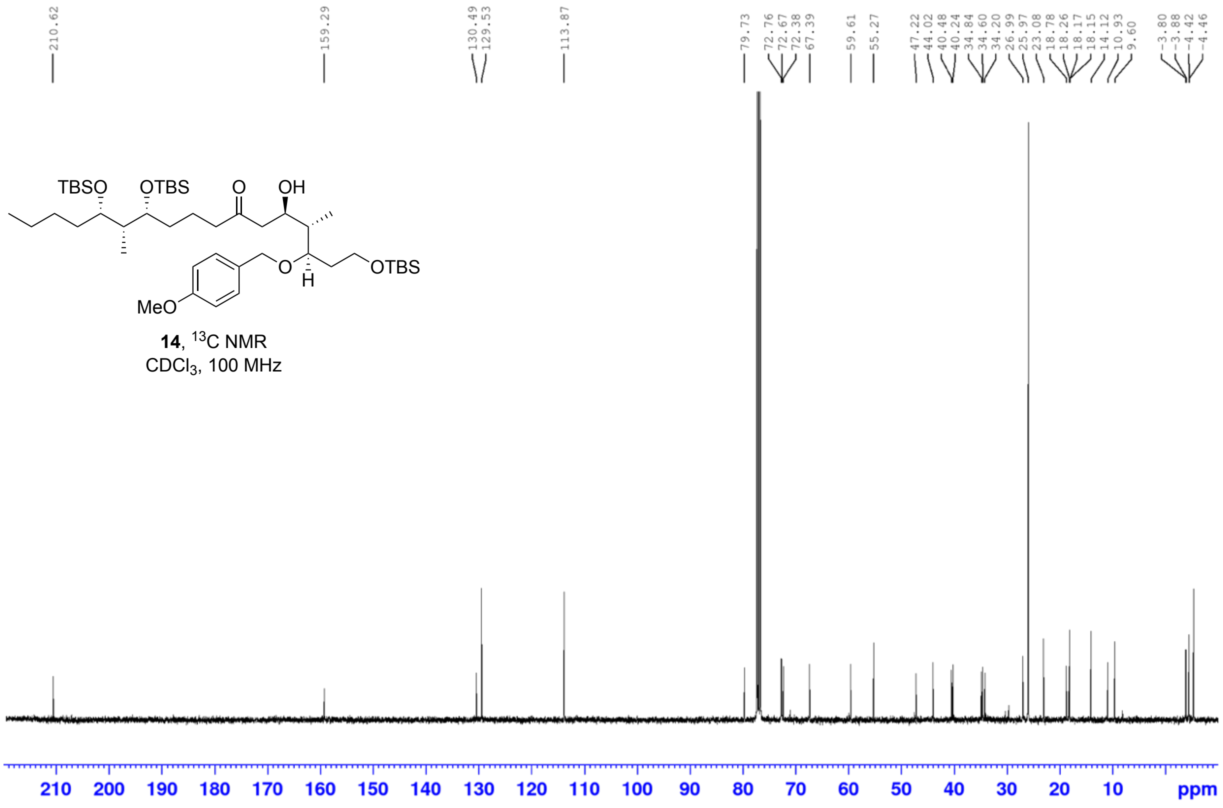

14, ^{13}C NMR
$CDCl_3$, 100 MHz

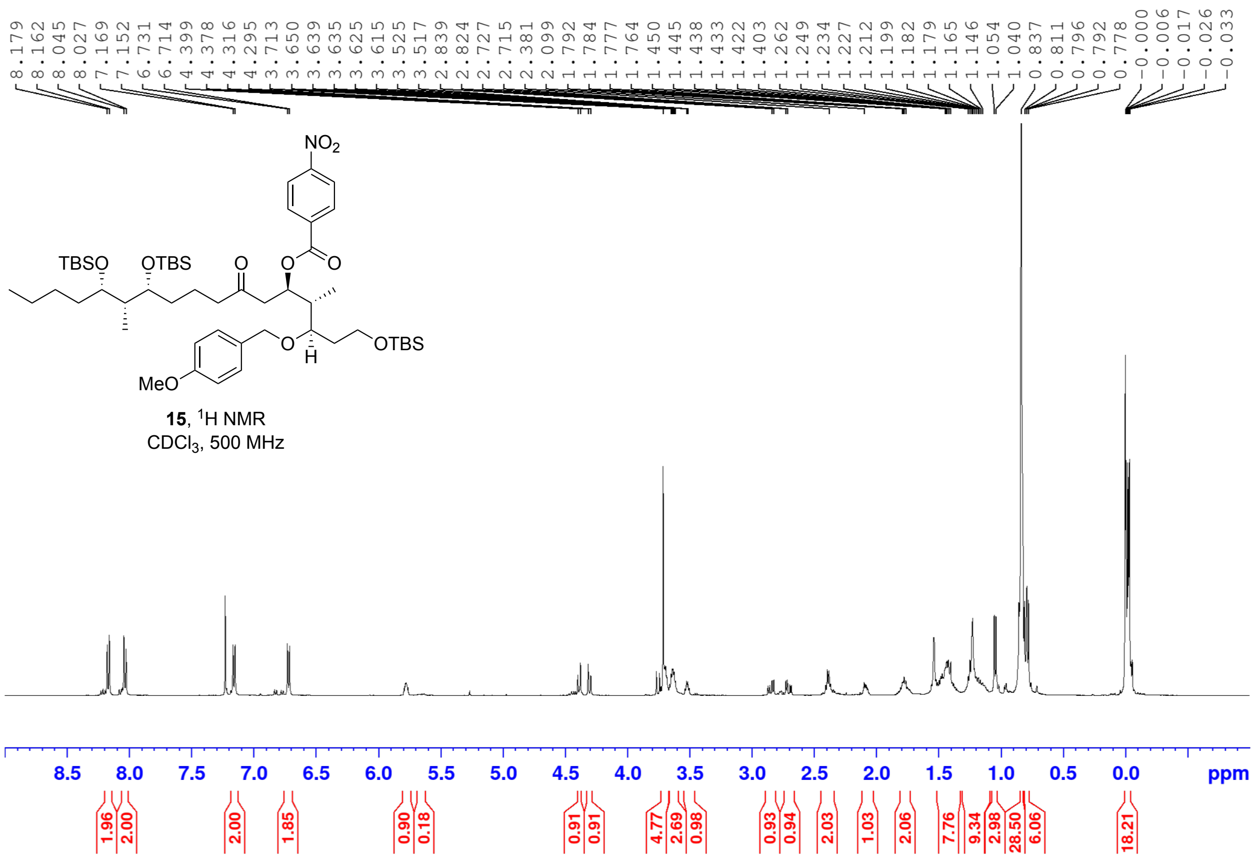

15, ^1H NMR
$CDCl_3$, 500 MHz

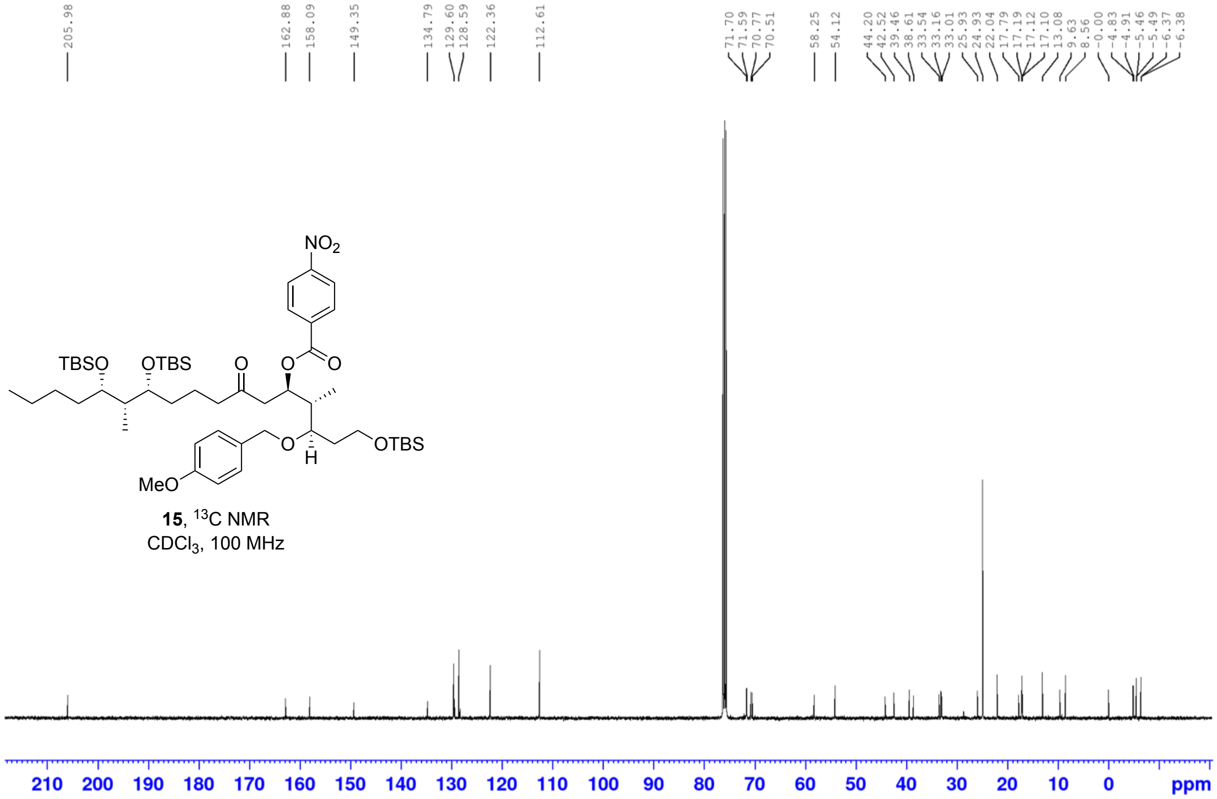

15, ^{13}C NMR
$CDCl_3$, 100 MHz

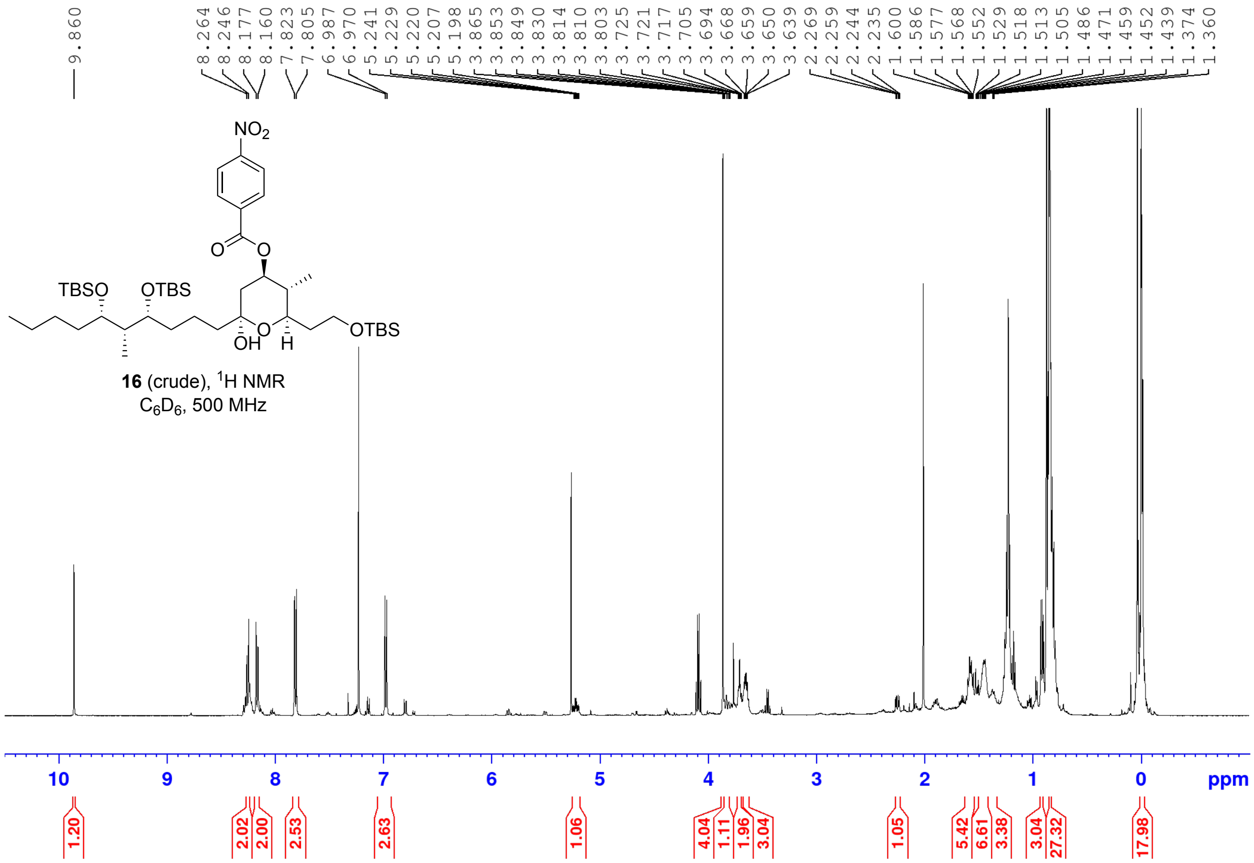
9.860
8.264
8.246
8.177
8.160
7.823
7.805
6.987
6.970
5.241
5.229
5.220
5.207
5.198
3.865
3.853
3.849
3.830
3.814
3.810
3.803
3.725
3.721
3.717
3.705
3.694
3.668
3.659
3.650
3.639
2.269
2.259
2.244
2.235
1.600
1.586
1.577
1.568
1.552
1.529
1.518
1.513
1.505
1.486
1.471
1.459
1.452
1.439
1.374
1.360
NO_2
O
O
TBSO
OTBS
OH
O
H
OTBS
16 (crude), ^{1}H NMR
C_6D_6, 500 MHz
10
9
8
7
6
5
4
3
2
1
0
ppm
1.20
2.02
2.00
2.53
2.63
1.06
4.04
1.11
1.96
3.04
1.05
5.42
6.61
3.38
3.04
27.32
17.98

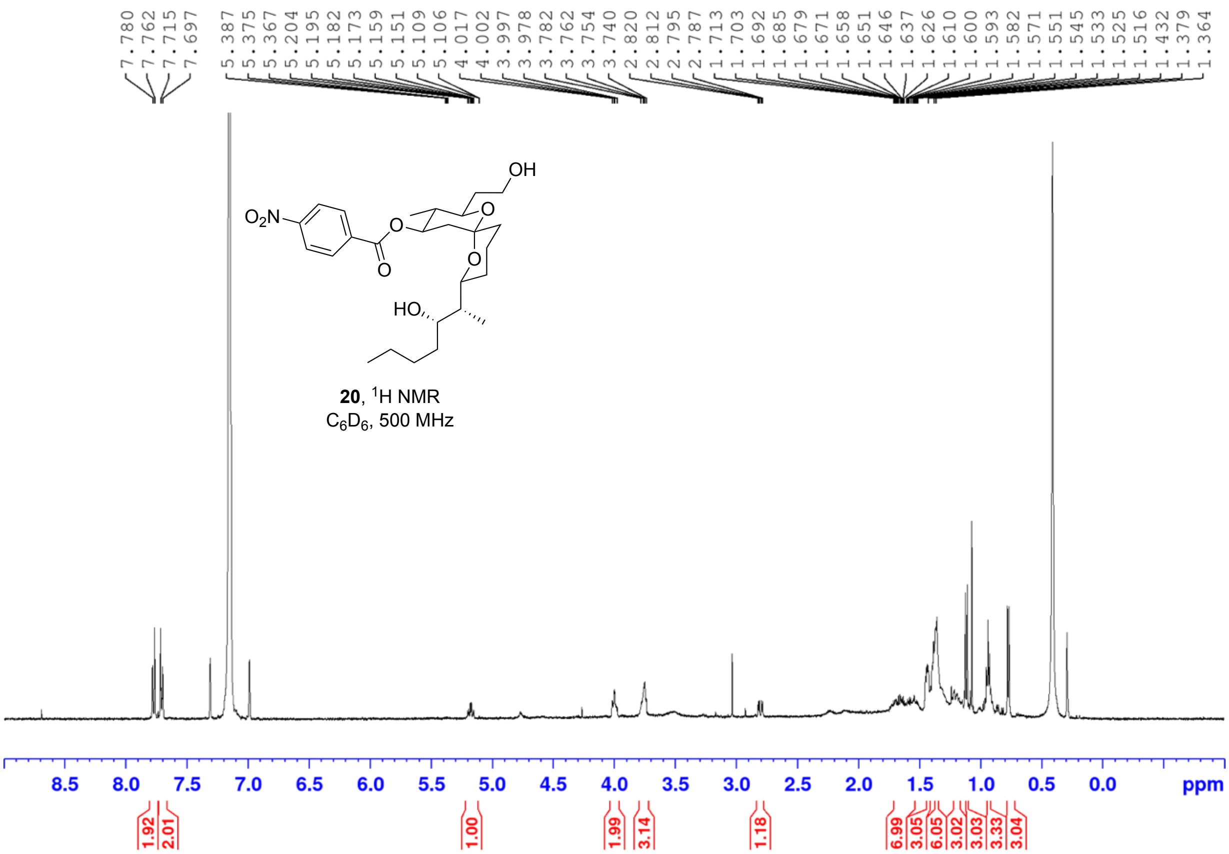
7.780
7.762
7.715
7.697
5.387
5.375
5.367
5.204
5.195
5.182
5.173
5.159
5.151
5.109
5.106
4.017
4.002
3.997
3.978
3.782
3.762
3.754
3.740
2.820
2.812
2.795
2.787
1.713
1.703
1.692
1.685
1.679
1.671
1.658
1.651
1.646
1.637
1.626
1.610
1.600
1.593
1.582
1.571
1.551
1.545
1.533
1.525
1.516
1.432
1.379
1.364
O_2N
O
O
O
O
OH
HO
20, ^{1}H NMR
C_6D_6, 500 MHz
8.5 8.0 7.5 7.0 6.5 6.0 5.5 5.0 4.5 4.0 3.5 3.0 2.5 2.0 1.5 1.0 0.5 0.0 ppm
1.92
2.01
1.00
1.99
3.14
1.18
6.99
3.05
6.05
3.02
3.03
3.33
3.04

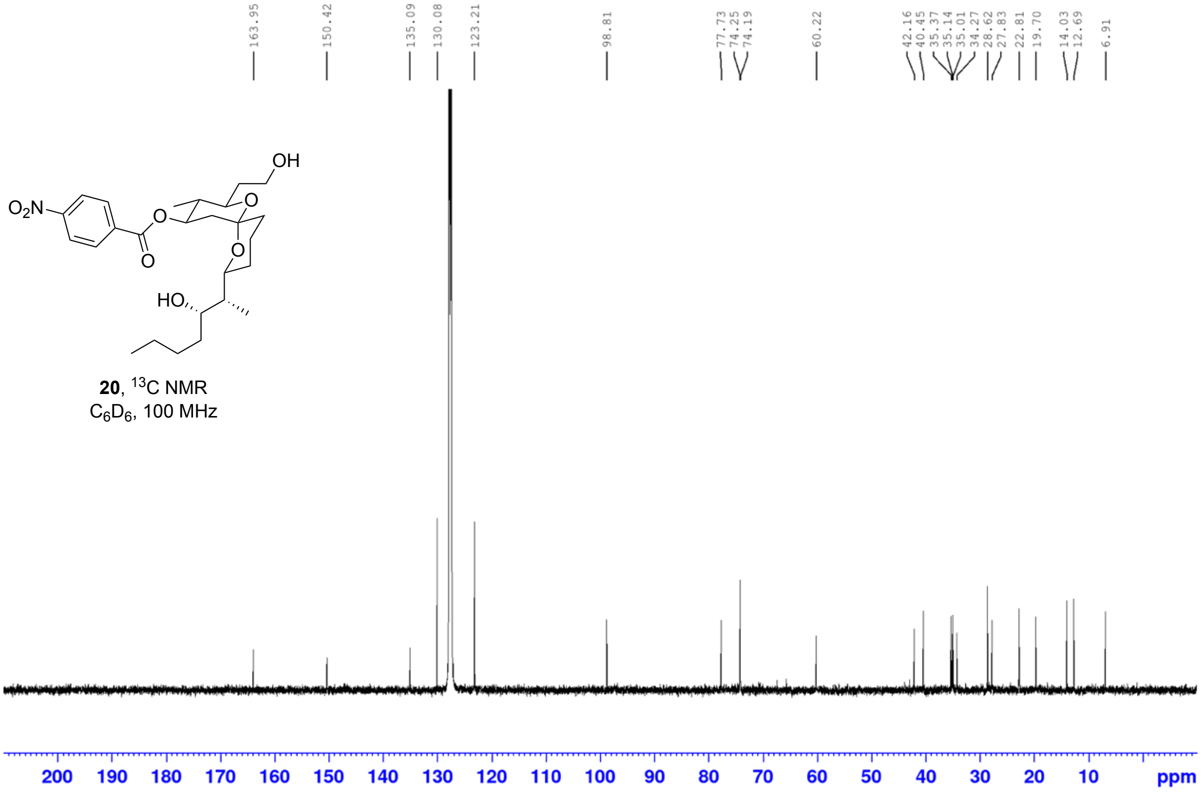
163.95
150.42
135.09
130.08
123.21
98.81
77.73
74.25
74.19
60.22
42.16
40.45
35.37
35.14
35.01
34.27
28.62
27.83
22.81
19.70
14.03
12.69
6.91
O_2N
O
O
O
O
OH
HO
20, ^{13}C NMR
C_6D_6, 100 MHz
200 190 180 170 160 150 140 130 120 110 100 90 80 70 60 50 40 30 20 10 ppm

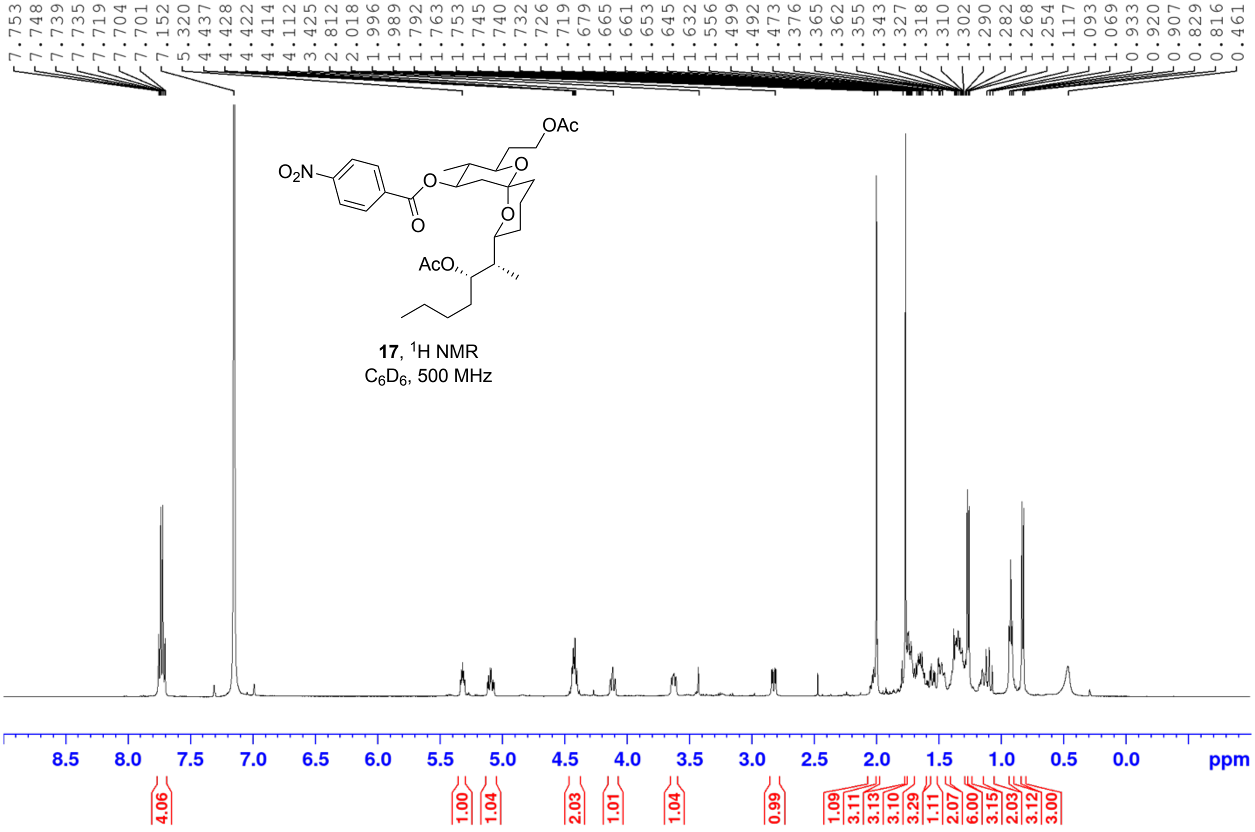
7.753 7.748 7.739 7.735 7.719 7.704 7.701 7.152 5.320 4.437 4.428 4.422 4.414 4.112 3.425 2.812 2.018 1.996 1.989 1.792 1.763 1.753 1.745 1.740 1.732 1.726 1.719 1.679 1.665 1.661 1.653 1.645 1.632 1.556 1.499 1.492 1.473 1.376 1.365 1.362 1.355 1.343 1.327 1.318 1.310 1.302 1.290 1.282 1.268 1.254 1.117 1.093 1.069 0.933 0.920 0.907 0.829 0.816 0.461
OAc
O2N
O
AcO
17, 1H NMR
C6D6, 500 MHz
8.5 8.0 7.5 7.0 6.5 6.0 5.5 5.0 4.5 4.0 3.5 3.0 2.5 2.0 1.5 1.0 0.5 0.0 ppm
4.06 1.00 1.04 2.03 1.01 1.04 0.99 1.09 3.11 3.13 3.10 3.29 1.11 2.07 6.00 3.15 2.03 3.12 3.00

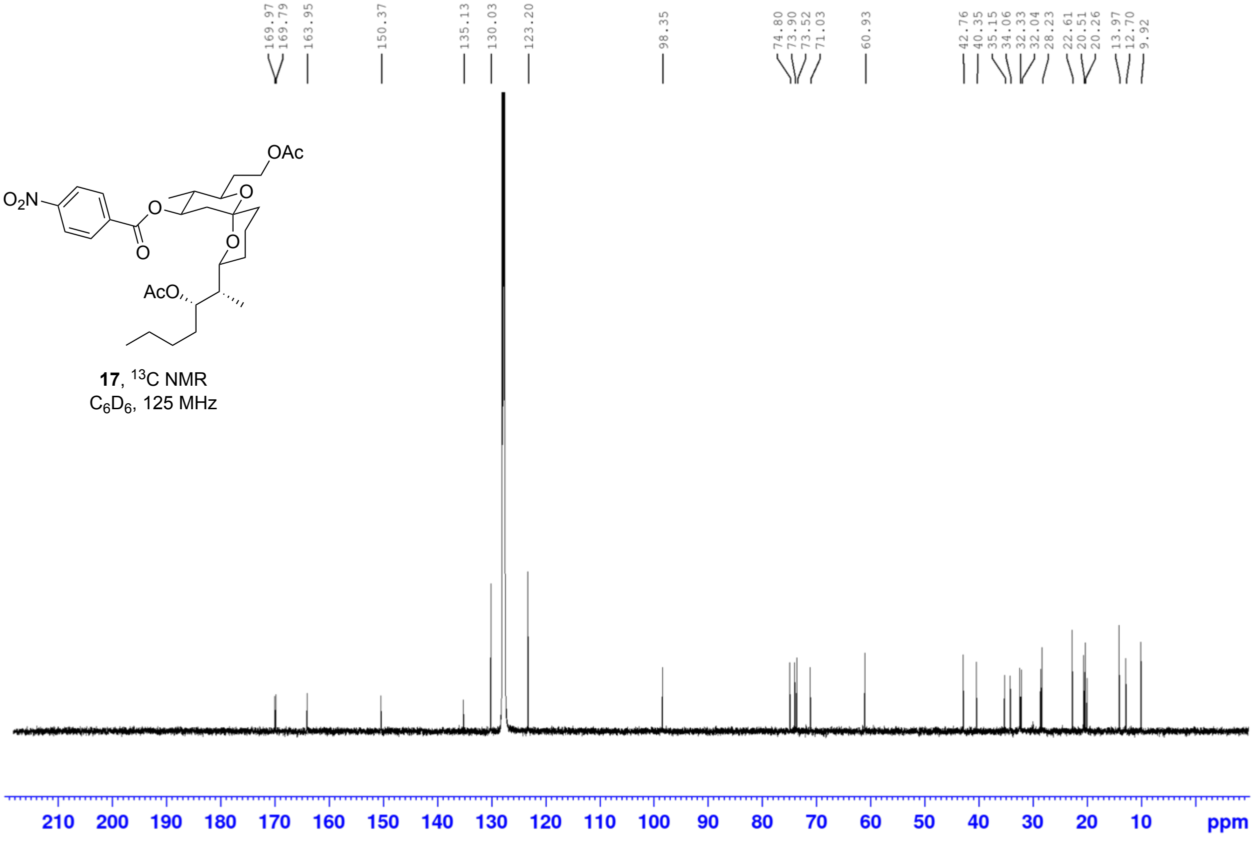
169.97 169.79 163.95 150.37 135.13 130.03 123.20 98.35 74.80 73.90 73.52 71.03 60.93 42.76 40.35 35.15 34.06 32.33 32.04 28.23 22.61 20.51 20.26 13.97 12.70 9.92
OAc
O2N
O
AcO
17, 13C NMR
C6D6, 125 MHz
210 200 190 180 170 160 150 140 130 120 110 100 90 80 70 60 50 40 30 20 10 ppm

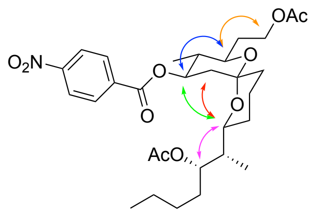

17, NOESY (top), COSY (bottom)
C_6D_6, 500 MHz

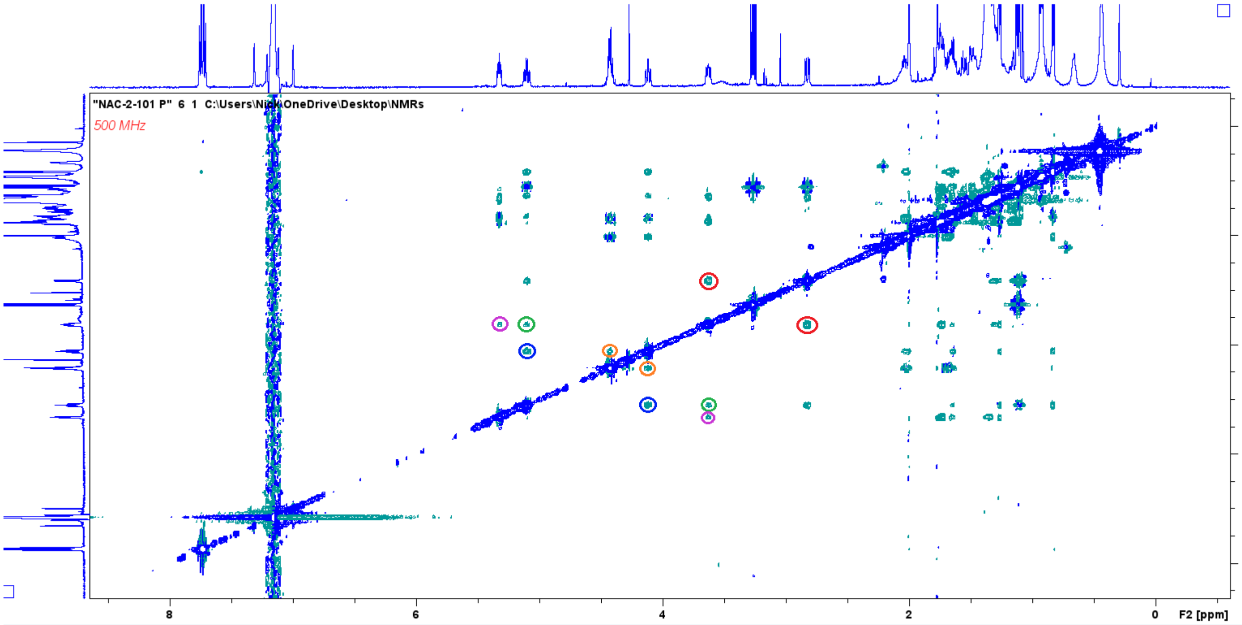

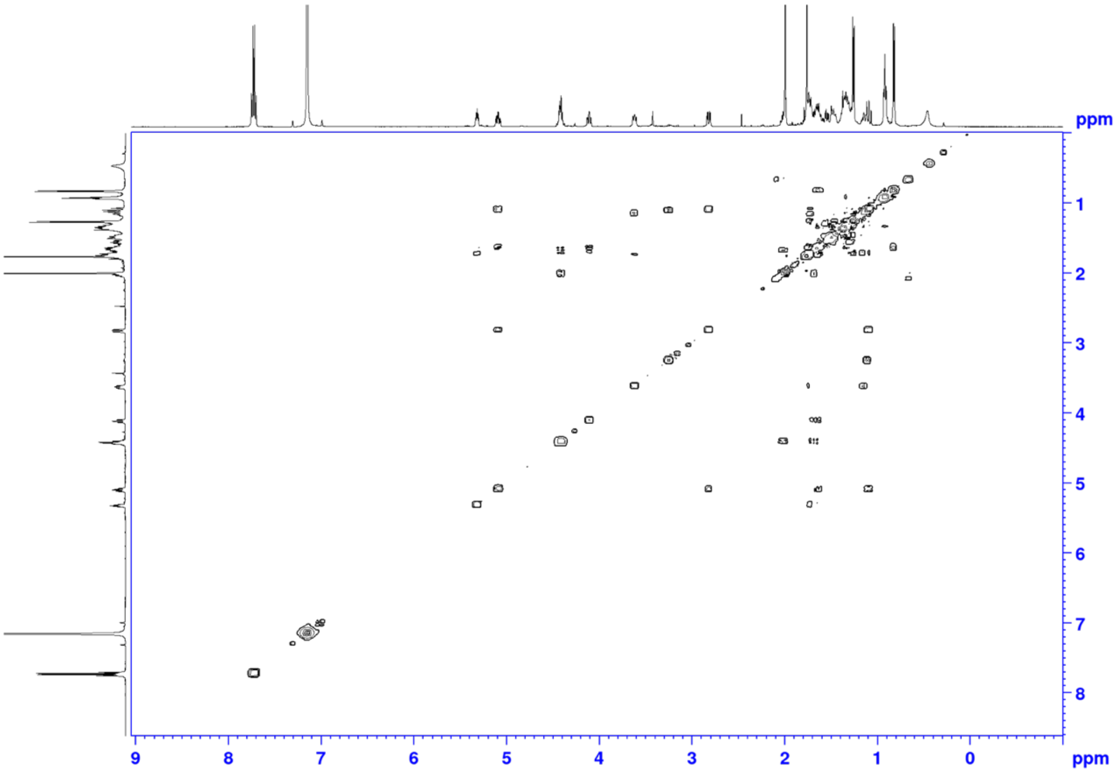

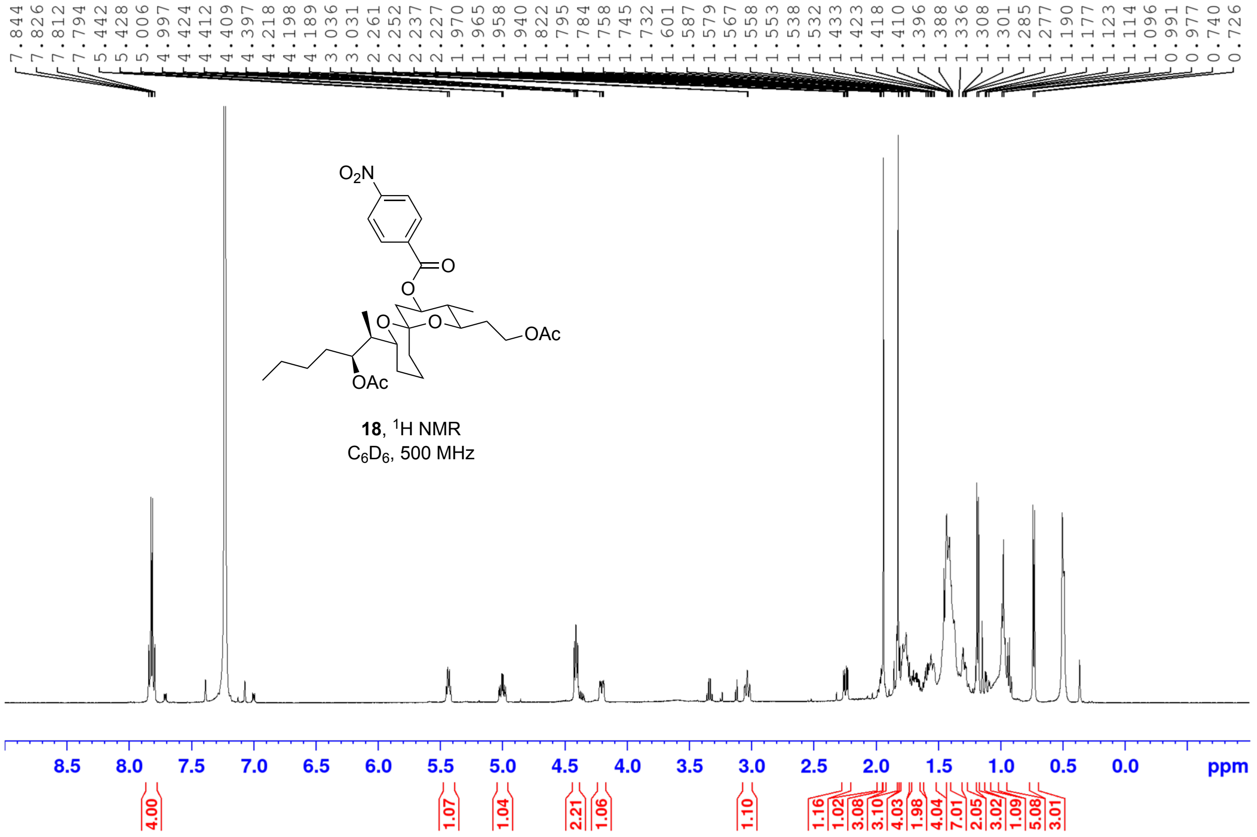
O2N
O
O
O
O
OAc
OAc
18, 1H NMR
C6D6, 500 MHz
8.5 8.0 7.5 7.0 6.5 6.0 5.5 5.0 4.5 4.0 3.5 3.0 2.5 2.0 1.5 1.0 0.5 0.0 ppm
4.00 1.07 1.04 2.21 1.06 1.10 1.16 1.02 3.08 3.10 4.03 1.98 4.04 7.01 2.05 3.02 1.09 5.08 3.01

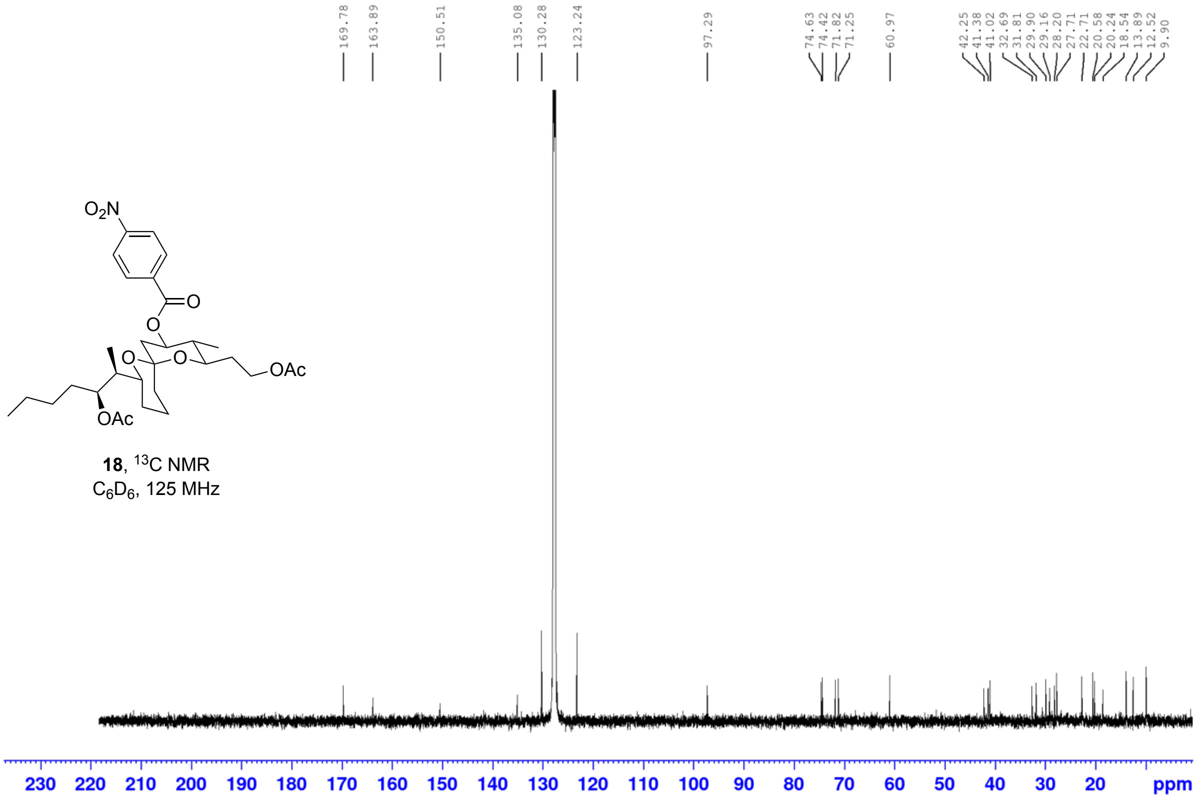
169.78
163.89
150.51
135.08
130.28
123.24
97.29
74.63
74.42
71.82
71.25
60.97
42.25
41.38
41.02
32.69
31.81
29.90
29.16
28.20
27.71
22.71
20.58
20.24
18.54
13.89
12.52
9.90
O2N
O
O
O
O
OAc
OAc
18, 13C NMR
C6D6, 125 MHz
230 220 210 200 190 180 170 160 150 140 130 120 110 100 90 80 70 60 50 40 30 20 ppm

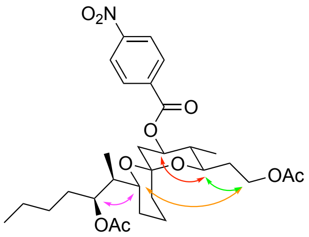

18, NOESY
C_6D_6, 500 MHz

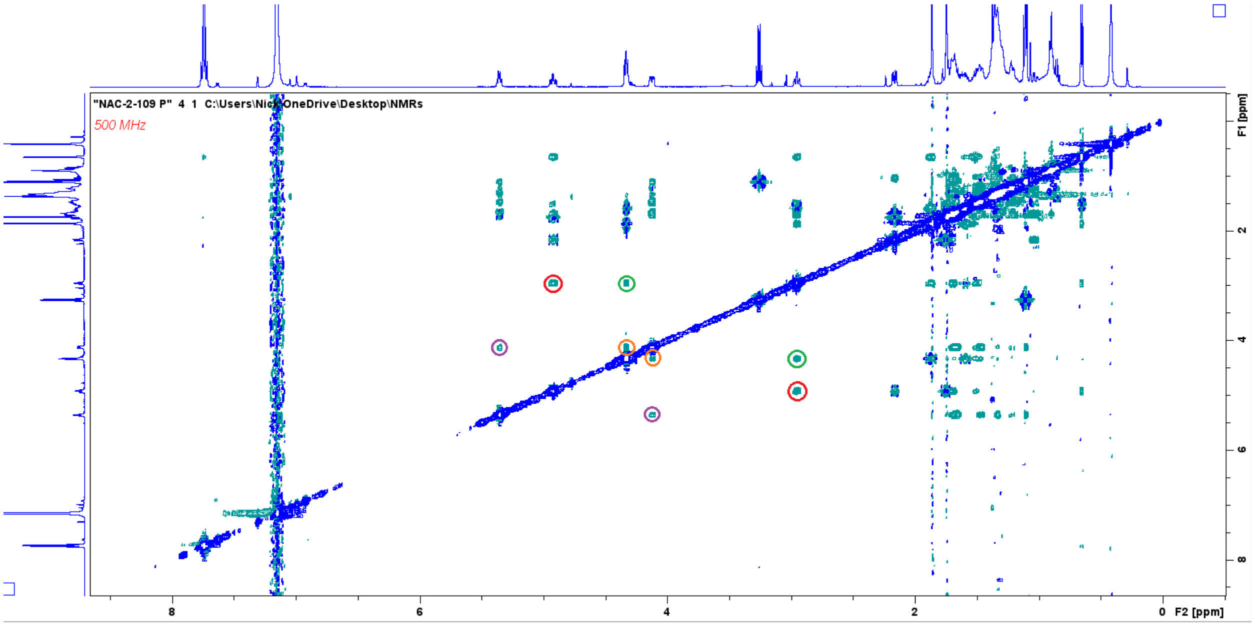

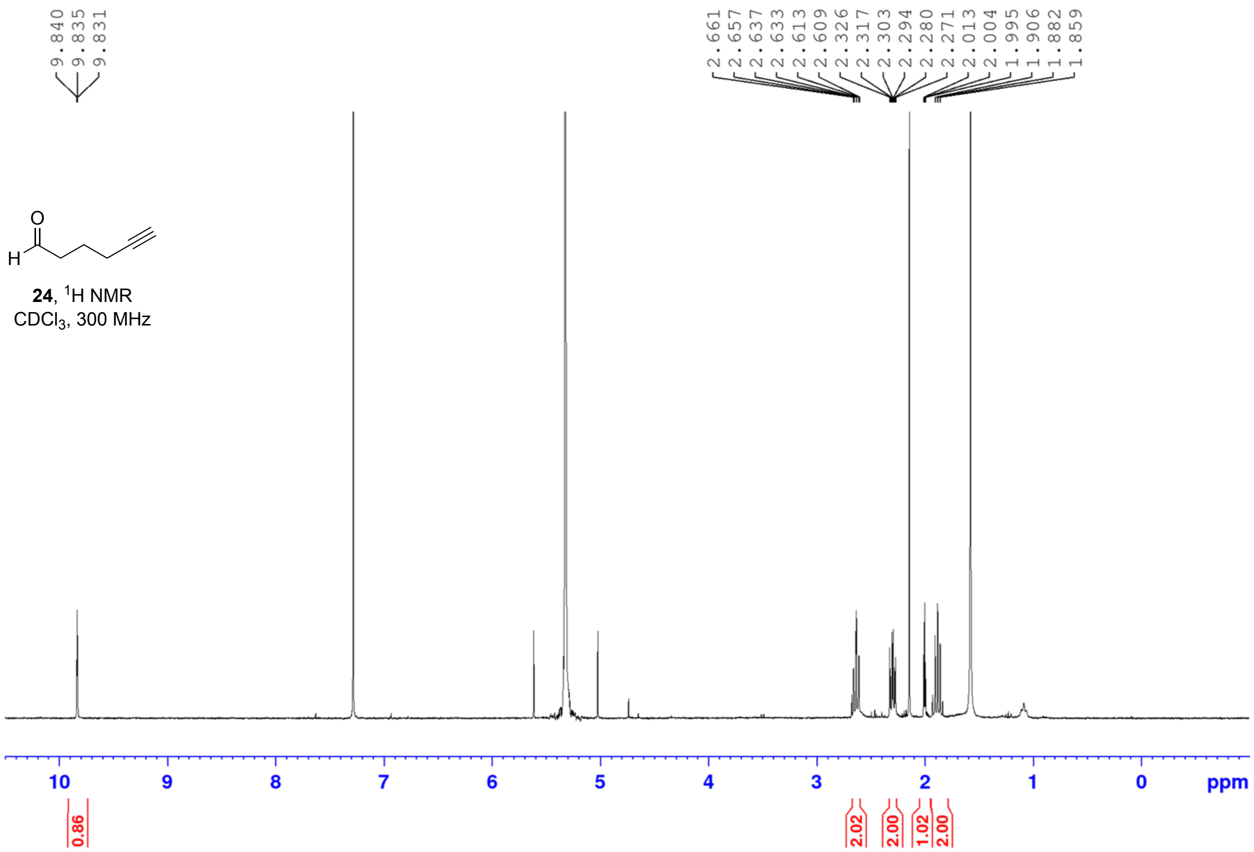
9.840
9.835
9.831
2.661
2.657
2.637
2.633
2.613
2.609
2.326
2.317
2.303
2.294
2.280
2.271
2.013
2.004
1.995
1.906
1.882
1.859
O
H
24, ^{1}H NMR
$CDCl_3$, 300 MHz
10
9
8
7
6
5
4
3
2
1
0
ppm
0.86
2.02
2.00
1.02
2.00

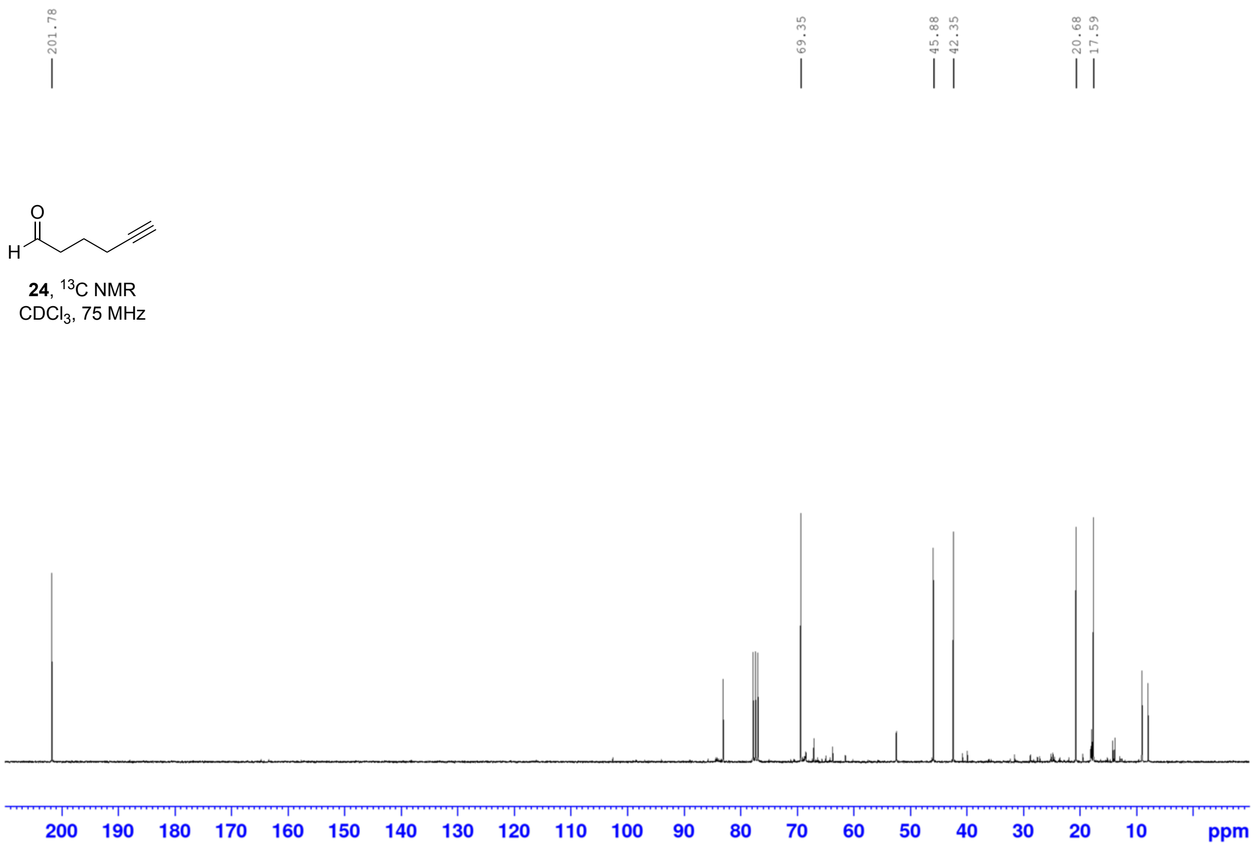
201.78
69.35
45.88
42.35
20.68
17.59
O
H
24, ^{13}C NMR
$CDCl_3$, 75 MHz
200
190
180
170
160
150
140
130
120
110
100
90
80
70
60
50
40
30
20
10
ppm

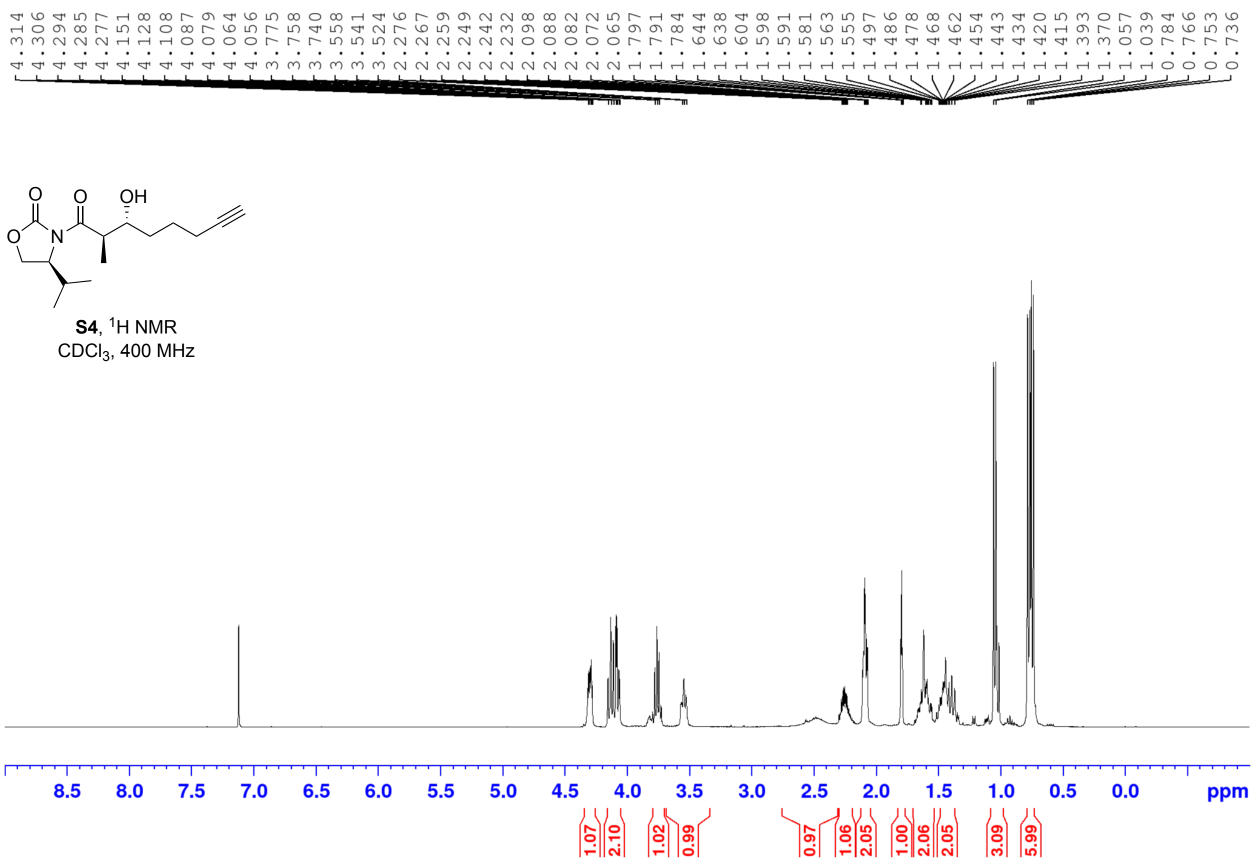

4.314 4.306 4.294 4.285 4.277 4.151 4.128 4.108 4.087 4.079 4.064 4.056 3.775 3.758 3.740 3.558 3.541 3.524 2.276 2.267 2.259 2.249 2.242 2.232 2.098 2.088 2.082 2.072 2.065 1.797 1.791 1.784 1.644 1.638 1.604 1.598 1.591 1.581 1.563 1.555 1.497 1.486 1.478 1.468 1.462 1.454 1.443 1.434 1.420 1.415 1.393 1.370 1.057 1.039 0.784 0.766 0.753 0.736
O
O
N
O
OH
S4, 1H NMR
CDCl3, 400 MHz
8.5 8.0 7.5 7.0 6.5 6.0 5.5 5.0 4.5 4.0 3.5 3.0 2.5 2.0 1.5 1.0 0.5 0.0 ppm
1.07 2.10 1.02 0.99 0.97 1.06 2.05 1.00 2.06 2.05 3.09 5.99

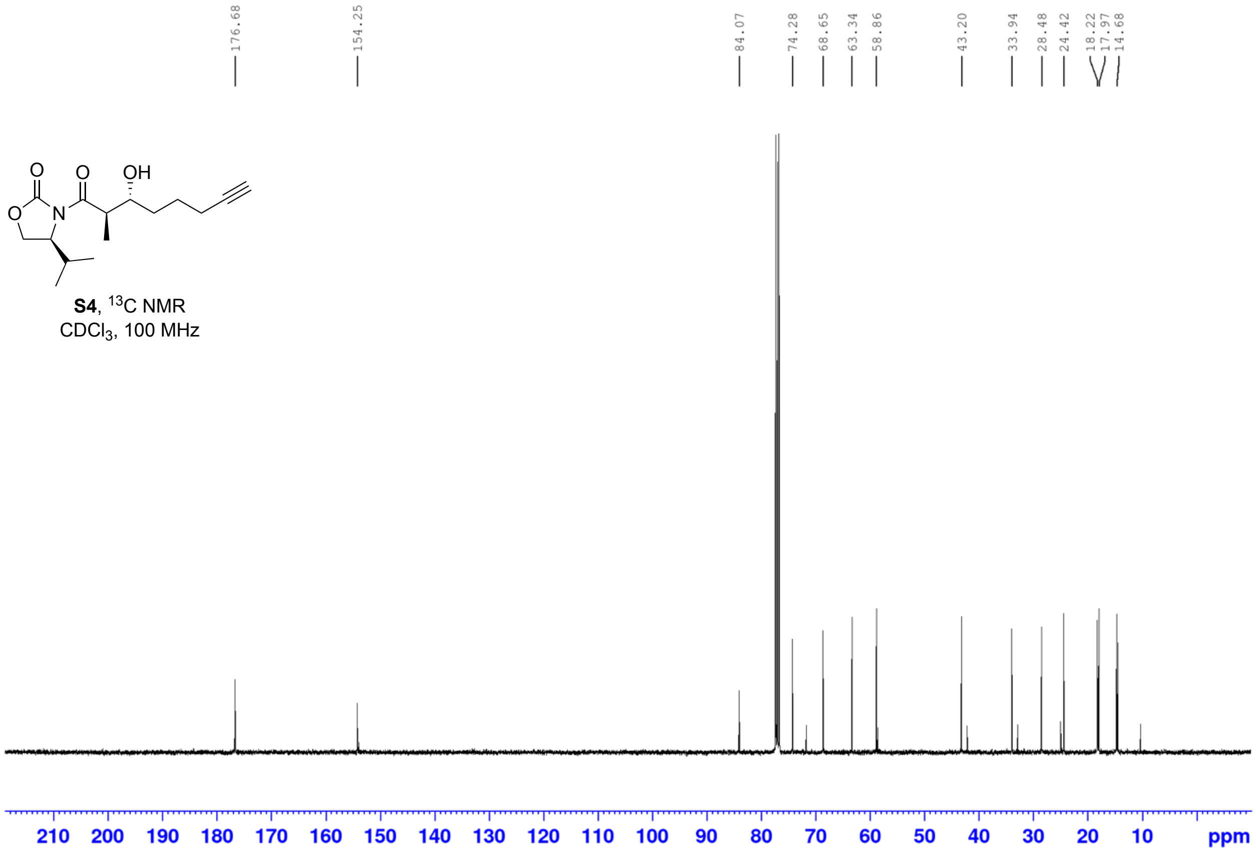

176.68 154.25 84.07 74.28 68.65 63.34 58.86 43.20 33.94 28.48 24.42 18.22 17.97 14.68
O
O
N
O
OH
S4, 13C NMR
CDCl3, 100 MHz
210 200 190 180 170 160 150 140 130 120 110 100 90 80 70 60 50 40 30 20 10 ppm

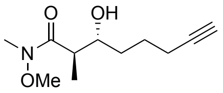

25, not purified, ^{1}H NMR
$CDCl_3$, 400 MHz

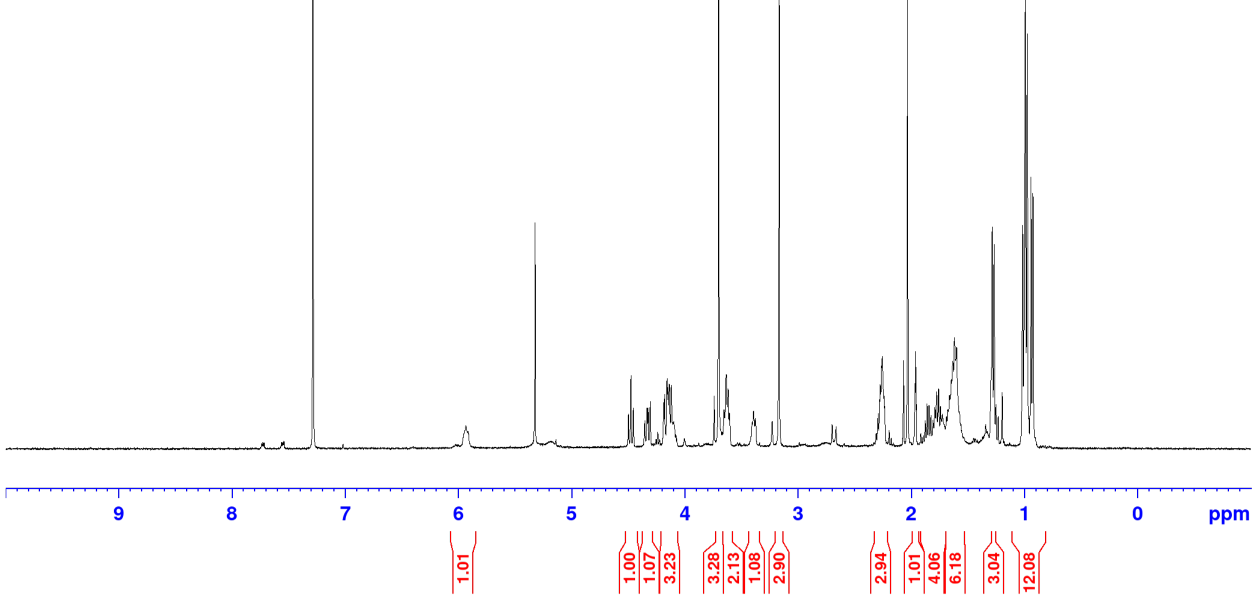

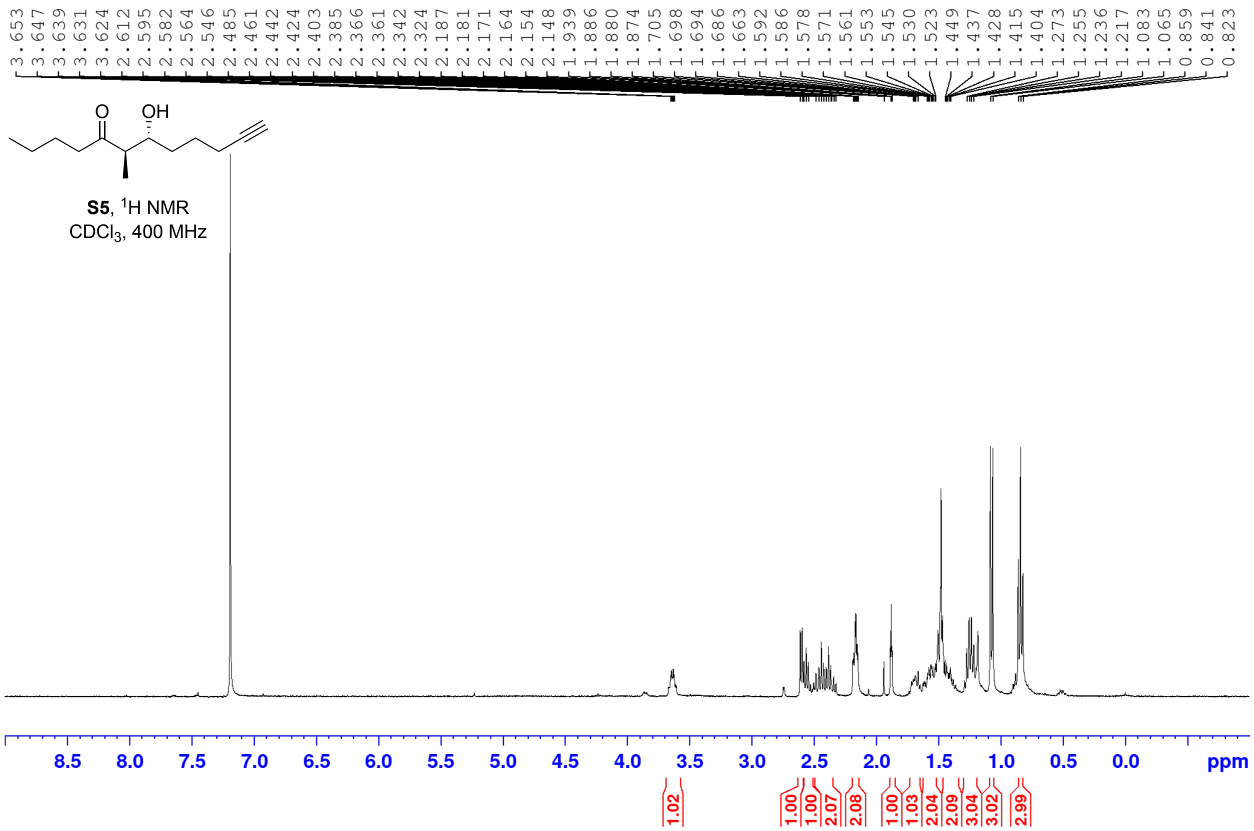
3.653 3.647 3.639 3.631 3.624 3.612 2.595 2.582 2.564 2.546 2.485 2.461 2.442 2.424 2.403 2.385 2.366 2.361 2.342 2.324 2.187 2.181 2.171 2.164 2.154 2.148 1.939 1.886 1.880 1.874 1.705 1.698 1.694 1.686 1.663 1.592 1.586 1.578 1.571 1.561 1.553 1.545 1.530 1.523 1.449 1.437 1.428 1.415 1.404 1.273 1.255 1.236 1.217 1.083 1.065 0.859 0.841 0.823
O
OH
S5, 1H NMR
CDCl3, 400 MHz
8.5 8.0 7.5 7.0 6.5 6.0 5.5 5.0 4.5 4.0 3.5 3.0 2.5 2.0 1.5 1.0 0.5 0.0 ppm
1.02 1.00 1.00 2.07 2.08 1.00 1.03 2.04 2.09 3.04 3.02 2.99

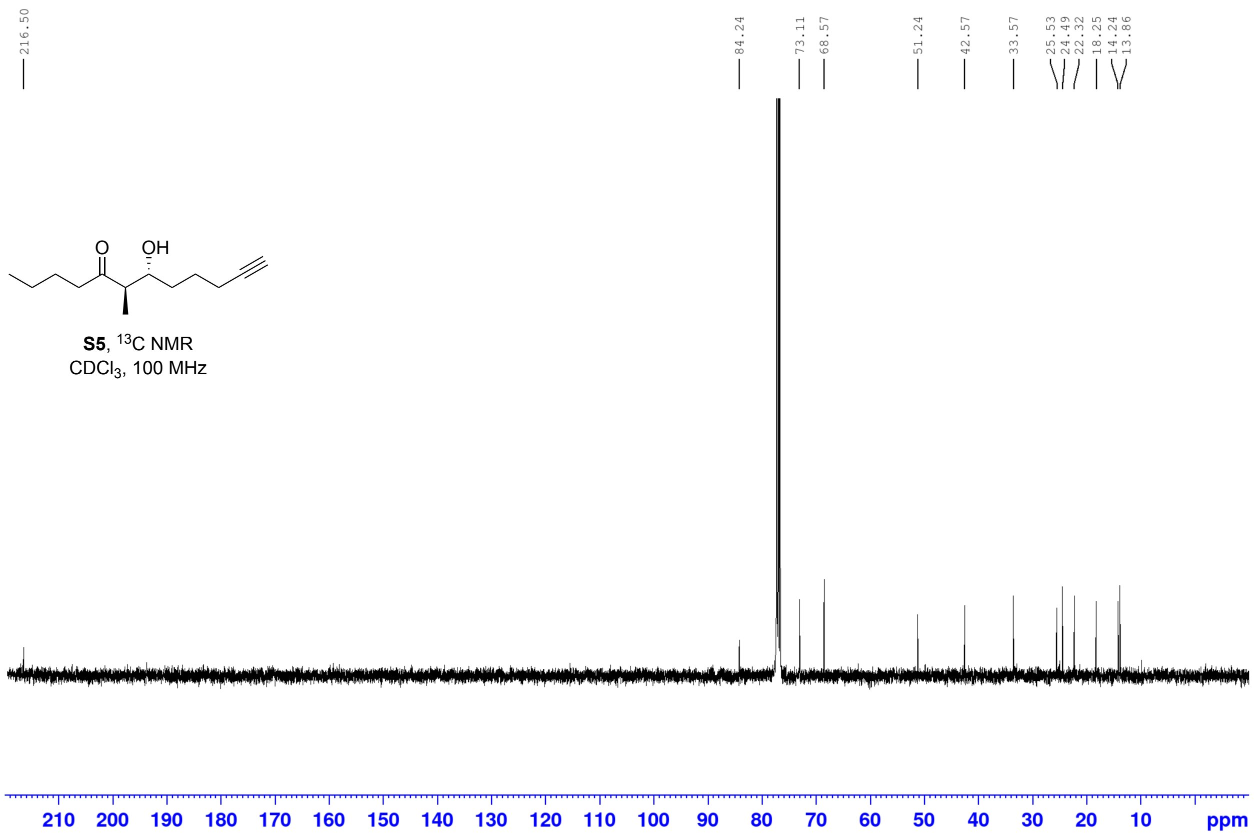
216.50 84.24 73.11 68.57 51.24 42.57 33.57 25.53 24.49 22.32 18.25 14.24 13.86
O
OH
S5, 13C NMR
CDCl3, 100 MHz
210 200 190 180 170 160 150 140 130 120 110 100 90 80 70 60 50 40 30 20 10 ppm

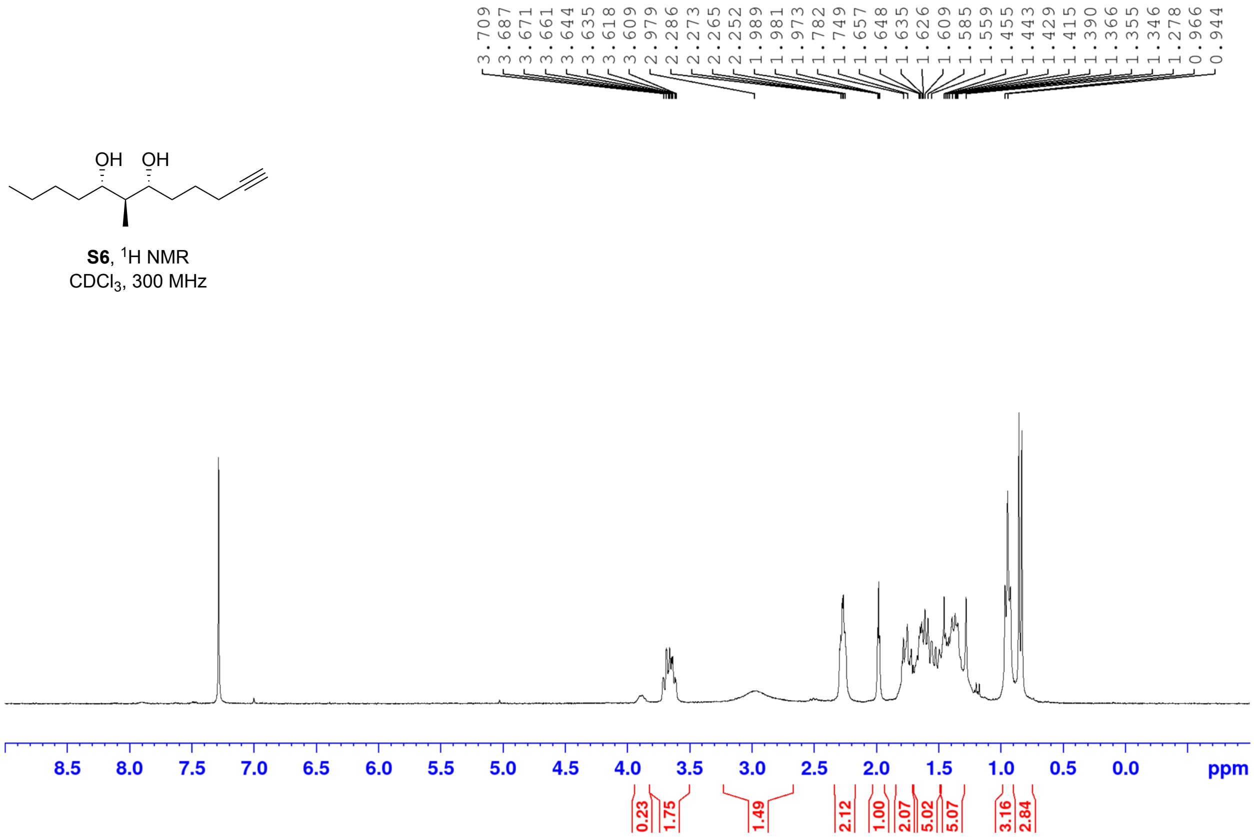

3.709 3.687 3.671 3.661 3.644 3.635 3.618 3.609 2.979 2.286 2.273 2.265 2.252 1.989 1.981 1.973 1.782 1.749 1.657 1.648 1.635 1.626 1.609 1.585 1.559 1.455 1.443 1.429 1.415 1.390 1.366 1.355 1.346 1.278 0.966 0.944
OH OH
S6, 1H NMR
CDCl3, 300 MHz
8.5 8.0 7.5 7.0 6.5 6.0 5.5 5.0 4.5 4.0 3.5 3.0 2.5 2.0 1.5 1.0 0.5 0.0 ppm
0.23 1.75 1.49 2.12 1.00 2.07 5.02 5.07 3.16 2.84

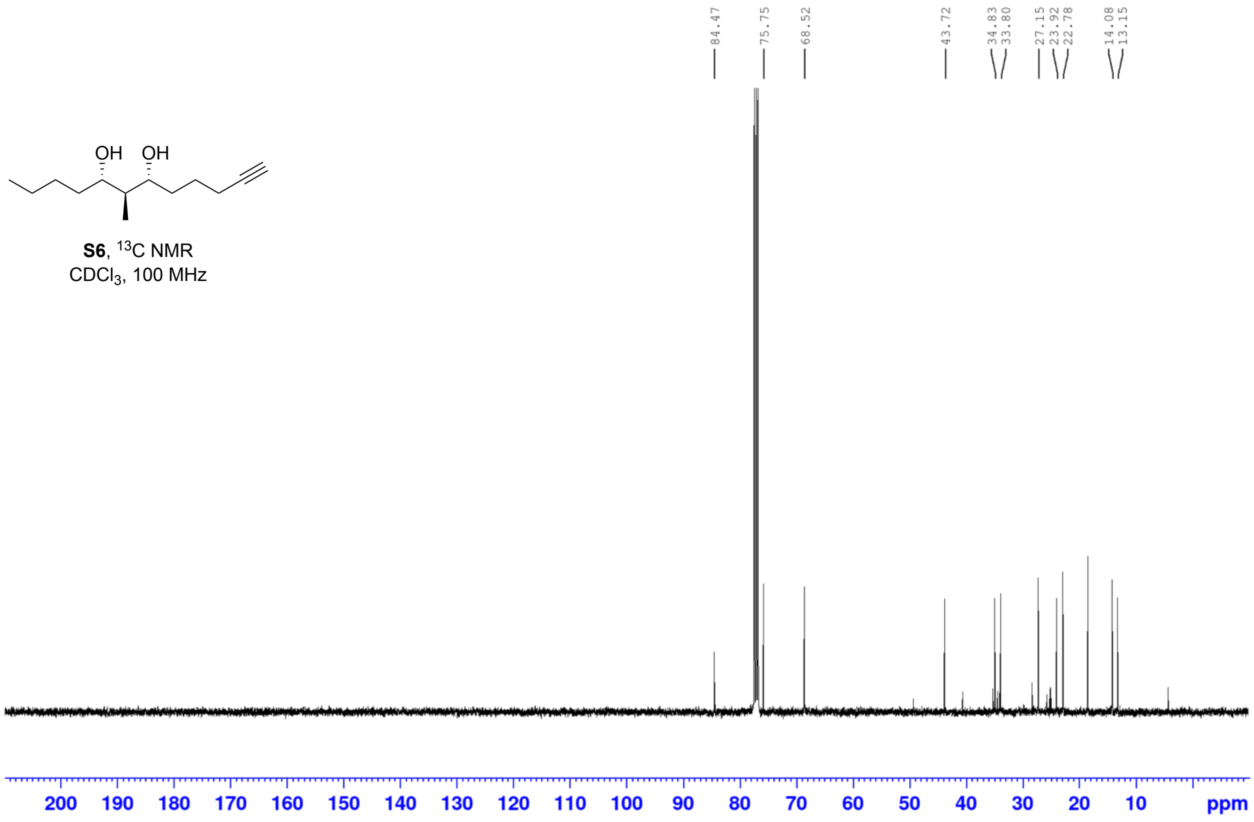

84.47 75.75 68.52 43.72 34.83 33.80 27.15 23.92 22.78 14.08 13.15
OH OH
S6, 13C NMR
CDCl3, 100 MHz
200 190 180 170 160 150 140 130 120 110 100 90 80 70 60 50 40 30 20 10 ppm

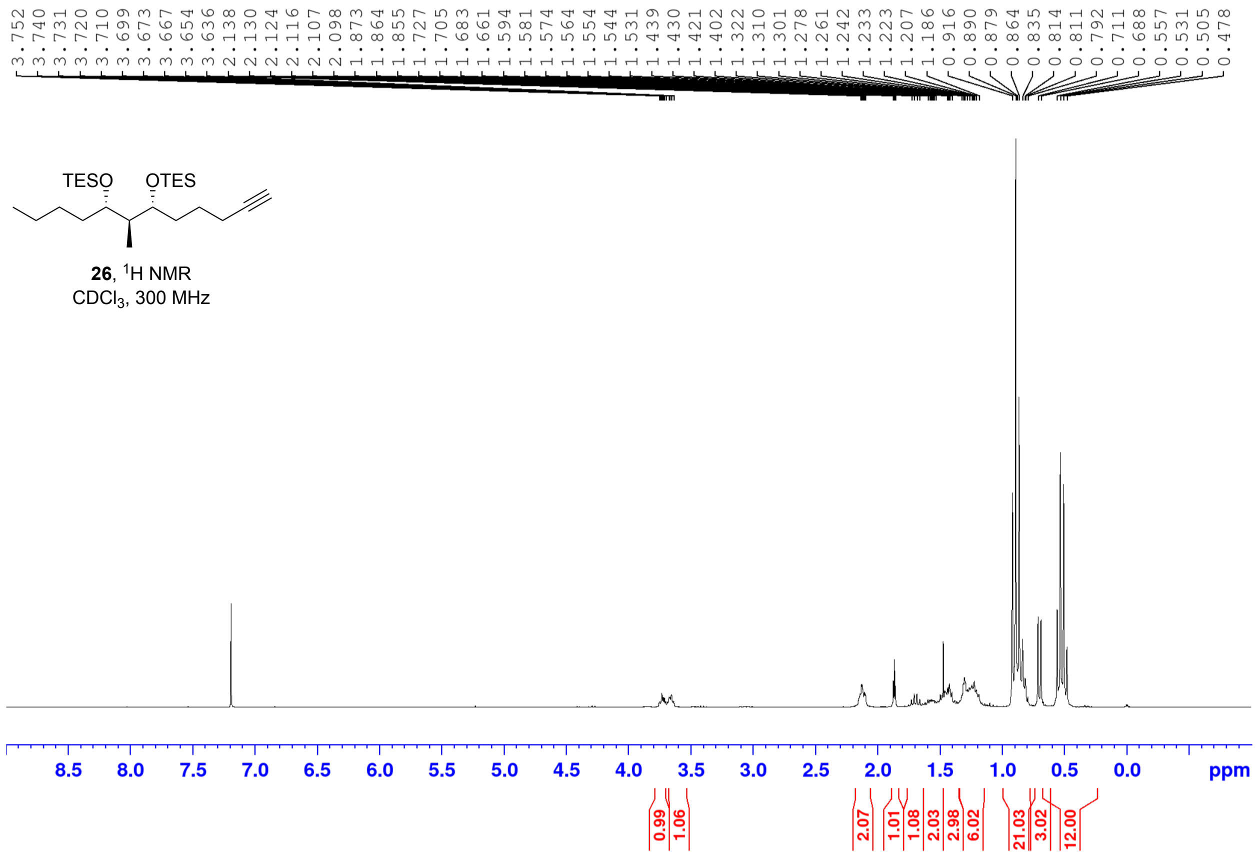

26, ^{1}H NMR
$CDCl_3$, 300 MHz

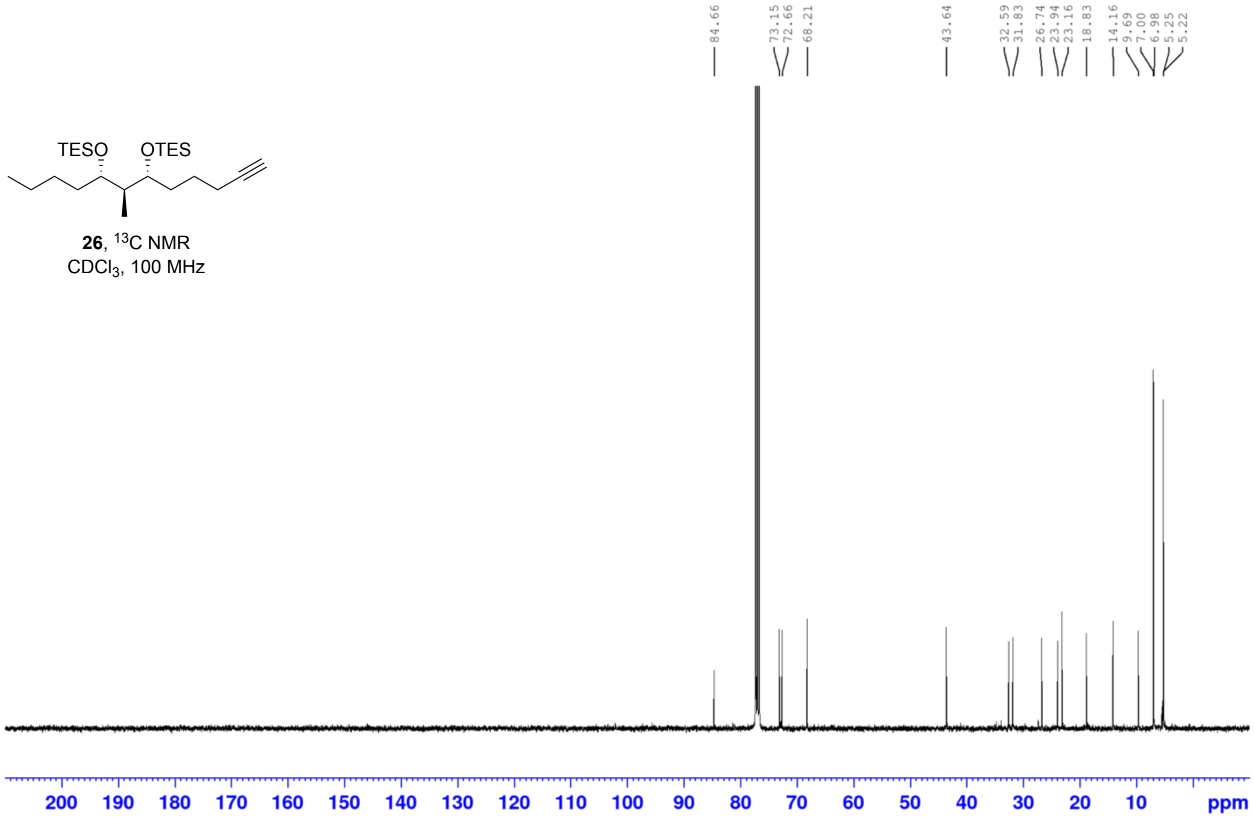

26, ^{13}C NMR
$CDCl_3$, 100 MHz

27, ^{1}H NMR
$CDCl_3$, 400 MHz

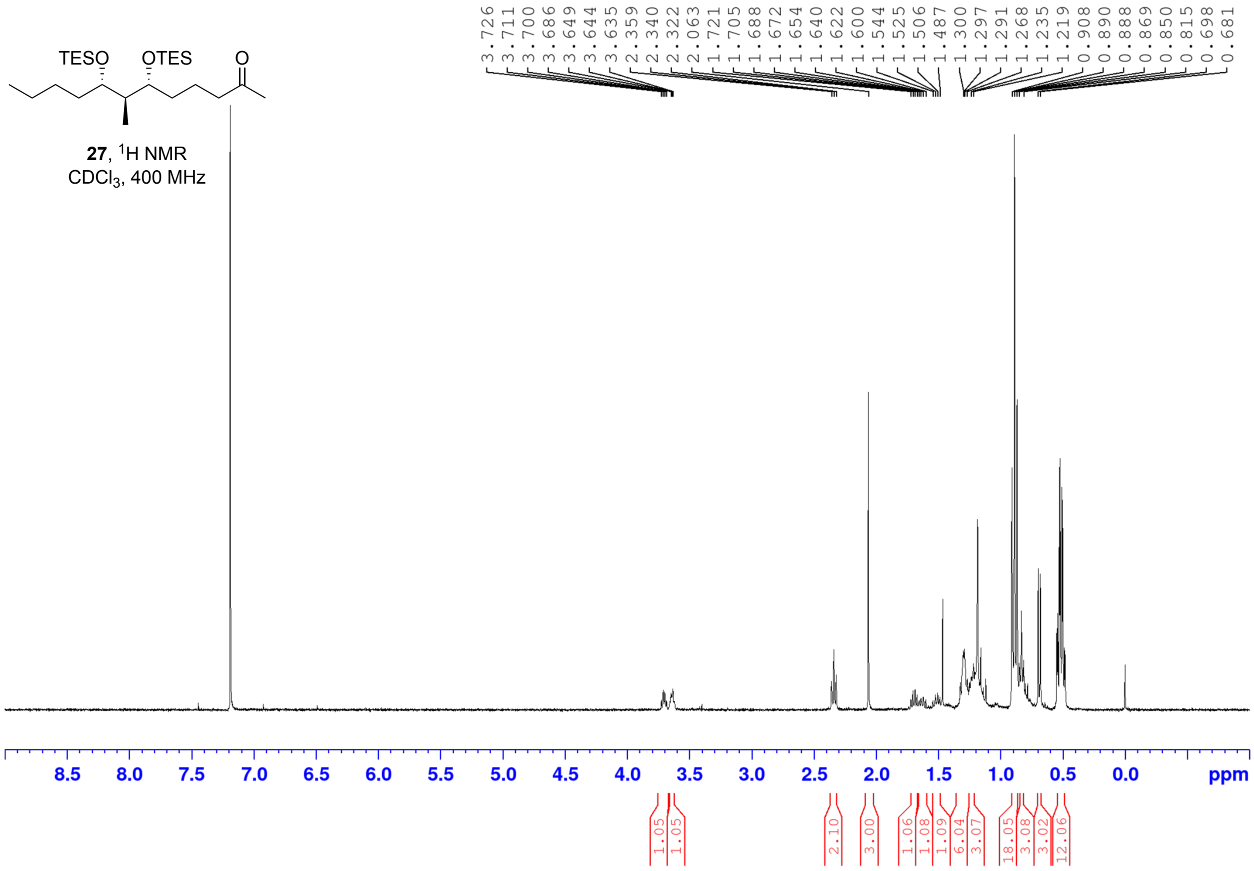

27, ^{13}C NMR
$CDCl_3$, 125 MHz

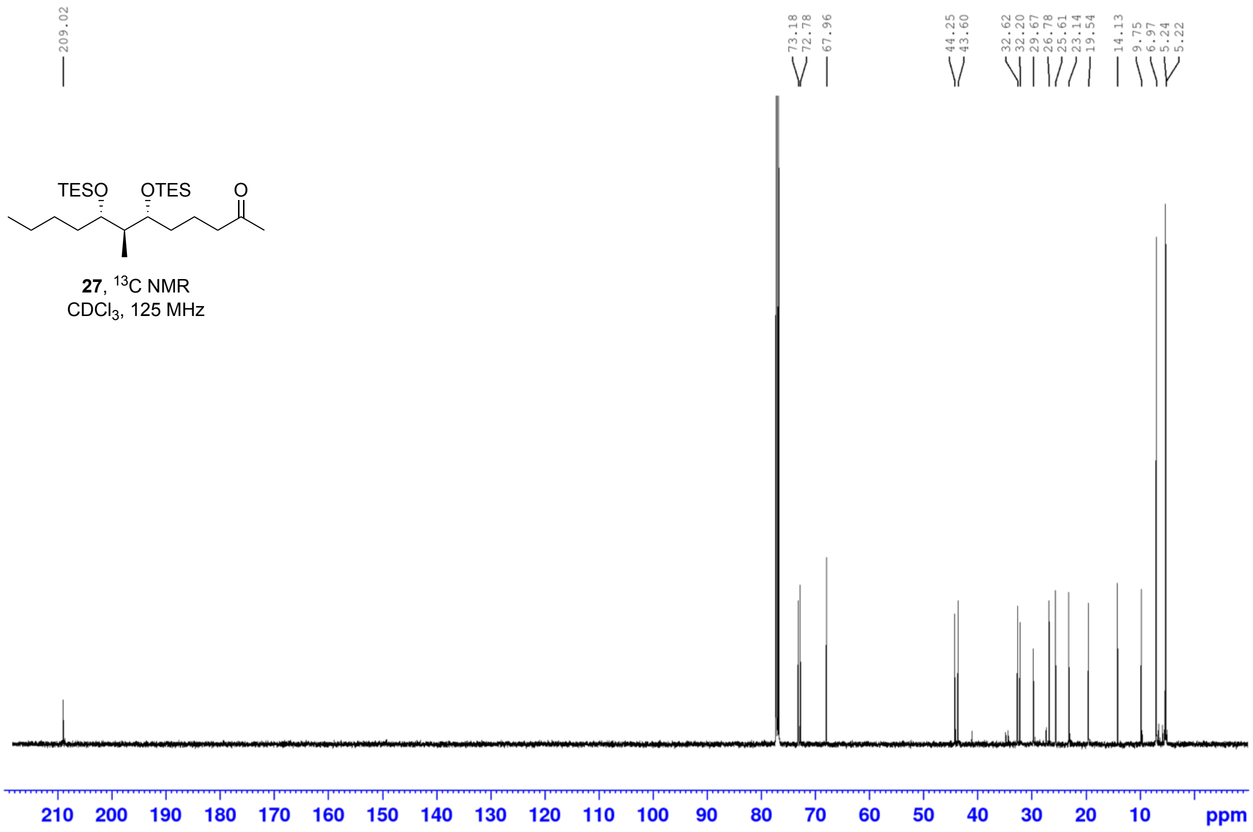

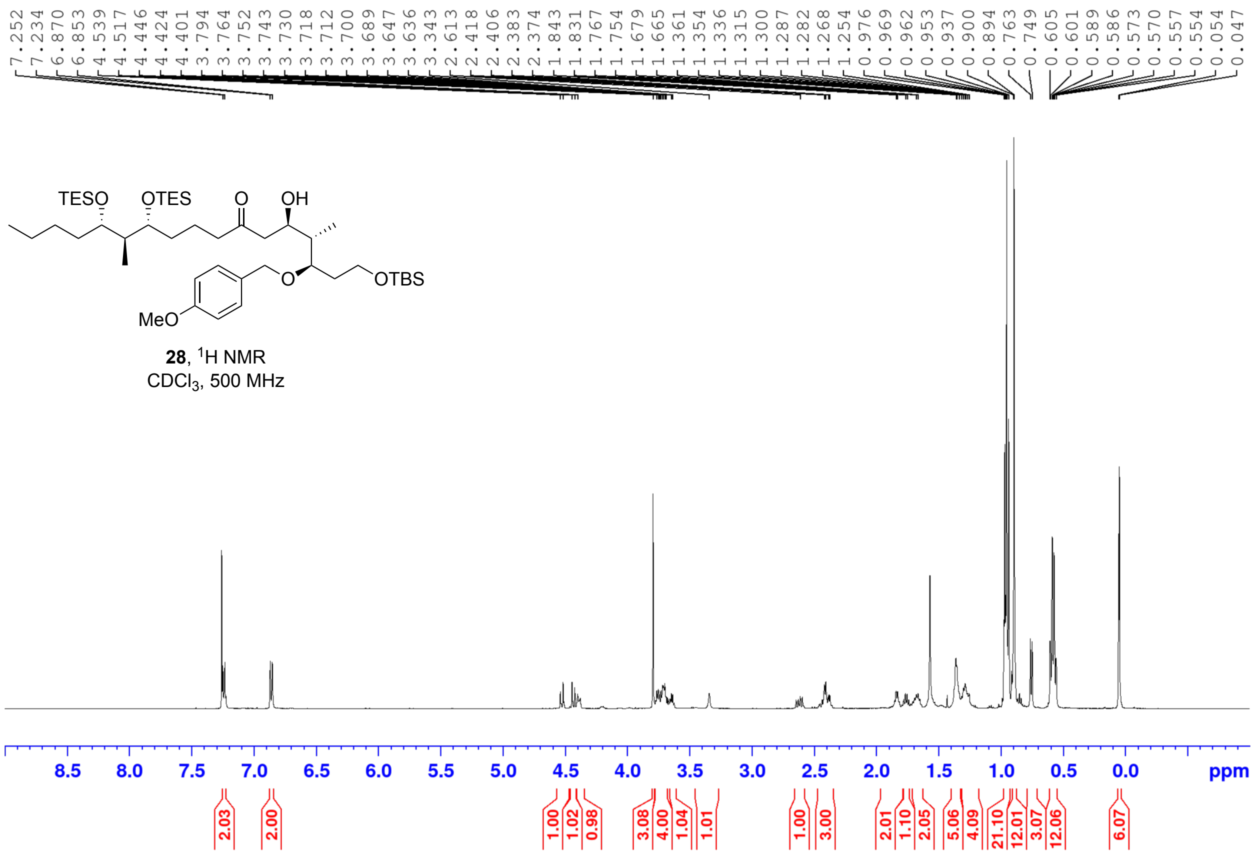

28, ^{1}H NMR
$CDCl_3$, 500 MHz

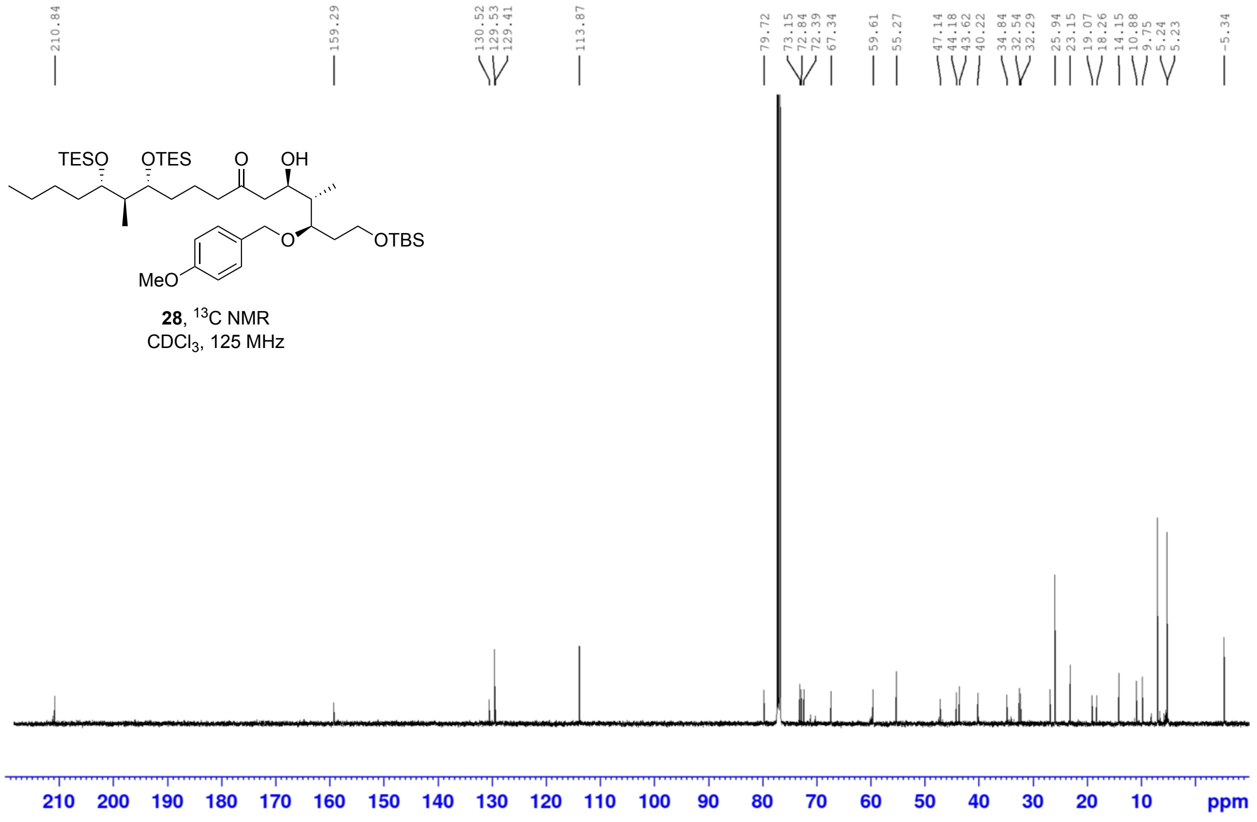

28, ^{13}C NMR
$CDCl_3$, 125 MHz

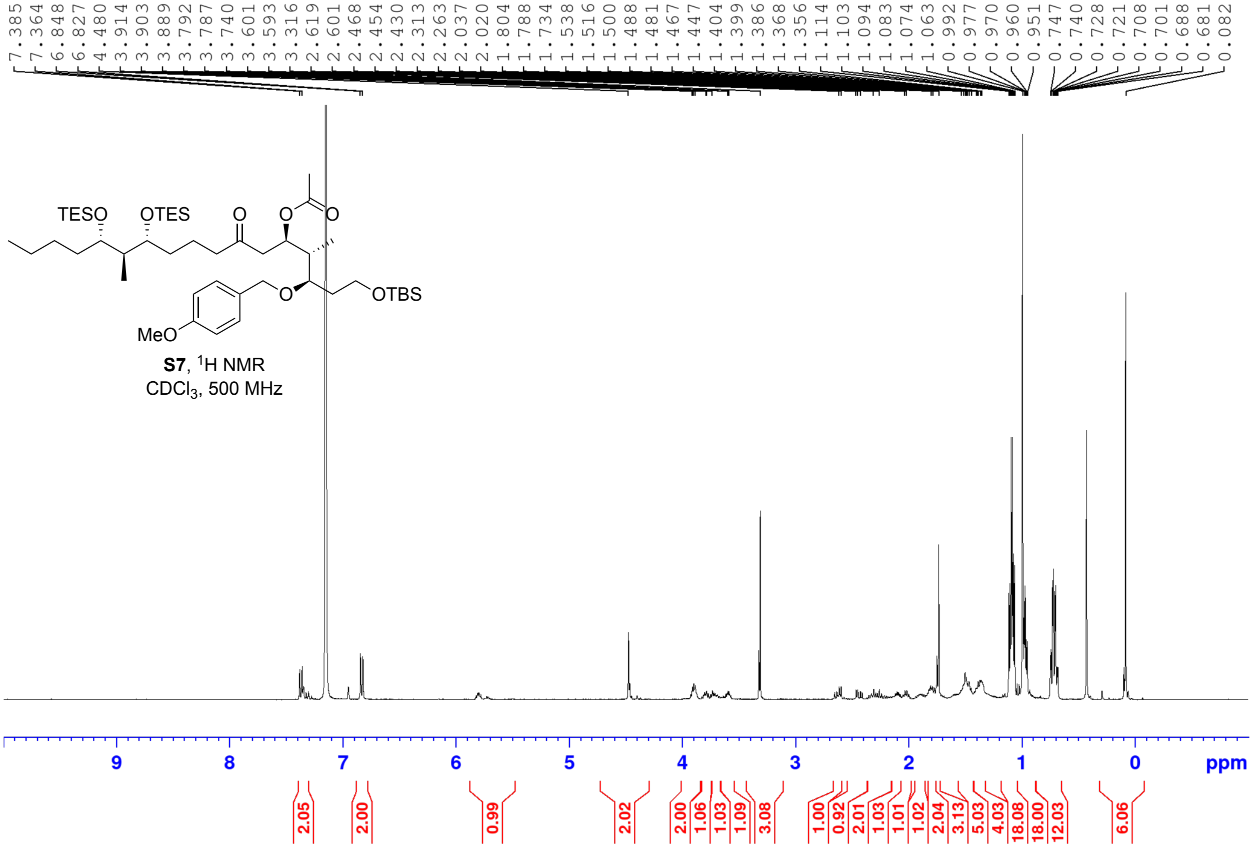
S7, 1H NMR
CDCl3, 500 MHz
TESO
OTES
OTBS
MeO
ppm

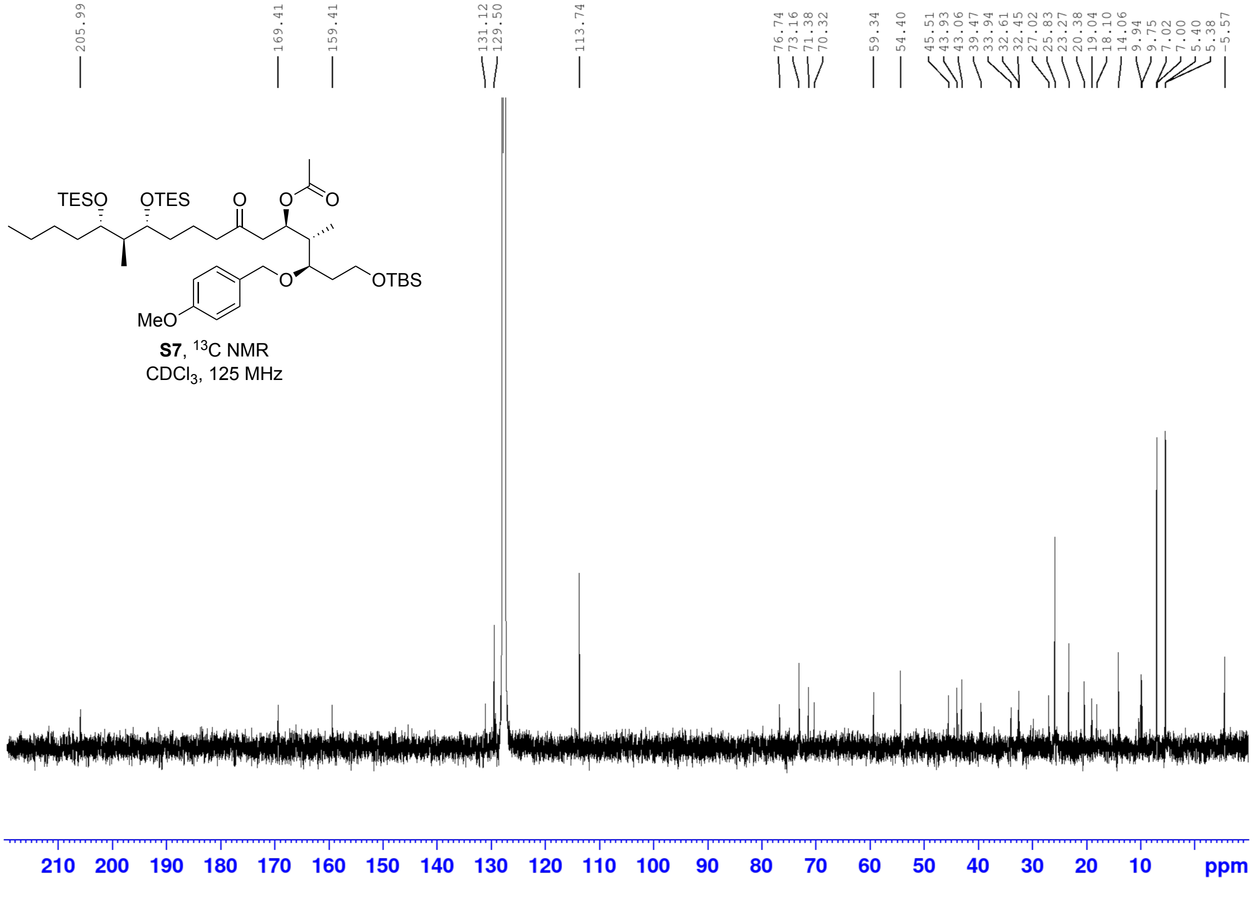
S7, 13C NMR
CDCl3, 125 MHz
TESO
OTES
OTBS
MeO
ppm

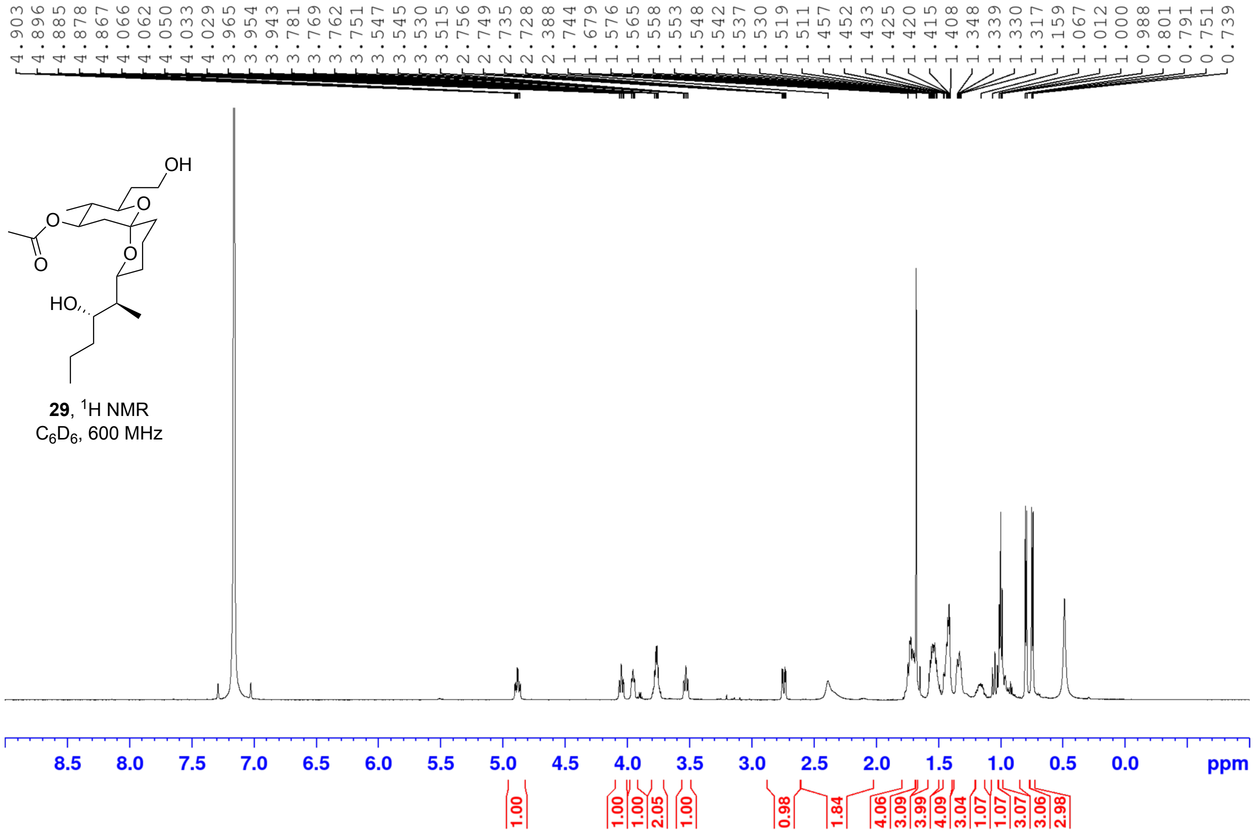
4.903 4.896 4.885 4.878 4.867 4.066 4.062 4.050 4.033 4.029 3.965 3.954 3.943 3.781 3.769 3.762 3.751 3.547 3.545 3.530 3.515 2.756 2.749 2.735 2.728 2.388 1.744 1.679 1.576 1.565 1.558 1.553 1.548 1.542 1.537 1.530 1.519 1.511 1.457 1.452 1.433 1.425 1.420 1.415 1.408 1.348 1.339 1.330 1.317 1.159 1.067 1.012 1.000 0.988 0.801 0.791 0.751 0.739
OH
O
O
O
O
HO
29, ^{1}H NMR
C_6D_6, 600 MHz
8.5 8.0 7.5 7.0 6.5 6.0 5.5 5.0 4.5 4.0 3.5 3.0 2.5 2.0 1.5 1.0 0.5 0.0 ppm
1.00 1.00 1.00 2.05 1.00 0.98 1.84 4.06 3.09 3.99 4.09 3.04 1.07 1.07 3.07 3.06 2.98

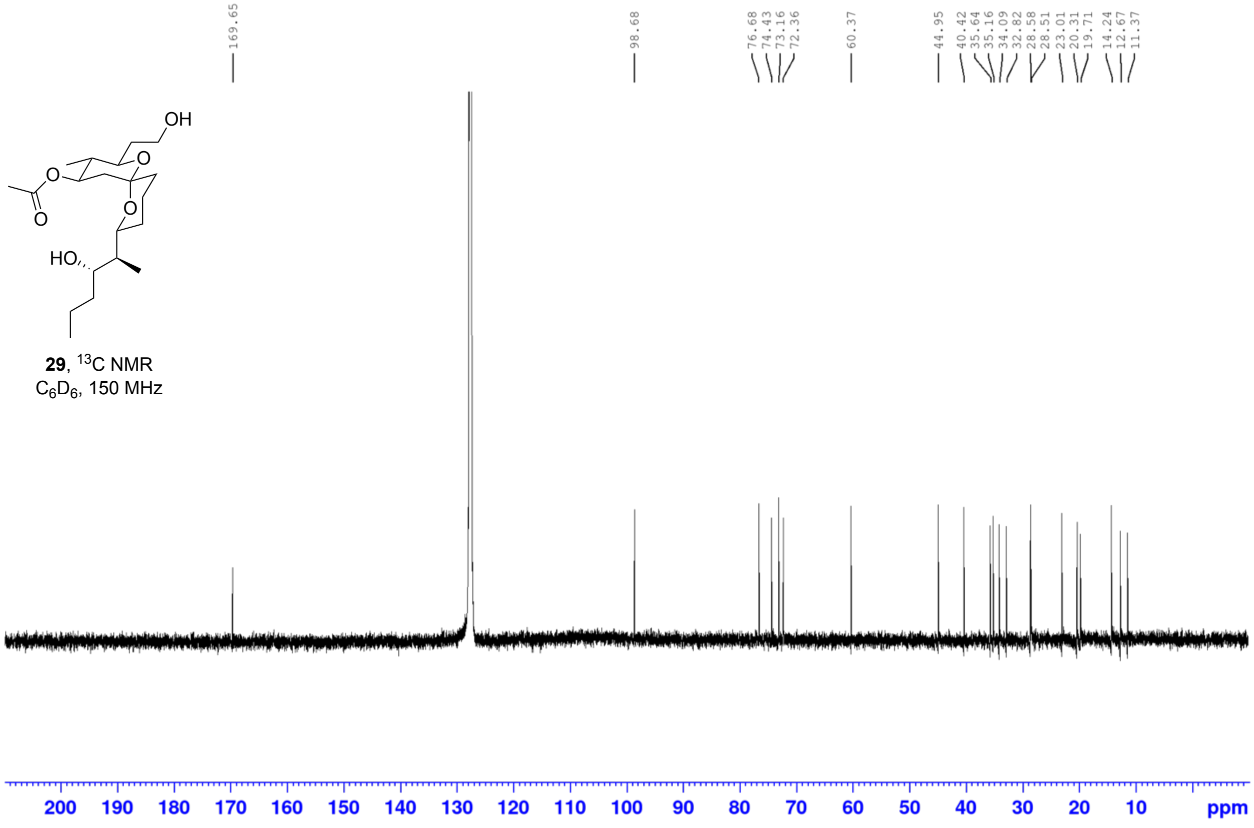
169.65 98.68 76.68 74.43 73.16 72.36 60.37 44.95 40.42 35.64 35.16 34.09 32.82 28.58 28.51 23.01 20.31 19.71 14.24 12.67 11.37
OH
O
O
O
O
HO
29, ^{13}C NMR
C_6D_6, 150 MHz
200 190 180 170 160 150 140 130 120 110 100 90 80 70 60 50 40 30 20 10 ppm

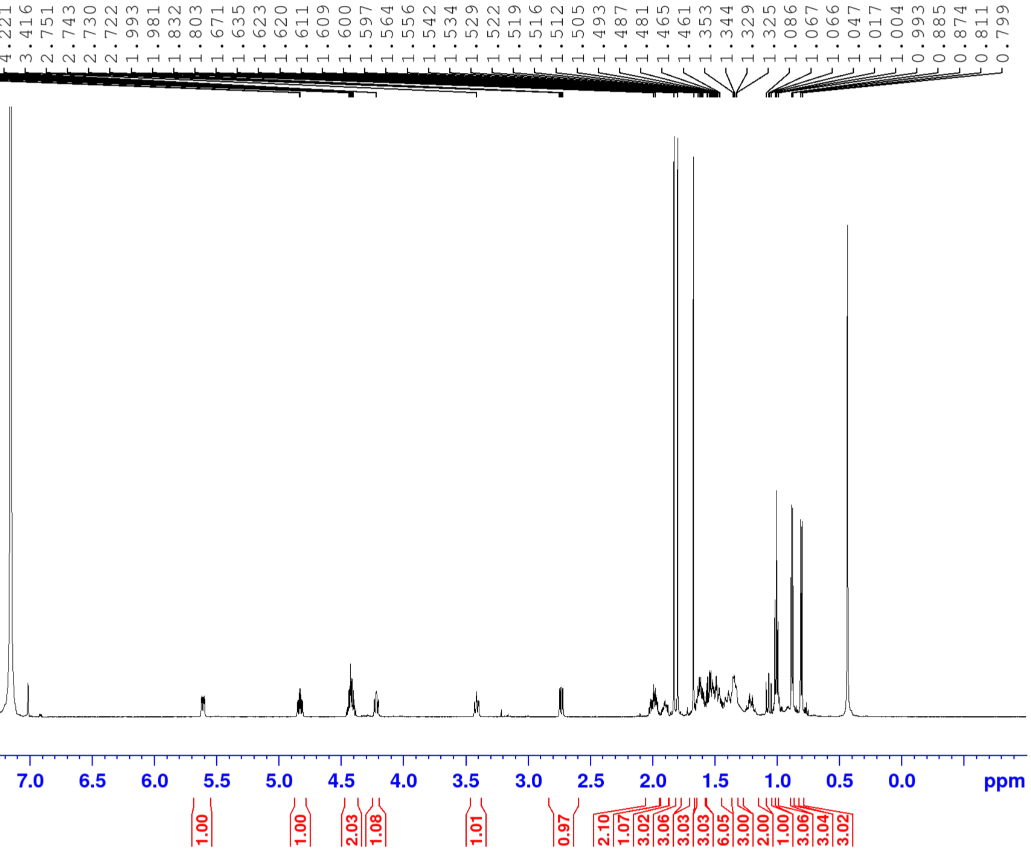

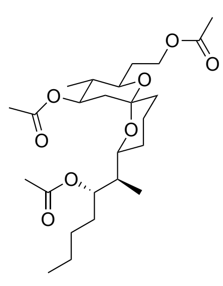

31, ^{1}H NMR
C_6D_6, 600 MHz

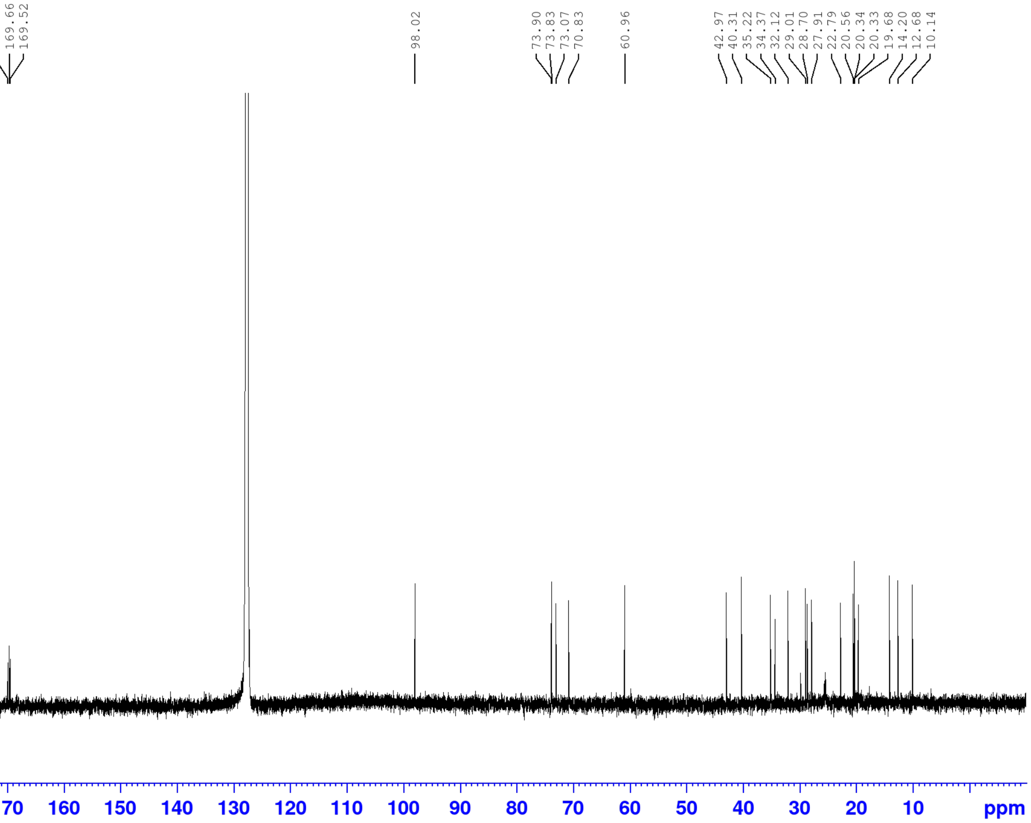

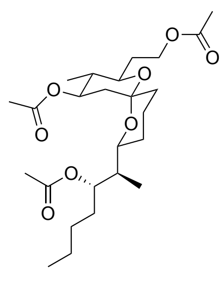

31, ^{13}C NMR
C_6D_6, 150 MHz

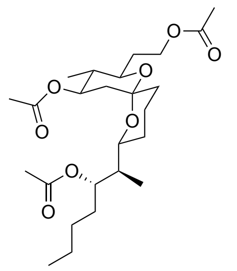

31, COSY
C_6D_6, 600 MHz

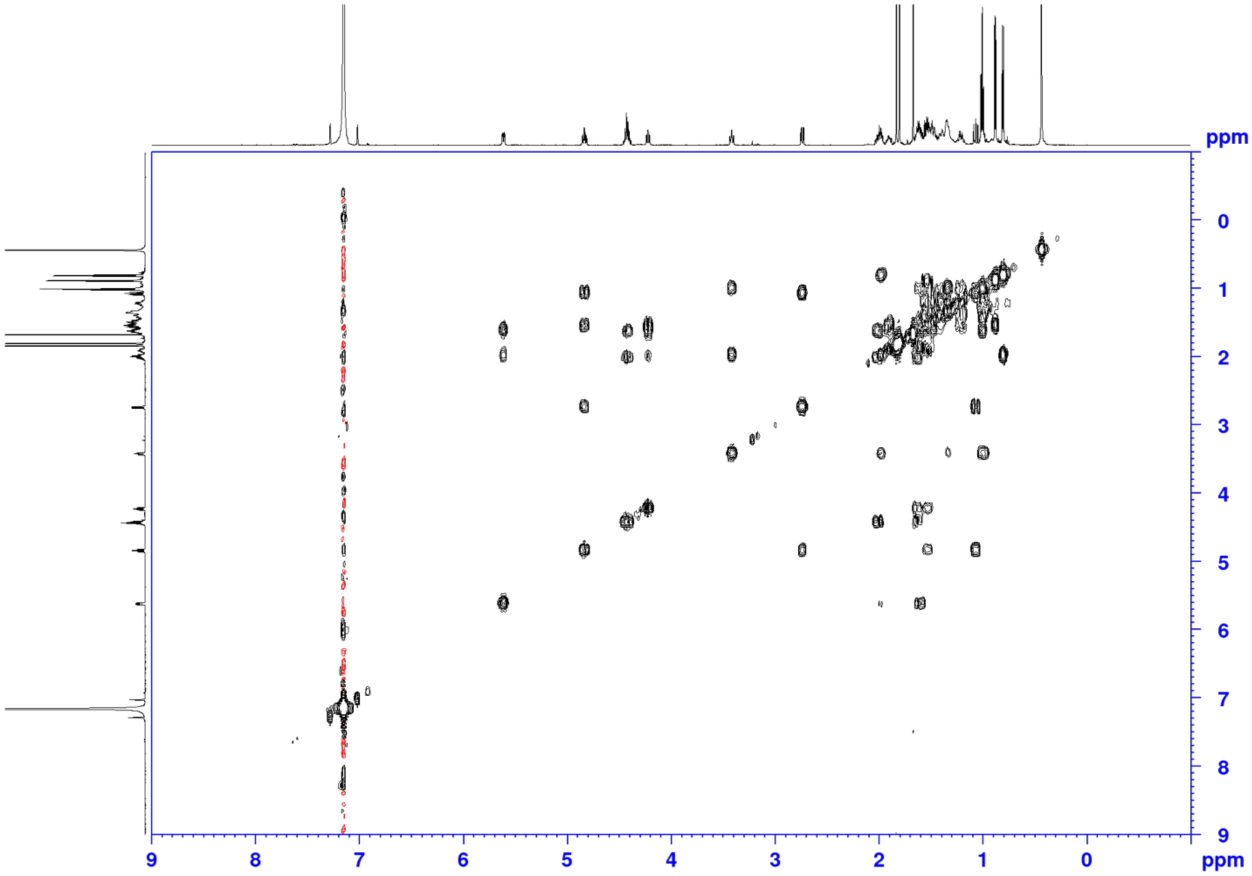

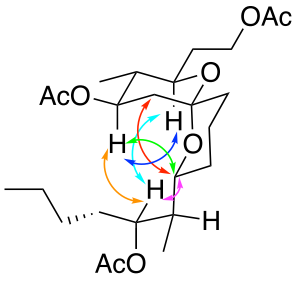

31, NOESY
C_6D_6, 600 MHz

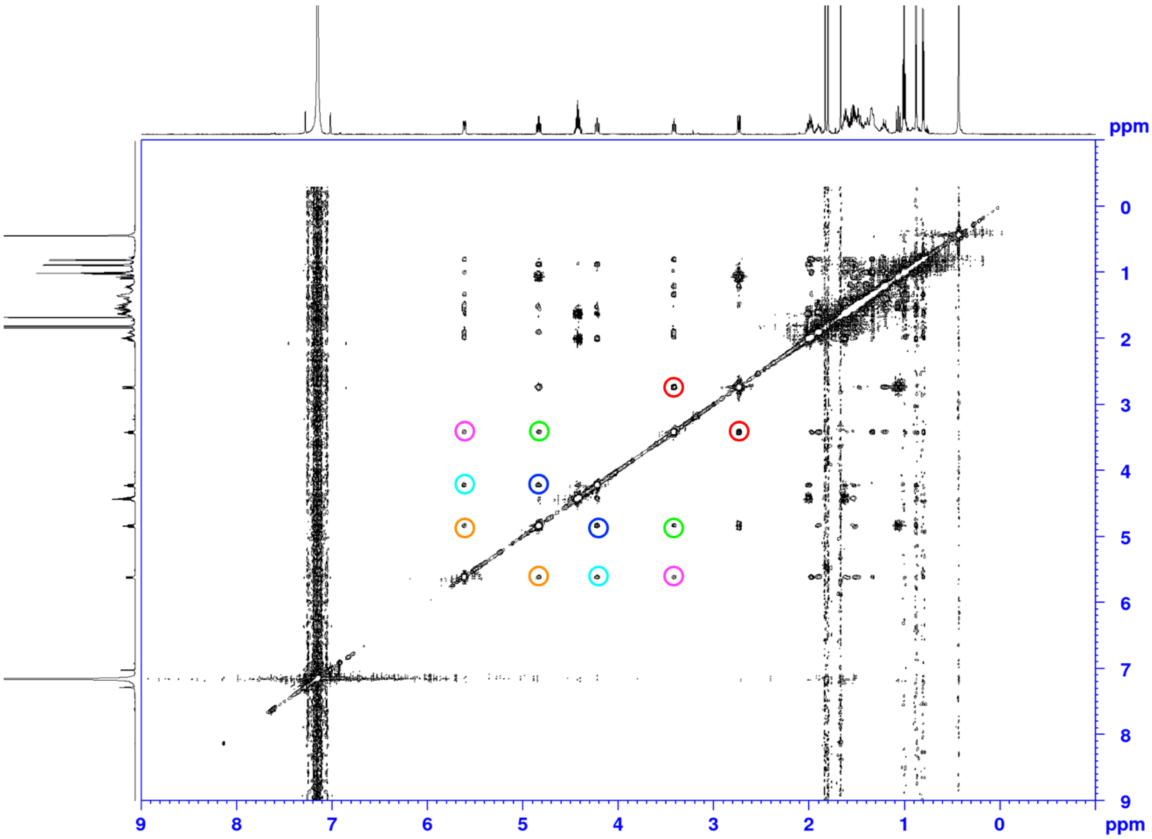

	**17**	**31**	Neaumycin B (isolated)[4]	Neaumycin B (synthetic)[5]
C25	4.12 ddd (*J* = 10.2, 9.2, 2.5 Hz)	4.22 ddd (*J* = 12.8, 9.3, 2.7 Hz)	3.85 dd (*J* = 11.5, 10.9 Hz)	3.95 dd (*J* = 12.1, 10.4 Hz)
C27	5.10 td (*J* = 16.2, 4.5 Hz)	4.83 td (*J* = 10.9, 4.5 Hz)	4.84 ddd (*J* = 12.8, 11.1, 4.6 Hz)	4.70 td (*J* = 10.8, 4.5 Hz)
C28 (eq)	2.83 dd (*J* = 12.7, 4.4 Hz)	2.74 dd (*J* = 12.8, 4.8 Hz)	2.62 d (*J* = 12.8, 4.9 Hz)	2.73 d (*J* = 13.0, 3.5 Hz)
C33	3.63 ddd (*J* = 11.2, 7.0, 1.9 Hz)	3.41 ddd (*J* = 13.7, 7.8, 2.0 Hz)	3.50 m	3.59 dd (*J* = 11.3, 7.1 Hz)
C34	2.00-2.05 m	1.94-2.04 m	1.61 m	1.70 m
C35	5.33 ddd (*J* = 8.5, 4.6, 4.0 Hz)	5.61 ddd (*J* = 10.7, 6.7, 2.0 Hz)	3.19 dd (*J* = 7.3, 2.0 Hz)	2.92 dd (*J* = 6.7, 3.2 Hz)

Table S1. Comparison of ^{1}H NMR chemical shifts between the isomers from this study, natural neaumycin B, and synthetic neaumycin B. Note that the spectra of the compounds from this study were taken with C_6D_6 as the solvent and the spectra of the neaumycin B structures were taken in $CDCl_3$.

References

[1] Han, X.; Floreancig, P. E. Spiroacetal Formation through Telescoped Cycloaddition and Carbon–Hydrogen Bond Functionalization: Total Synthesis of Bistramide A. *Angew. Chem., Int. Ed.* **2014**, *53*, 11075-11078.

[2] Paczkowski, R.; Maichle-Moessmer, C.; Maier, M. E. A Formal Total Synthesis of Dysidiolide. *Org. Lett.* **2000**, *2*, 3967-3969.

[3] Lim, J.; Chintalapudi, V.; Gudmundsson, H. G.; Tran, M.; Bernasconi, A.; Blanco, A.; Song, L.; Challis, G. L.; Anderson, E. A. Synthesis of the C1-C27 Fragment of Stambomycin D Validates Modular Polyketide Synthase-Based Stereochemical assignments. *Org. Lett.* **2021**, *23*, 7439-7444.

[4] Kim, M. C.; Machado, H.; Jang, K. H.; Trzoss, L.; Jensen, P. R.; Fenical, W. Integration of Genomic Data with NMR Analysis Enables Assignment of the Full Stereostructure of Neaumycin B, a Potent Inhibitor of Glioblastoma from a Marine-Derived *Micromonospora*. *J. Am. Chem. Soc.* **2018**, *140*, 10775-10784.

[5] Ding, J.; Smith, A. B., III Total Synthesis of the Reported Structure of Neaumycin B. *J. Am. Chem. Soc.* **2023**, *145*, 18240-18246.
